# Longitudinal linear combination test for gene set analysis

**DOI:** 10.1186/s12859-019-3221-7

**Published:** 2019-12-10

**Authors:** Elham Khodayari Moez, Morteza Hajihosseini, Jeffrey L. Andrews, Irina Dinu

**Affiliations:** 1 School of Public Health, University of Alberta, Edmonton, AB Canada; 2 School of Public Health, University of Alberta, Edmonton, AB Canada; 3 Department of Statistics, University of British Columbia | Okanagan Campus, Kelowna, BC Canada; 4 School of Public Health, University of Alberta, Edmonton, AB Canada

## Abstract

**Background:**

Although microarray studies have greatly contributed to recent genetic advances, lack of replication has been a continuing concern in this area. Complex study designs have the potential to address this concern, though they remain undervalued by investigators due to the lack of proper analysis methods. The primary challenge in the analysis of complex microarray study data is handling the correlation structure within data while also dealing with the combination of large number of genetic measurements and small number of subjects that are ubiquitous even in standard microarray studies. Motivated by the lack of available methods for analysis of repeatedly measured phenotypic or transcriptomic data, herein we develop a longitudinal linear combination test (LLCT).

**Results:**

LLCT is a two-step method to analyze multiple longitudinal phenotypes when there is high dimensionality in response and/or explanatory variables. Alternating between calculating within-subjects and between-subjects variations in two steps, LLCT examines if the maximum possible correlation between a linear combination of the time trends and a linear combination of the predictors given by the gene expressions is statistically significant. A generalization of this method can handle family-based study designs when the subjects are not independent. This method is also applicable to time-course microarray, with the ability to identify gene sets that exhibit significantly different expression patterns over time. Based on the results from a simulation study, LLCT outperformed its alternative: pathway analysis via regression. LLCT was shown to be very powerful in the analysis of large gene sets even when the sample size is small.

**Conclusions:**

This self-contained pathway analysis method is applicable to a wide range of longitudinal genomics, proteomics, metabolomics (OMICS) data, allows adjusting for potentially time-dependent covariates and works well with unbalanced and incomplete data. An important potential application of this method could be time-course linkage of OMICS, an attractive possibility for future genetic researchers.

Availability: R package of LLCT is available at: https://github.com/its-likeli-jeff/LLCT

## Introduction

Longitudinal designs are fast becoming a key instrument in genetics studies, as they advance our understanding of disease progression and underlying biological mechanism. Longitudinal studies can provide information regarding age of onset along with time-varying covariates that may aid in our understanding of a complex disease. A primary concern of these study designs is to find a proper analysis method which deals best with the correlation structure imposed by longitudinal data. Within-subject correlation in the context of high dimensional data cannot be addressed by traditional statistical analysis methods.

In the past two decades, there has been an increasing interest in microarray studies which has triggered rapid advances in microarray data analysis methods. From 2001, a considerable amount of literature has been published on methods of Individual Gene Analysis (IGA) [1] and Gene Set Analysis (GSA) [2–5]. Majority of these studies have proposed enrichment methods for binary and categorical phenotypes. Little attention has been paid to developing the methods for other phenotypes, especially longitudinal ones. The current paper contributes to filling this gap by proposing a longitudinal linear combination test (LLCT).

A frequent practice to deal with longitudinal phenotypes in genetics studies is to simply average across the multiple measurements. With this approach, the temporal variation of the phenotype is discarded and part of the information is lost [6]. To the best of our knowledge, the only GSA method developed to analyze longitudinal phenotype is the Pathway Analysis via Regression (PAVR) method proposed by Adewale et al. [7]. This method utilizes regression modelling to analyze binary, multi-class, continuous, count, rate, survival and longitudinal data and adjusts the results for potential covariates. In this method, the measure of association of a specific gene set with the phenotype is a sum of squares of Wald statistics from regression models fitted on the phenotype against the individual genes in the pathway of interest. We will compare this method with LLCT and discuss its limitations, later in this manuscript.

Our goal is to develop a statistical method which not only tackles the limitations of available methods, but addresses challenges of complex designs in recent microarray studies. The main function of this method is to recognize differentially expressed gene sets associated with a phenotype trajectory over time. It is also applicable to family-based study designs when the subjects are not independent. A generalization of this method can handle time-course microarray studies and identify gene sets with significantly different expression patterns over time.

Longitudinal microarray studies may wish to consider the trajectories of both phenotypes and gene expressions. In time-course microarray studies, arrays are collected repeatedly over time, allowing one to examine the dynamic behavior of gene expressions. GSA methods for time-course gene expressions have received more attention than GSA methods for repeated measurements of phenotypes. These methods are exploratory in nature, clustering genes to co-expressed groups [8]. Unfortunately, this development is not sufficient to address biologists’ concerns about the association of gene expressions trajectories with one or more specific covariate(s). Many procedures have been proposed for time-course microarray experiments to test if specific genes exhibit different expression profiles significantly associated with covariates. ANOVA-based methods [9, 10] and regression-based approaches are very popular in this field. Linear Mixed Models (LMM) or Generalized Estimating Equations (GEE) are more mature statistical models accommodating the correlations between repeated measurements. However, they are not directly applicable, as the time-course expression data is often collected for a large number of genes, but only for few subjects. To deal with the high dimensionality problem, Turner et al. [11] modeled the genes separately and then rescaled the data using Variance Inflation Factor (VIF) estimates to accommodate the correlation between the genes within gene sets. LMMs were also used in the methods developed by Hejblum et al. [12], Zhang et al. [13], and Conesa et al. (maSigPro method) [8], but they only work with categorical predictor variables. Our proposed method, LLCT can handle both categorical and continuous predictors.

Family-based data is another type of complex design in microarray studies. Family-based study designs are advantageous compared to studies of unrelated subjects in terms of lower genomic or phenotypic heterogeneity. Also, we are more likely to detect any significant effect when we observe multiple copies of the significant effects in a family [14]. Over the past few decades, study designs incorporating information from related subjects have resulted in better scientific interpretations [15].

LLCT is a GSA method. Incorporating information about the group of genes which are linked via biological pathways, LLCT aims to discover gene sets associated with the phenotype trajectories. These biological pathways, or a-priori defined gene sets, are archived in online databases: The Cancer Genome Atlas (TCGA) [16], Gene Expression Omnibus (GEO) [17], Keyoto Encyclopedia of Genes and Genomes (KEGG) [18], BioCarta [18], Molecular Signature Database of the Broad Institute [19] . Although imposing additional complexity into the analysis, this feature of LLCT is biologically very appealing. In contrast to IGA, GSA works based on a biologically realistic assumption that the genes are not independent and a cell’s function can be accomplished by differential expression of a group of genes, even if all of them show only weak to moderate changes [20].

LLCT is a self-contained method. Methodological reviews on GSA emphasize the distinction between self-contained and competitive GSA. A competitive method employs gene permutation to test whether the association between a gene set and the outcome is equal to those of the other gene sets (so-called “Q1 hypothesis” [21]). A self-contained method employs subject permutation to test the equality of the two mean vectors of gene set expressions corresponding to the two groups (so-called “Q2 hypothesis” [21]). Since competitive methods have been widely criticized for their inability to take care of the correlation within gene sets, we focus here on developing a self-contained method testing the Q2 hypothesis.

## Results

### Simulation study

**Fig. 1:**
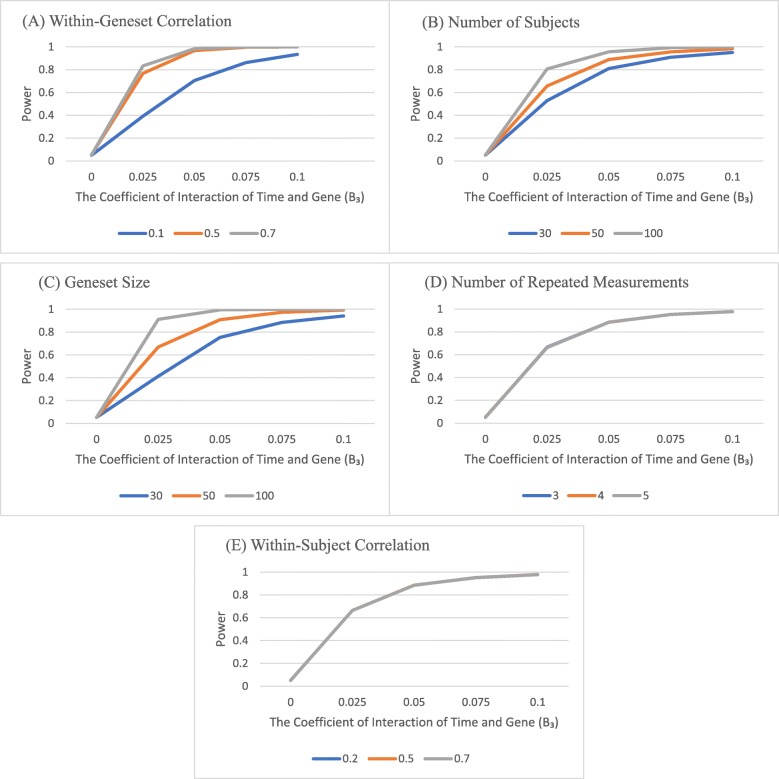
Calculation of the power of LLCT using simulated data generated with different within-geneest correlation (**a**), sample size (**b**), geneset size (**c**), number of repeated measurements (**d**) and within-subject correlation (**e**). Type I error is set at 5%. For each plot, the simulation variables except the one mentioned on the title varies but remains comparable among the curves

**Fig. 2:**
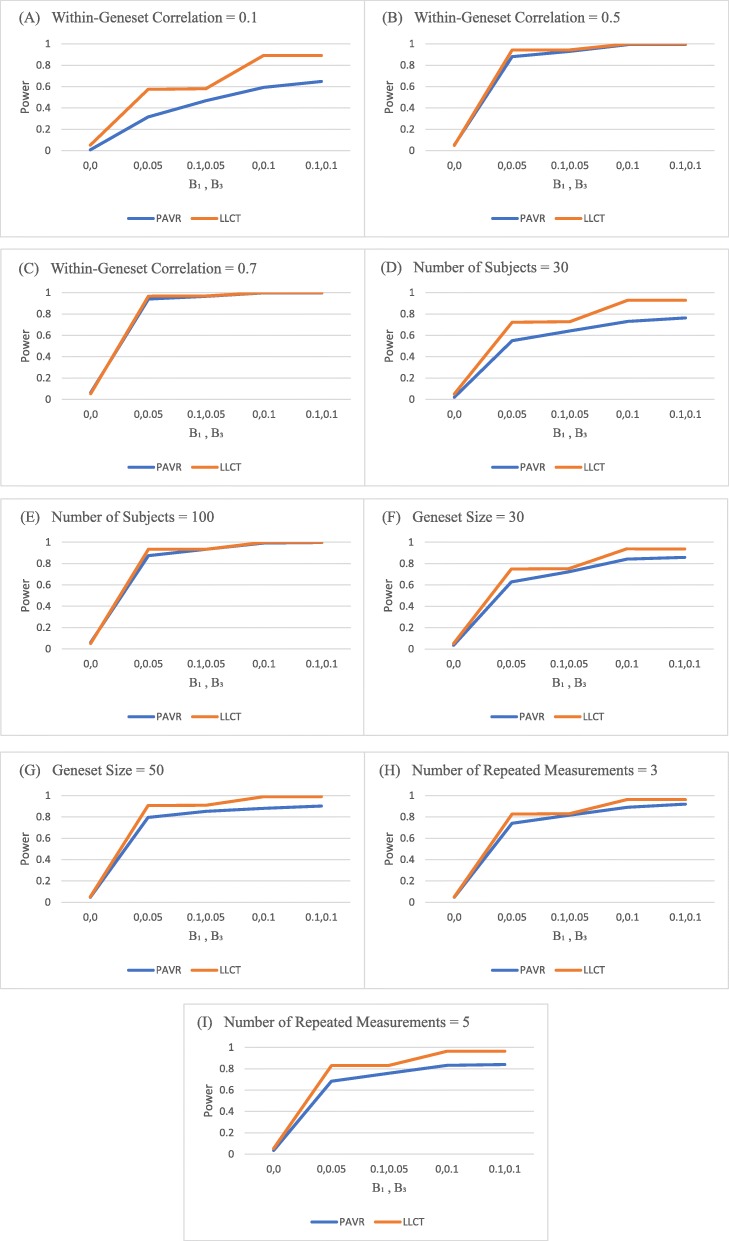
Comparison of the powers of LLCT method and the method of pathway analysis via regression (PAVR) proposed by Adewale et a1. using simulated data generated with different within-geneset correlation (**a, b** and **c**); different sample size (**d** and **e**); different geneset size (**f** and **g** and **h**) and different number of repeated measurements (**h** and **i**). Β_1_ denotes the gene effect and Β_3_ denotes the gene effect over time referring to eq. 7

We present here results of our simulation study on LLCT performance. Figure 1 shows the power of LLCT analyzing diverse sets of data, simulated by considering different within-gene-set and within-subject correlations, sample and gene set sizes, and the number of repeated measurements. For each plot, the type I error was constant at 0.05 and the simulated data were similar for all characteristics except the one mentioned at the top of the plot. The power was calculated at the presence of different Β_3_ values, determining the effect of each gene within specific gene set over time. The power of LLCT increased by higher within-gene-set correlation, sample size and gene set size (Fig. 1a-c). However, it remains unaffected by within-subject correlation and number of repeated measurements (Fig. 1d-e).The power of LLCT and PAVR were compared in Fig. 2, where we let within-gene-set correlation, sample size, gene set size, and number of repeated measurements change. PAVR does not distinguish between the gene effect and the gene effect over time. Therefore, two parameters of Β_1_ and Β_3_ were set at different values (other than zero for both) to define alternative hypotheses for this method. However, the power of LLCT was consistent over different values of Β_1_ and altered by Β_3_ only. For small within-gene-set correlation values (*ρ* < 0.5), LLCT significantly outperformed PAVR. However, as the within-gene-set correlation increased, the power values of PAVR and LLCT became closer (Fig. 2a, b, c). Comparing with LLCT, PAVR performed poorly when the sample was small (Fig. 2d, e). Furthermore, different gene set sizes did not make a considerable difference between the methods’ powers (Fig. 2f, g). LLCT exhibited a better ability in dealing with large number of repeated measurements over time (Fig. 2h, i).

### Application 1: blood pressure

Hypertension affects more than a quarter of the world’s adult population [22] annually and adds a significant burden on healthcare systems. Long-term hypertension damages heart, kidney, brain, large blood vessels and retinal vessels [23] and explains about half of stroke and ischaemic heart diseases worldwide. Despite this high health risk, hypertension is unknown for more than 30% of patients, untreated for 50% of them, and uncontrolled for 75% [24].

Blood pressure is known as a highly-heritable complex trait [25] regulated by multiple environmental and genetic factors. The importance of understanding the genetics mechanism of blood pressure on identification of therapeutic and prevention targets has been emphasized in studies examining the variation of effectiveness of antihypertensive medications on different ancestral groups [26].

Hypertension is developed by small contributions of a large number of genes, whose effects may be hard to detect. Facing this challenge, most studies on hypertension genetics failed to reach replication. Traditional statistical approaches suffer from inferential limitations in genetic studies, largely due to small sample sizes. Novel methodologies are being developed to address this issue.

Genetic Analysis Workshops (GAWs) are designed to evaluate the performance of different statistical methods applied on high density genotype. Among them, GAW13 [27], GAW16 [28], GAW18 [29] and GAW19 [30] have focused on analysis of longitudinal datasets. GAW19 [30], the focus of our work, is based on data from San Antonio Family Heart Study (SAFHS), conducted to investigate the genetics of cardiovascular disease in Mexican-Americans. GAW19 researchers were divided into different teams to work on heterogeneous statistical methods dealing with longitudinal datasets. For analysis of gene expressions, these teams independently worked on different areas of individual or pathway gene analysis, unrelated or family-based analysis and joint or separated analysis of phenotypes. However, utilizing heterogeneous statistical methods prevented them from replicating their findings.

The subjects of SAFHS were born in a large, multi-generational family and their stated pedigree relationships were verified. The transcriptional profile data of 647 people was recorded, including 16,383 gene expression measurements, for each individual. For each subject, systolic blood pressure (SBP), diastolic blood pressure (DBP), hypertension status (HTN), use of antihypertensive medications, and smoking status were measured at four time points and the subjects’ sex and age were recorded. By applying the proposed method to this family-based data, we detected differentially expressed gene sets significantly associated with blood pressure trajectories over time. We analyzed this real data set and considered DBP, SBP, pulse pressure (PP) (defined as PP = SBP − DBP), and hypertension (defined as blood pressure ≥ 140/90 mmHg) as the outcome variables.

We first analyzed the unrelated subjects by selecting subjects with no shared parents. In this part of analysis, the repeatedly measured expressions of 10,072 genes for 64 subjects, belonging to 5898 gene sets were examined by LLCT for unrelated subjects. The gene sets are defined by Gene Ontology database. The size of gene sets varied from 2 to 1417 with median of 22.

In the second part of analysis, 647 related subjects in 17 family clusters were analyzed. The size of families varied from 21 to 62 with the median of 31. The total number of 10,072 genes contributing in 5907 pathways was tested by LLCT for related subjects. Table 1 shows the characteristics of the related and unrelated subjects.

**Table 1:** Summary information (mean (standard deviation)) of covariates and outcomes at different time points: GAW19 application, studies of related and unrelated subjects

	Age	Antihypertensive Medication	Smoking Status	Systolic Blood Pressure (SBP)	Diastolic Blood Pressure (DBP)	Hypertension Status (HTN)
Related Subjects						
First visit	39.58 (16.88)	0.1 (0.3)	0.23 (0.42)	121.73 (18.98)	71.48 (9.99)	0.18 (0.39)
Second visit	42.76 (15.93)	0.19 (0.39)	0.18 (0.39)	124.96 (19.34)	71.94 (10.01)	0.28 (0.45)
Third visit	46.34 (15.10)	0.29 (0.45)	0.2 (0.4)	125.21 (18.04)	70.73 (10.02)	0.36 (0.48)
Forth visit	50.88 (12.76)	0.43 (0.5)	0.11 (0.32)	128.24 (17.63)	77.76 (11.06)	0.52 (0.5)
Unrelated Subjects						
First visit	53.84 (14.77)	0.22 (0.42)	0.25 (0.43)	130.3 (23.36)	72.96 (9.48)	0.37 (0.48)
Second visit	58.26 (12.30)	0.36 (0.48)	0.11 (0.32)	135.01 (20.17)	72.34 (10.09)	0.59 (0.49)
Third visit	59.52 (10.85)	0.53 (0.50)	0.17 (0.38)	130.46 (19.24)	69.14 (9.74)	0.59 (0.49)
Forth visit	62.16 (9.26)	0.63 (0.49)	0.06 (0.25)	135.5 (23.44)	77.06 (15.4)	0.71 (0.46)

The test of association was conducted after adjustment for either smoking status or antihypertensive medications intake. As some subjects were measured for two times only, the method was unable to adjust for both time-dependent covariates at the same time, unless we restricted our subjects to those with more than 2 measurements.

LLCT was used to find the gene sets whose expressions are significantly associated with the outcome(s) and calculated 5989 *p*-values for testing the gene sets in unrelated study and 5907 p-values for analysis of family-based dataset. Table 2 shows the number of significant gene sets in testing each outcome and each dataset separately. The pathways that were significantly associated with both pulse pressure and linear combination of SBP and DBP, after adjusting for antihypertensive medication consumption, were selected and shown in Additional file 2 and Additional file 3. Exposure to blood pressure medication, compared to smoking, showed more considerable effect in changing SBP and DBP trajectories and the best model is the one adjusting for this effect.

**Table 2:** The number of significant gene sets found by LLCT at different levels of confidence, testing a variety of outcomes and datasets

Datasets	Type I Error	SBP	DBP	SBP& DBP^a^	SBP-DBP^b^	HTN
Adjusted for smoking status						
Related Subjects	1%	30	23	20	73	65
	5%	170	135	141	360	321
	10%	255	278	310	434	392
Unrelated Subjects	1%	12	3	5	27	5
	5%	136	39	60	389	82
	10%	408	78	245	735	162
Adjusted for antihypertensive medications						
Related Subjects	1%	98	13	63	127	12
	5%	402	127	271	541	99
	10%	413	242	390	614	159
Unrelated Subjects	1%	17	3	11	17	2
	5%	142	60	86	116	22
	10%	465	75	186	382	88
No Adjustment						
Related Subjects	1%	18	17	14	43	54
	5%	158	141	122	259	327
	10%	263	273	277	386	417
Unrelated Subjects	1%	9	2	3	17	2
	5%	234	37	70	273	71
	10%	537	68	231	682	168

^a^The multiple analysis of systolic and diastolic blood pressure. In this analysis, the outcome is a linear combination of SBP and DBP with the highest association with the linear combinations of gene expressions

^b^Pulse pressure which is the difference between systolic and diastolic blood pressures

In Additional file 2 and Additional file 3, the gene sets were classified based on their shared ancestral categories, derived from Gene Ontology Tree. A few descendent pathways of immune system process, cellular response to stimulus, cell communication, cellular metabolic process, multi-organism cellular process, multi-cellular organism process and metabolic process has been found to be significant in the analyses of related and unrelated subjects. Cell differentiation, cell activation, cell cycle, cellular component organization or biogenesis, biological regulation, system development, localization, metabolic process and response to stimulus are other parental classes of biological processes with significant descending pathways in the analysis of the related dataset only. In addition to these biological processes, few significant pathways in major classes of molecular function and cell components were found significant. The family-based analysis is expected to result in more accurate findings, as it works on the larger database.

Blood pressure is a complex phenotype that is controlled by multiple biological processes, multiple molecular functions and multiple cell components. Comparing the results of the analysis of multiple phenotypes, pulse pressure displayed higher level of robustness and was less affected by covariates. Also, HTN failed to reflect the changes of SBP and DBP and mostly failed to agree with the analysis results of other phenotypes. From a statistical perspective, the result of HTN analysis is limited because the information is lost by dichotomizing the continuous variables. Also, many biological studies doubted the reliability of this one-size-fits-all stratification scheme [31]. The other noteworthy finding of this study was the difference between SBP and DBP trajectories in their association with gene expressions. There were larger number of pathways associated with SBP compared to DBP. This underlines the sensitivity of SBP, as a blood pressure measurement, to gene expression alterations.

By discussing the list of significant pathways in Additional file 2 and Additional file 3, insights can be gained into the genetics of hypertension. However, we admit that an in-depth biological interpretation of the findings is beyond the scope of this manuscript. Below, we will discuss some processes underlying hypertension, whose presence was supported by more than one significant pathway in LLCT analysis. We also provided the prioritization score, indicating the proportion of gene sets with smaller *p*-values [32], for each significant pathway. These scores were calculated using the p-value ranks based on LLCT analysis of bivariate SBP and DBP after adjustment for medications.

#### Regulation of smooth muscle contraction by signal transduction

Recent developments in blood pressure studies have highlighted the importance of the regulation of vascular smooth muscle contraction and vascular tone on the regulation of blood pressure. Young blood vessels are contractible and plastic, but as people age they become synthetic and less contractible in response to proinflammatory stimuli, diet, or other factors [33–35].

The significant pathways of negative and positive regulation of ERK1 and ERK2 cascade (PS = 0.3 and 4.3%), negative and positive regulation of dephosphorylation (PS = 5.3 and 5.6%), protein dephosphorylation (PS = 1.2%), actin binding (PS = 5.6%), response to camp (PS = 1.2%) may reveal some biological processes behind the regulation of vascular smooth cell and its subsequent effect on blood pressure regulation. Previous studies have detected significant roles of these pathways and other related pathways in regulation of vascular smooth muscle contraction [36, 37]. Brozovich et al. [38] provided a thorough description of these roles.

#### Regulation of smooth muscle contraction by epigenetic mechanism

Epigenetic mechanism refers to heritable changes of gene expression which are not related to the genome sequence [39]. These mechanisms may contribute in changing plasticity of vascular smooth muscle by either altering the accessibility of transcription factors at DNA regulatory regions or changing the genetic translations [40]. Our study identified histone methylation (PS = 4.1%) as a significant pathway to alter accessibility of transcription factors by changing chromatin packaging of the cells. Also, significant pathways of messenger RNA transcription (PS = 0.8%), basal transcription machinery binding (PS = 3.0%), transcription cofactor binding (PS = 1.7%) and damaged DNA binding (PS = 3.6%) may reveal more epigenetic mechanisms causing differential transcription of smooth muscle cell.

#### Cell-cell Signalling: WNT signaling

Non-canonical and canonical WNT pathways (PS = 5.4 and 5.5%) were found to be associated with trajectories of pulse pressure and multiple outcome of SBP and DBP. Massive literature has supported the association between WNT pathway and hypertension [41–43]. The study of these pathways has been motivated by heterogeneity of hypertensive patient population in response to antihypertensive medications. Patients with type 2 diabetes mellitus responded poorly to the treatment compared to others.

Many Genome Wide Association Studies (GWAS) suggest the association between hypertension and WNT3 that encodes a canonical WNT ligand and SOX proteins which interact with b-catenin and modulate the transcription of WNT-target genes [44–47]. In experiments, mice infused with angiotensin II have been diagnosed with activated b-catenin and proliferated vascular smooth muscle contraction. The other line of evidence supporting this relationship is the association of neurolocal regulation of blood pressure with interaction of insulin and WNT signaling [42].

#### DNA damage and genomic instability

The associations between age, development of cardiovascular diseases, and hypertension can be explained by pathways related to DNA damage and repair. This result is in agreement with our earlier observation that biological processes of intrinsic apoptotic signaling pathway in response to DNA damage (PS = 3.2%), nucleotide excision repair (PS = 4.2%), positive regulation of DNA repair (PS = 2.2%), and regulation of response to reactive oxygen species (ROS) (PS = 4.1%)are significantly associated with blood pressure trajectory over time. Below, there is a description of how these pathways collaborate to develop hypertension.

DNA is damaged by exposure to exogenous and endogenous agents, such as smoking and diabetes mellitus. Aging leads to prolonged exposure, accumulation of DNA damages, and elevated production of ROS at the molecular level. In order to preserve genomic stability under ROS-induced stress, multiple pathways to repair or respond to the presence of DNA damage are employed by the cell and their functions may overlap, compromise or exceed the capability to repair DNA. A defective DNA repair system leads to genomic instability and can accelerate development of vascular problems, such as increased blood pressure, increased vascular stiffness, and decreased vascular relaxation [48]. Also, multiple lines of evidence have suggested the direct or indirect effect of increased ROS on hypertension incidence, affecting blood vessels (contraction, relaxation and growth), heart, kidney [49] and nervous system functions [50]. This path of investigation can promote antioxidant therapies and production of drugs enhancing genomic integrity.

#### Nervous system development: pituitary development and ventral spinal cord development

Blood pressure changes can be related to nervous system development. In our study, we found pituitary development (PS = 0.4%) as a significant pathway affecting the pulse pressure and SBP&DBP trajectories. Endocrine hypertension, a special type of hypertension, is caused by the pituitary or adrenal gland producing too much or not enough of the hormones [51, 52]. Secretion of Antidiuretic hormone (vasopressin) by pituitary gland plays an important role in water retention in kidneys and controlling blood pressure. Furthermore, the imbalanced influence of the posterior and interior parts of pituitary gland is known to increase blood pressure [53].

The other significant nervous-system-related pathway in this study is spinal cord development (PS = 4.4%). Higher prevalence of hypertension among patients with spinal cord injury as a result of the interruption in the autonomic nervous pathways supports our finding. Reduction in autonomic cardiovascular control of hypertension explains this result [54].

#### Heart and blood vessel development

Our results are consistent with the significant influence of cardiac chamber development, coronary vasculature development (PS = 5.1%), embryonic heart tube development (PS = 4.3%), embryonic heart tube morphogenesis (PS = 2.8%), and blood vessel morphogenesis pathways (PS = 1.5%) on blood pressure trajectories.

The extra load on the thin wall chamber or tube caused by increased blood pressure is normalized by an increase in wall thickness and/or by a reduction in chamber/lumen diameter. More specifically, the left ventricle adopts its structure in response to imposed stress through remodelling or hypertrophy [55]. At the cellular level, cardiac gene expressions are altered in response to stress stimulus [56].

Overall, this study illustrated the application of LLCT on gene expression data measured on related and unrelated subjects. This was the first attempt to analyze gene sets when the blood pressure is repeatedly measured, and the data set is clustered by families. Analysis at the gene set level improves interpretability of findings. Incorporating repeated measurements of outcome over time enables us to investigate the temporal progression of phenotype over time. These studies provide the opportunity to investigate genomics under an important assumption: the effects of the genes contributing to the underlying phenotype are persistent over time. Also, the potential genetic and environmental covariates are better controlled via longitudinal study design. The family-based structure of data decreases heterogeneity leading to more precise investigations. The previous works in GAW19 never had these three features together. Although this study is unique in its kind, our findings have been shown to be mostly consistent with those of experimental or GWAS studies. However, we recognize that our study may not present the best set of pathways involved in blood pressure development because of the following limitations. The first limitation, albeit common among genomic studies, is that a single significant gene may lead to the significance of the whole pathway. Second, although we adjusted for anti-hypertensive intake and smoking status, there are many other uncontrolled covariates, such as diet, stress, and physical activity [57]. Lack of availability of informative covariates, such as behavioral recommendations that accompany medical prescriptions, has also been mentioned as a general limitation of GAW19 studies in the summary provided by Chiu et al. [58].

### Application 2: wound healing

A mouth wound may heal up to 3 times faster than a skin wound [59]. Researchers are interested to learn from the differential gene activations during skin and mouth wound healing to discover the genetic reasons underlying the speedy oral repairs. Understanding the genetic mechanisms involved in wound healing is critical for promoting a fast recovery through which many infections are prevented, many lives are saved, and many costs are decreased [60].

Several previous studies examined human and mice samples to find the genes or gene pathways which are differentially expressed in a mucosal cell compared to a skin cell after wounding. They mostly agreed that the transcriptome regulatory system leading the fast recovery of mucosal wounds are activated at unwounded state [59]. Consequently, as a mucosal cell needs to undergo a simpler molecular recovery process compared to a skin cell, a shorter healing time is expected.

Although previous findings contributed in better understanding of the speedy healing of mucosal tissue, we believe that there is still much more to investigate. Aside from the genes which are differentially expressed at the unwounded state, the genes with the similar expression levels before injury may exhibit differential expression pattern over time. In this application, we took advantage of LLCT to examine if the fast repair after oral injury is caused by the differential trajectories of gene expression, rather than their differential levels. We believe this analysis revealed more aspects of the undergoing transcriptome regulatory system.

This application study used the mice gene expression data collected from tongue and skin wounds of six- to eight-week-old female Balb/c mice at 6 h, 12 h, 1 day, 3 days, 5 days, 7 days and 10 days after the injury. Chen et al. [61] designed this experiment to explore the differences in gene expression in skin and tongue wound healing at different states of unwounded (referred as time 0), hemostasis (6 to 12 h), inflammation (24 h to 3 days), proliferation (5 days to 7 days) and remodeling (10 days). In this case study, we focused exploring the time 0 to 7 days as the remodeling state may take up to several months and an exact length time determination may not be possible [62]. At each time point, the total RNA from 3 skin samples and 3 tongue samples was hybridized to Affymetrix GeneChip Mouse Genome 430 v 2.0 chip. The closure time for tongue and skin wounds were 3 and 5 days, respectively. An interested reader may refer to Chen et al. [61] for more information regarding the experimental procedure.

We applied LLCT to analyze the association between the time trajectories of gene expression and the wound position in 247 KEGG pathways. The LLCT examined the changing patterns of gene expressions at different stages of hemostatic, inflammation and proliferation separately and in combination. LCT also was utilized to detect the pathways with non-differential gene-set expression level between tongue and skin wounds before the injury.

**Fig. 3:**
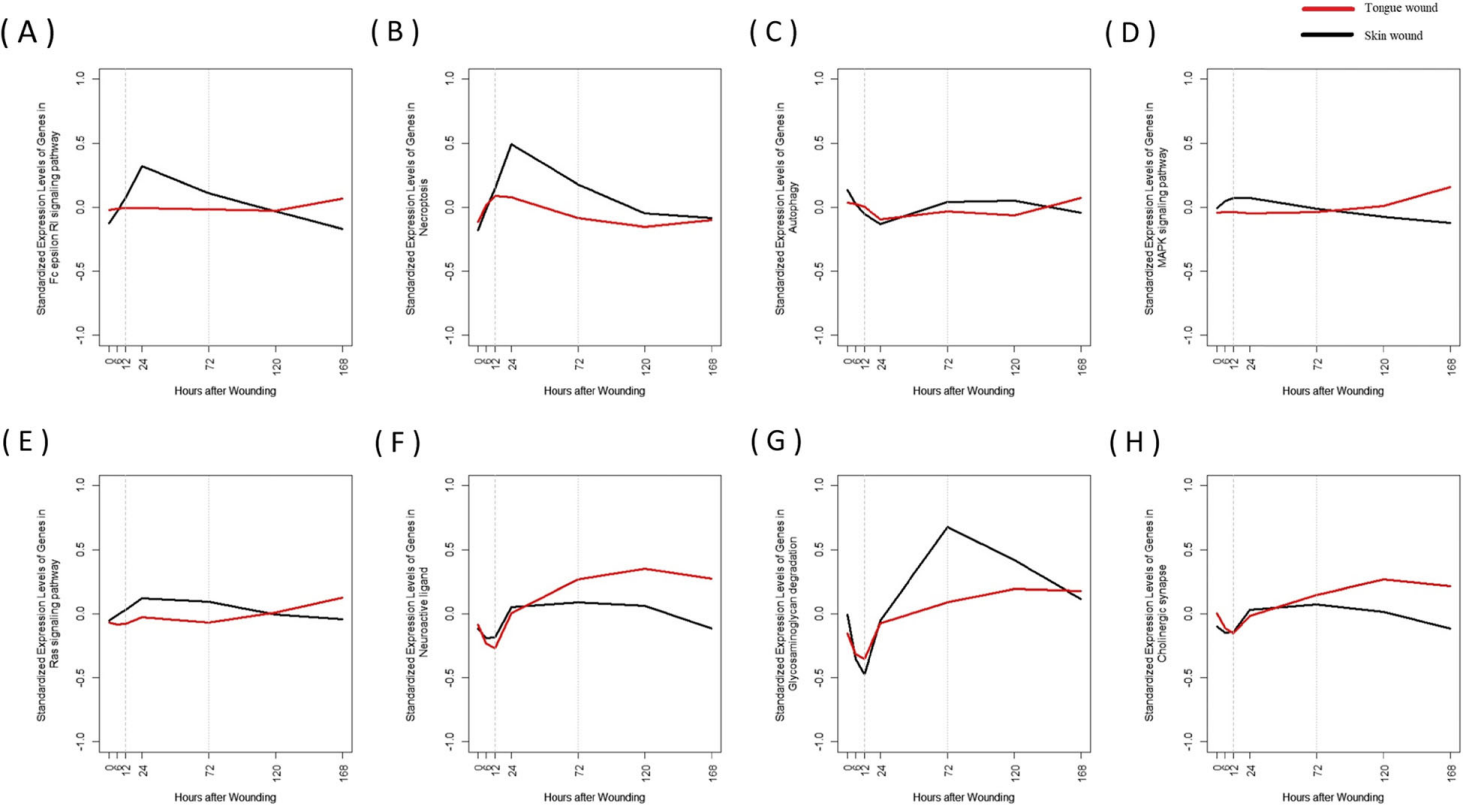
Time-course expression patterns of gene pathways. These gene sets were expressed non-differentially at unwounded state (time 0) but they are expressed differentially over all three stages of hemostasis (before 12 h after wounding), inflammatory (12–72 h after wounding) and proliferation (3–7 days after wounding)

Among 247 KEGG pathways selected, there were 95 pathways with differential expression pattern at hemostasis stage, 125 pathways with differential expression pattern at inflammation stage and 150 pathways with differential expression pattern at proliferation stage (Additional file 4). Table 3 lists the 38 gene pathways that are differentially expressed in all three stages of hemostatis, inflammation and proliferation for different organs of tongue and skin. The Additional file 4 provides the analysis results for all pathways. The eight pathways with the similar expression level at unwounded state can be identified using the last column of Table 3 and are plotted in Fig. 3.

**Table 3:** The list of KEGG pathways significantly expressed over three healing states of hemostasis, inflammation and proliferation for different wounds of skin and tongue, and their corresponding *p*-values calculated by LLCT method

	Geneset size	Hemostasis (< 12 h)	Inflammation (12–72 h)			Proliferation (3–7 days)		Differentially expressed at unwounded status?	Prioritization Score
		*p-*value	q-value*	*p*-value	q-value*	*p-*value	q-value*		
Metabolism									
Nucleotide metabolism									
Purine metabolism	789	< 0.01	< 0.01	0.01	0.03	< 0.01	< 0.01	Yes	9.0%
Glycan biosynthesis and metabolism									
Mucin type O-glycan biosynthesis	156	0.01	0.03	<0.01	<0.01	0.01	0.01	Yes	10.7%
Glycosaminoglycan degradation	108	0.03	0.09	0.01	0.02	<0.01	< 0.01	No	9.6%
Genetic Information Processing									
Transcription									
Spliceosome	984	<0.01	< 0.01	< 0.01	< 0.01	< 0.01	< 0.01	Yes	0.4%
Replication and repair									
DNA replication	237	<0.01	< 0.01	< 0.01	< 0.01	< 0.01	< 0.01	Yes	0.4%
Mismatch repair	135	<0.01	0.01	<0.01	<0.01	<0.01	<0.01	Yes	5.7%
Folding, sorting and degradation									
Protein processing in endoplasmic reticulum	1164	<0.01	<0.01	<0.01	<0.01	<0.01	<0.01	Yes	0.4%
Environmental Information Processing									
Signal transduction									
MAPK signaling pathway	2127	0.02	0.08	0.02	0.04	<0.01	<0.01	No	9.4%
Ras signaling pathway	1566	0.02	0.07	0.01	0.02	<0.01	<0.01	No	8.5%
Calcium signaling pathway	1206	0.01	0.03	<0.01	<0.01	<0.01	<0.01	Yes	3.1%
NF-kappa B signaling pathway	702	<0.01	< 0.01	< 0.01	< 0.01	< 0.01	< 0.01	Yes	0.4%
HIF-1 signaling pathway	804	<0.01	<0.01	<0.01	<0.01	<0.01	<0.01	Yes	0.4%
Hedgehog signaling pathway	378	<0.01	< 0.01	0.01	0.02	0.01	0.01	Yes	8.9%
JAK-STAT signaling pathway	972	<0.01	<0.01	<0.01	<0.01	<0.01	<0.01	Yes	0.4%
Signaling molecules and interaction									
Cytokine-cytokine receptor interaction	1308	<0.01	<0.01	<0.01	<0.01	<0.01	<0.01	Yes	0.4%
Neuroactive ligand-receptor interaction	1557	<0.01	0.01	<0.01	<0.01	<0.01	<0.01	No	2.2%
Cellular Processes									
Transport and catabolism									
Autophagy - animal	996	<0.01	0.01	0.02	0.04	<0.01	<0.01	No	10.8%
Lysosome	771	<0.01	<0.01	<0.01	<0.01	<0.01	<0.01	Yes	0.4%
Cell growth and death									
Apoptosis	951	0.03	0.09	<0.01	<0.01	<0.01	<0.01	Yes	5.1%
Necroptosis	849	0.01	0.03	<0.01	<0.01	<0.01	<0.01	No	3.5%
Cellular community - eukaryotes									
Adherens junction	609	0.02	0.06	0.01	0.01	0.01	0.01	Yes	12.6%
Organismal Systems									
Immune system									
Toll-like receptor signaling pathway	615	0.01	0.02	<0.01	<0.01	<0.01	<0.01	Yes	3.0%
RIG-I-like receptor signaling pathway	426	0.02	0.06	<0.01	<0.01	<0.01	<0.01	Yes	3.8%
Cytosolic DNA-sensing pathway	345	0.00	0.00	<0.01	<0.01	<0.01	<0.01	Yes	4.3%
Hematopoietic cell lineage	492	0.01	0.04	<0.01	<0.01	<0.01	<0.01	Yes	3.6%
Natural killer cell mediated cytotoxicity	720	0.02	0.06	<0.01	<0.01	<0.01	<0.01	Yes	6.7%
IL-17 signaling pathway	552	<0.01	0.01	<0.01	<0.01	<0.01	<0.01	Yes	2.2%
B cell receptor signaling pathway	540	0.03	0.09	<0.01	<0.01	<0.01	<0.01	Yes	5.0%
Fc epsilon RI signaling pathway	444	0.02	0.07	0.01	0.03	<0.01	<0.01	No	8.9%
Fc gamma R-mediated phagocytosis	696	0.04	0.11	<0.01	<0.01	<0.01	<0.01	Yes	5.4%
Leukocyte transendothelial migration	753	0.03	0.08	<0.01	0.01	<0.01	<0.01	Yes	8.4%
Circulatory system									
Adrenergic signaling in cardiomyocytes	1092	<0.01	0.01	0.01	0.01	<0.01	<0.01	Yes	10.5%
Development									
Axon guidance	1389	<0.01	<0.01	<0.01	<0.01	0.02	0.03	Yes	5.4%
Nervous system									
Glutamatergic synapse	777	0.02	0.07	<0.01	<0.01	0.01	0.01	Yes	8.6%
Cholinergic synapse	831	0.03	0.08	<0.01	<0.01	<0.01	<0.01	No	7.8%
Dopaminergic synapse	1041	<0.01	<0.01	<0.01	0.01	0.01	0.02	Yes	0.4%
Endocrine system									
Oxytocin signaling pathway	1125	<0.01	0.01	<0.01	<0.01	<0.01	<0.01	Yes	5.1%
AGE-RAGE signaling pathway in diabetic complications	717	0.01	0.03	<0.01	<0.01	<0.01	<0.01	Yes	3.1%

*P-values adjusted for False Discovery Rate (FDR)

Chen et al. [61] found KEGG pathways of Toll-like receptor signaling pathway and Jak-STAT signaling pathway to be early upregulated in skin wounds while the KEGG pathway of Cytokine-cytokine receptor interaction was observed to be upregulated in both organs with larger number of genes activating in skin cell. Consistently, we observed these three pathways underwent significantly distinct expression patterns in all three stages in association with the wound organ with a sharper increasing trend within the first 24 h after the skin injury. In agreement with Chen et al. [61], who reported a more refined gene responses after oral wounding, most of the pathways whose expression trajectories varied significantly by the related organ have shown relatively lower variation among oral samples.

There is extensive literature supporting the differential expression patterns of the 38 pathways we listed in Table 3, however this is beyond the scope and the length of this manuscript. Therefore, we are mainly discussing the pathways which were not differentially expressed at the initial state, but they underwent distinctive expression patterns after injury.

The prioritization scores indicated in the last column of Table 3 helps the reader evaluate the strength of LLCT in prioritizing biologically most relevant gene pathways.

#### Inflammations and immune systems

It is commonly reported that pathways related to inflammatory mediators, such as cytokine-cytokine receptor interaction and chemokine signaling pathways, and the immune system pathways were significantly over-expressed for skin cells [59]. The lower activation of immune processes during mucosal wound recovery prevents the chronic inflammation and decreases fibrosis and scarring [59, 63]. Our analysis showed that the skin and mucosal cells took differential expression patterns of inflammatory and immune factors in different states with a higher peak expression at 12 or 24 h after injury for skin wound and a subsequent steeper decline by Day 7. Although all the immune system pathways were upregulated before wounding, the basal pathway of Fc *ε* RI signaling expressed at the same level for different wound organs (Fig. 3a). According to a study conducted on skin-drived mast cells, the mast cells responses to Fc *ε* RI - mediated simulation through secretion of mediators like cytokines [64]. We consistently observed pattern similarities between cytokine-cytokine receptor interaction and Fc *ε* RI signaling pathway over time in different samples of skin and tongue. Therefore, the low level of cytokine expression in tongue wound could be regulated by the low expression of Fc *ε* RI signaling pathway.

#### Cellular death processes

Programmed cell death is required to maintain tissue hemostasis. Apoptosis and autophagy are known as a “programmed cell death”, which when inhibited, a new type of cell death, necroptosis, occurs [65]. In our study, the apoptosis, necroptosis and autophagy were differentially regulated over different types of skin and tongue samples. The higher upregulation of apoptosis in skin samples during the time course of wound healing compared to mucosal samples were also reported by Johnson et al. [66]. This study discussed that while apoptosis occurs predominantly through the intrinsic pathway in the healing mucosa, it occurs predominantly through the extrinsic pathway in skin samples [66]. In contrast to apoptosis pathway, the necroptosis and autophagy expressions at basal were statistically identical for both wound organs (Fig. 3b-c). As necroptosis expression is regulated by secretion of cytokines/chemokines, its post-injury fluctuations resemble that of cytokines/chemokines [67]. However, Autophagy regulates and is regulated by the inflammatory cytokine and chemokines, and therefore, its expression values did not reflect well the trajectories of cytokine and chemokine expressions [68].

#### Signal transduction

Having the same level of oral MAPK expression at unwounded state, skin cells responded immediately to the injury through overexpression of MAPK signaling pathway in the first day followed by a consistent decline afterwards. In contrast, the expression of this pathway in tongue cells remained approximately unchanged after wounding and started increasing in proliferation state (Fig. 3d). The level of MAPK signaling pathway expression for skin wound stayed lower than that of tongue wound in proliferation state. This pattern also resembles the observed pattern of RAS in this study (Fig. 3e). The RAS and MAPK pathways play a critical role in regulation of cell proliferation through a series of protein kinase cascades and their upregulation at proliferation phase in tongue wounds can help the wound rebuild [69].

Several evidences suggested the induction of tenascin-C, a fibroblast marker whose expression is necessary for accelerated wound healing [70], by several growth factors [71]. This association is regulated by neuroactive ligand-receptor interaction [72]. Therefore, the increasing expression pattern of neuroactive ligand-receptor interaction in the tongue cells, in contrast to the plateau and slightly decreasing pattern of that in the skin cells, could explain the rapid oral healing (Fig. 3f).

#### Metabolism

Glycosaminoglycan is a major component of connective tissue and play an important regulatory role in extracellular matrix production [73]. Our observation of distinct expression patterns of glycosaminoglycan in dermal and mucosal wounds over different wound healing stages accords the previous finding that suggested their distinctive features and quantities of glycosaminoglycan [73]. While expressed at the same level at unwounded state, the pathway of glycosaminoglycan degradation was overexpressed in the late hemostasis state and early inflammatory stage in tongue wound which helps coagulation, inhibits enzymes and cytotoxic mediators released from proinflammatory cells, and improves recruitment of inflammatory cells (Fig. 3g). The pathway of glycosaminoglycan degradation was greatly overregulated in late inflammatory state and over proliferation state of healing the skin wound. The excess expression of Heparin, a highly sulfated glycosaminoglycan, inhibits the activity of the FGF-7, which regulates keratinocytes migration and proliferation and therefore impedes the tissue development process [74].

#### Nervous system

Distinct response of Cholinergic Synapse pathway was observed in different wound organs. While Cholinergic Synapse pathway of skin wound reaches its highest expression level at day 1, the expression of this pathway in tongue tissue peaks at day 5 (Fig. 3h). In agreement, Anderson et al. [75] observed Acetylcholinesterase, an enzyme located at neuromuscular and central cholinergic synapse, to be highly expressed at day 1. They found the important role of Acetylcholinesterase in cell migration and fibroblast wound healing. Therefore, the increasing trend of theexpression of Cholinergic Synapse pathway in tongue sample by day 5 accounts for the speedy mucosal repair.

Our results suggested that tissue repair is a complex process involving multiple gene pathways. Our pattern analysis also suggested that the mucosal wounds required a less demanding procedure to repair, characterized by many invariant time-course expressions patterns, in comparison with the dermal wounds. We identified the important role of Fc *ε* RI signaling pathway in regulating the inflammatory mediators and the critical roles of neuroactive ligand-receptor interaction, cholinergic synapse and MAPK and RAS pathways in promoting the different healing phases. We also recognized the destructive effect of excess expression of glycosaminoglycan degradation in proliferation phase. All these findings improve our understanding of the molecular pathways and may contribute in devising new strategies for successful wounds treatment. We believe that a study of the time-course correlations between different gene pathways would also help advancing the grasp of the underlying transcriptome system.

## Discussion

The interest in temporal patterns of change in the patients’ conditions is becoming increasingly popular, as it aids in the explanation of complex biological systems. Longitudinal studies provide the possibility to study individual development of an outcome over time, and in doing so advance our understanding of disease progression or phenotype trajectory. Through longitudinal studies, the development of other variables can also be examined as determinants of the outcome trajectories. Therefore, incorporating longitudinal designs in genetic studies enable examination of genetic variants that affect phenotypes over time [58, 76]. Moreover, longitudinal studies are more reliable as the subjects are closely followed up with and the onset of the events is precisely observed [58]. Obviously, there is higher certainty behind the existence of an effect that is detected to be continuously significant over time in the presence of many uncontrolled or unmeasured time-dependent covariates than an effect that is observed only once. In other words, multiple measurements and significant trajectory over time provide more reliable evidence than what a single time point measurement and a cross-sectional effect can provide. Adding family structure to the study design can improve this reliability by detecting a significant genetic effect in a family rather than simply an individual.

The main purpose of the current study was to develop a statistical method for high-dimensional data able to analyze repeatedly-measured outcomes and covariates. This method offers many interesting flexibilities to the analysis. It allows adjusting for potentially time-dependent covariates. Since genetics and environment always interact to shape the phenotype, the result of genetics studies alone may be biased when environmental factors are not taken into account. A very common drawback of many available GSA methods is the lack of ability to accommodate between-gene correlation, which our proposed LLCT addresses. In addition, LLCT is a self-contained method proven to be powerful and computationally efficient compared to existing methods. Being a self-contained method, LLCT is also expected to result in high reproducibility, high power and high robustness to the sample heterogeneity, as applied on RNA-seq data [77]. This method can be applied to different classes of phenotypes, such as continuous, binary or categorical phenotype if an appropriate model is defined in the first stage. Furthermore, it is applicable to both unbalanced and incomplete data, which is important in longitudinal studies as some subjects are often lost to follow up. The evidence from the simulation study suggests higher power of LLCT in comparison to existing method, PAVR [7]. Aside from higher power of LLCT, there are two critical features that discriminate these two methods. First, LLCT is computationally far more efficient. Compared to LLCT, the run time is about 70 times longer for PAVR. For the same reason, we could not design a large simulation for evaluation of PAVR. Second, PAVR is unable to test the interaction of time and covariate over time and it only tests the covariate effect. The interaction of time and covariate indicates if the covariate’s effect varies over time; which is known to be the most critical parameter of longitudinal analysis. Without considering this parameter, the longitudinal study resembles a cross-sectional study that takes advantage of multiple measurements for gaining higher accuracy of measurements. Our simulation study also showed that the power, and therefore the required sample size, is dependent on the gene set size and the within-gene-set correlation and it is independent on the number of repeated measurements and within-subjects correlation. Moreover, Significance of a set exhibiting lower between-genes correlations can be achieved with a smaller sample size..

Despite the strengths mentioned above, there are a few limitations for this method that need to be considered. Our method for dealing with longitudinal phenotype is unable to adjust for time-independent covariates. Including time-independent covariates in the second step of the method may result in misleading findings. As a self-contained method, LLCT would identify a set as significant even if a small number of genes, or even if one single gene is associated with the phenotype. One way to address this limitation is to consider reducing the significant sets to their core members. In time-course microarray data analysis, this method can identify the gene sets which are differentially expressed over time in association with a set of covariates. However, our method is unable to distinguish the individual covariates responsible for this difference, unless we include one covariate at a time. Resembling the mixed effect models, LLCT assumes the expressions of the genes within a geneset can be characterized by the same type of model. If the study includes the genes with differential patterns of expression which could not be characterized by a single type of model, spline models should be used. The researcher may also consider analysis of the expression trajectory in the time subperiods.

LLCT was applied to GAW19 data. As noted earlier, GAW19 has been analyzed before. However, significant differences across various methods used prevented a meaningful comparison of the results. There are four pedigree-based GAW19 studies exploring the association between phenotype and gene expressions via different methods: linear mixed models, nonparametric weighted U statistics, structural equation modeling, Bayesian unified frameworks, and multiple regression. However, their results cannot be compared with ours because of the following differences in the analytical approaches: (1) They incorporated the information of rare variants into their analysis while this study examined the transcriptome variations only; (2) In contrast to our GSA approach, they did not include the priori information of gene pathways; (3) They did not take the longitudinal pattern of the phenotype into the account. There are seven GAW19 pathway-based analysis, three of which explored gene expression data [78]. There are three GAW19 studies with longitudinal analytical approaches, all of them examining genetic variants [58]. The longitudinal studies used GEE, latent class growth modeling (LCGM), LMM, and variance components (VC) in their analysis. Among all these studies, the study of Ziyatdinov et al., which is a gene ontology pathway and family-based enrichment analysis of gene-expression data using the mixed effect models, came closest to the current study, but it is unpublished at the time of submission of this work. GAW19 studies acknowledged higher power of longitudinal methods in detecting genetic effects, decreased trait heterogeneity and smaller standard error of effect estimates [58]. Also, they identified unique genetic-related trajectories of disease progression missed by the previous studies.

We demonstrated the applicability of LLCT on analysis of time-course microarray data in the second application. Contrary to the first application, LLCT dealt with a binary phenotype collected from mice samples in this application. The data was previously analyzed using ANOVA for comparing the expression level at each post-wounding time with that of unwounded state. In order to gain some understanding of the underlying expression dynamics, the temporal trajectories of the genes which were differentially expressed were K-means clustered [61]. The inherent assumptions of this clustering method limit the ability to describe the differential dynamic behaviour of the genes and, also, do not provide a test of time-course diversities.

Although we found many phenotype-relevant gene sets in our application, that are supported by previous studies, a future study comparing the sensitivity and specificity of the LLCT applied to a large number of longitudinal studies is needed.

## Conclusion

The proposed LLCT method can be used for analysis of complex genetic studies and may result in better reproducibility across studies. LLCT can be applied to a wide range of longitudinal genomics, transcriptomics, proteomics, metabolomics and microbiota data. A very important application of LLCT is to link omics over time - an approach that has been emphasized by recent studies for gaining better understanding of complex biological process. Linkage of omics over time requires a method that can handle large scale outcomes and predictors datasets, simultaneously, which cannot be accommodated by most methods. Our method has strong potential to contribute to the progression of genetic science.

## Methods

### Longitudinal linear combination test (LLCT)

We propose a two-step method to analyze multiple longitudinal phenotypes when there is high dimensionality in either the response or explanatory variables. In the first step, within-subject variation is analyzed. The changing trend of outcomes over time is estimated using an appropriate model for the structure and type of the data. In the second step, LCT [79] is applied to analyze the between-subject variation. In this step, LCT is employed to examine if the maximum possible correlation between a linear combination of the time trends and a linear combination of the predictors given by the gene expressions is statistically significant. We generalized our method to accommodate data generated by two complex study designs: time-course microarray studies and family-based studies. A time-course study measures gene expression repeatedly over time and is designed to find the correlation between time trajectory of gene-expressions and covariates. A family-based design collects the information from family members and examines the association between longitudinal phenotypes and gene expressions, while taking care of the correlation between subjects within each family.

We borrowed the main idea of this method from mixed effect modelling wherein the variation in the longitudinal phenotype is modelled taking two steps: first step, the within-subject variation is modelled; in the second step, the between-subject variation is modeled using the coefficients estimated in the first step [80]. Roughly the same strategy is also practiced by Conesa et al. [8] in their microarray significant profiles (maSigPro) method.

The proposed method is designed to model continuous outcome variables. However, this method can be generalized to work with other type of data, such as binary or categorical response variable, using an appropriate link function in the first step. This method is self-contained, designed to accommodate the correlations between genes in the gene sets, works well in the presence of missing data at random and is efficient to work with high dimensional data. It can also adjust for time-variant covariates. Next, we describe the two steps of the method, followed by two generalizations.

#### *Analysis of Within-Subject Variation (Step 1)*

Consider a microarray study on I subjects where longitudinal phenotypes of size M is measured for *n*_*i*_ times for the *i* th subject, *i* = 1, …, *I*. Let *Y*_*mij*_ be the *j th* measurement (*j* = 1, …, *n*_*i*_) of the *m* th phenotype (*m* = 1, …, *M*) of the *i* th subject that happened at time *t*_*ij*_ and let ${Y}_{mi}={\left({Y}_{mi1},\dots, {Y}_{mi{n}_i}\right)}^T$ be the vector of *n*_*i*_ measurements of the *m* th phenotype for the *i* th subject ($\sum \limits_{i=1}^I{n}_i={n}_{.}\Big)$ and *Y*_*i*_ = (*Y*_1*i*_, …, *Y*_*Mi*_) be the matrix of phenotype measurements of the *i* th subject. We also consider that the study measured the expressions of a predefined set of *P* genes for the *i* th subject, *G*_*i*_ = (*G*_*i*1_, …, *G*_*iP*_)^*T*^, *i* = 1, …, *I*; and we define the vector of the expressions of gene *p* for *I* subjects as *G*_*p*_ = (*G*_1*p*_, …, *G*_*Ip*_)^*T*^, *p* = 1, …, *P*. We are interested to test if there is a significant linear relationship between the gene set *G* and the longitudinal phenotype *Y*. The null hypothesis is that the changes in Y over time are not dependent on the expressions of the genes in the predefined gene set G.

In order to analyze within-subject correlation, we define the regression equation in matrix notation as below:

1 $$ {Y}_{mi}={Z}_i{\beta}_{mi}+{W}_i{\gamma}_{mi}+{\varepsilon}_{mi} $$

In this equation, *Z*_*i*_ is (*n*_*i*_ × *Q*) matrix of *Q* potential time variables and it usually includes ${t}_i=\left({t}_{i1},\dots .,{t}_{i{n}_i}\right)$ and different polynomial functions of *t*_*i*_ (e.g. ${t}_i^2,{t}_i^3$) if required. *W*_*i*_ is (*n*_*i*_ × *Q* ′ ) matrix of *Q*′ potential time-variant covariates, and *γ*_*mi*_(*Q* ′  × 1) represents their corresponding coefficients. Also, *β*_*mi*_ denotes a (*Q* × 1) vector of coefficients for each specific phenotype (*m*) with elements of *β*_*mqi*_. We define *β*_*i*_ a (*Q* × *M*) matrix of regression coefficients generated by column-wise binding of *β*_*mi*_ s: *β*_*i*_ = [*β*_1*i*_|*β*_2*i*_|…| *β*_*Mi*_].

#### *Analysis of Between-Subject Variation (Step 2)*

In our method, we used Linear Combination Test (LCT) [79] to detect significant gene sets associated with different trajectories of longitudinal phenotypes. We reason that a lack of gene set related variability in the subject-specific regression coefficient estimated in the first step, leads to no relationship between the gene set expressions and the changing trend of M longitudinal phenotypes. In other words, there is no linear combination of the columns of $\beta ={\left[{\beta}_1^T|\dots |{\beta}_I^T\right]}^T$ associated to any linear combination of gene set expression measurements. The null hypothesis is that there is no association between any of the linear combination of *G*_1_, …, *G*_*P*_ with any linear combination of columns of *β*.

Let G be a ((*I* × *Q*) × (*P*)) matrix obtained by vertically merging the vectors of the gene expressions, *G*_*p*_ s, duplicating the rows for Q times. Then, let

2 $$ Z\left(G,\mathrm{A}\right)={\left[\begin{array}{c}\begin{array}{ccc}{G}_{11}& \dots & {G}_{1P}\\ {}\vdots & \ddots & \vdots \\ {}{G}_{11}& \dots & {G}_{1P}\end{array}\\ {}\begin{array}{ccc}{G}_{21}& \dots & {G}_{2P}\\ {}\vdots & \ddots & \vdots \\ {}{G}_{21}& \dots & {G}_{2P}\end{array}\\ {}\begin{array}{ccc}\vdots & \vdots & \vdots \\ {}{G}_{I1}& \dots & {G}_{IP}\\ {}{G}_{I1}& \dots & {G}_{IP}\end{array}\end{array}\right]}_{(I.Q)\times (P)}\times {\left[\begin{array}{c}\begin{array}{c}{\alpha}_1\\ {}{\alpha}_2\end{array}\\ {}\vdots \\ {}{\alpha}_P\end{array}\right]}_{(P)\times 1} $$

be a linear combination of the columns of matrix G, and,

3 $$ Z\left(\mathrm{B},\Gamma \right)={\beta}_{(I.Q)\times M}\times {\left[\begin{array}{c}\begin{array}{c}{\gamma}_1\\ {}{\gamma}_2\end{array}\\ {}\vdots \\ {}{\gamma}_M\end{array}\right]}_{(M)\times 1} $$

a linear combination of columns of *β*. The null hypothesis can be written as an optimization problem, more precisely, identifying A and B to maximize the correlation of *Z*(*G*, Α) and (Β, Γ), and then test if this maximum correlation is significant or not.

Let Σ_G, G_ =  *cov* (G, G) be the covariance matrix of G; and similarly, let Σ_Β, Β_ =  *cov* (Β, Β) be the covariance matrix of Β and Σ_G, Β_ =  *cov* (G, Β) be the covariance matrix between G and Β. This leads to the proposed test statistic:

4 $$ {T}^2={\mathit{\max}}_{A,B}{\left|\rho \left(Z\left(\mathrm{G},\mathrm{A}\right),Z\left(\mathrm{B},\Gamma \right)\right)\right|}^2={\mathit{\max}}_{A,B}\frac{{\left({\mathrm{A}}^T{\Sigma}_{\mathrm{B},\mathrm{G}}\Gamma \right)}^2}{{\mathrm{A}}^T{\Sigma}_{\mathrm{G},\mathrm{G}}\mathrm{A}.{\Gamma}^T{\Sigma}_{\mathrm{B},\mathrm{B}}\Gamma} $$

The problem of singularity of Σ_Β, Β_ and Σ_G, G_ emerges when the dimensions of Β or G are large. This is very likely to happen as we usually measure the expressions of a large number of gene sets. A possible remedy for this problem is to utilize the shrinkage method [81]. Therefore, we need to replace the covariance matrices with their shrinkage versions, ${\Sigma}_{\mathrm{B},\mathrm{B}}^{\ast }$ and ${\Sigma}_{\mathrm{G},\mathrm{G}}^{\ast }$. *T*^2∗^, the shrinkage version of *T*^2^, is defined as below:

5 $$ {T}^{2\ast }={\mathit{\max}}_{A,B}\frac{{\left({\mathrm{A}}^T{\Sigma}_{\mathrm{B},\mathrm{G}}\Gamma \right)}^2}{{\mathrm{A}}^T\ {\Sigma}_{\mathrm{G},\mathrm{G}}^{\ast}\mathrm{A}.{\Gamma}^T{\Sigma}_{\mathrm{B},\mathrm{B}}^{\ast}\Gamma} $$

We use the permutation method to calculate the *p*-value corresponding to this statistic. When the permutation method is employed, it would be computationally inefficient to maximize the right-hand side of the equation above. The remedy could be using two groups of normalized orthogonal bases, instead of using the original observation vectors G and Β. We decomposed the two shrinkage covariance matrices using eigenvalues (${\Sigma}_{\mathrm{G},\mathrm{G}}^{\ast }=\Psi {D}_{\mathrm{G}}{\Psi}^T$ and ${\Sigma}_{\mathrm{B},\mathrm{B}}^{\ast }=\Omega {D}_{\mathrm{B}}{\Omega}^T$) to obtain two groups of orthogonal basis vectors $\overset{\sim }{\mathrm{G}}$ and $\overset{\sim }{\mathrm{B}}$. Thus, the test statistic becomes:

6 $$ {T}^{2\ast }=\underset{\eta, \theta }{\max}\frac{{\left({\eta}^T{\Sigma}_{\overset{\sim }{\mathrm{G}},\overset{\sim }{\mathrm{B}}}\theta \right)}^2}{{\left\Vert \eta \right\Vert}_2^2.{\left\Vert \theta \right\Vert}_2^2} $$

where $\eta ={D}_{\mathrm{G}}^{1/2}{\Psi}^T\mathrm{A}$ and $\theta ={D}_{\mathrm{B}}^{1/2}{\Omega}^T\Gamma$. Optimizing this expression will be straightforward if we first optimize *η* given *θ* and then optimizing *θ* at the next step. The value of *T*^2∗^ is equal to the largest eigenvalue of ${\Sigma}_{\overset{\sim }{\mathrm{G}},\overset{\sim }{\mathrm{B}}}^T{\Sigma}_{\overset{\sim }{\mathrm{G}},\overset{\sim }{\mathrm{B}\ }}$ (or ${\Sigma}_{\overset{\sim }{\mathrm{B}},\overset{\sim }{\mathrm{G}}}^T{\Sigma}_{\overset{\sim }{\mathrm{B}},\overset{\sim }{\mathrm{G}}}$).

The sample permutation method is employed to calculate *p*-values. The sample permutation changes neither the correlation structure within gene sets nor the correlation structure within phenotype. This feature brings a considerable computational advantage to the analysis because there is no need to repeat eigenvalue decomposition for each permuted version of the dataset.

The LLCT method can also be generalized to analyze (1) family-based data in which the subjects are nested within families and expected to share many similarities; and, (2) time-course microarray data in which the gene expressions are repeatedly measured over time. These two generalizations are expanded in Additional file 1.

### Simulation study design

A simulation study was designed to evaluate the performance of LLCT method and compare its performance with PAVR proposed by Adewale et al. [7]. Several simulations were generated with varying number of subjects, gene set sizes, number of repeated measurements, within-gene set correlation, within-subject correlation and gene set effect sizes. The number of subjects and gene set size changed from 30,50 to 100.

For each gene set, gene expressions are simulated from *MVN*(Μ_*G*_, Σ_*G*_) where Μ_*G*_ is the mean vector of gene expressions, taken from a truncated exponential distribution with *λ* = 0.7. Σ_*G*_ is the variance-covariance matrix of genes within a gene set. The variances of the genes were set at ${\sigma}_G^2=0.5$ and the correlations between genes were set at *ρ*_*G*=_.0.1, 0.5 or 0.7. The effect of within-gene set correlation on the performance of the method was evaluated.

For each gene set, the longitudinal data was simulated based on the following model:

7 $$ {y}_{ij}={\mathrm{B}}_1\times {GS}_i+{\mathrm{B}}_2\times {t}_i+{\mathrm{B}}_3\times {GS}_i\times {t}_i+{b}_{0i}+{b}_{1i}\times {t}_i+{\varepsilon}_{ij} $$

Where *y*_*ij*_ denotes the *j* th observation of the *i* th subject; *GS*_*i*_ is the vector of gene expression measurements for *i* th subject; Β_1_ is the vector of fixed effects of the genes on the longitudinal phenotype, with values of 0.05,0.1 and 0.2 for all the subjects; *t*_*i*_ is the measurement time vector of the *i* th subject varying from one subject to another. The length of *t*_*i*_ is set at 3,4 and 5 in different simulations, but the time points of measurement were uniformly distributed between 1 and 10. Β_2_ is the vector of fixed effect of time on phenotype, set at 0.3 for all the subjects. Β_3_ is the vector of fixed effects of interactions of gene expressions at time and was set at 0.25,0.05 and 0.1 for all subjects in different simulations. *b*_0*i*_~*N*(0, 1) and *b*_1*i*_~*N*(0, 2) are the random constant and the random effect of subject *i*, respectively and are assumed to be independent among subjects. *ε*_*ij*_ is the error term defining the variation of the *j* th observation of subject *i*. *ε*_*ij*_ is assumed to be correlated within subjects. In this simulation, the correlation structure of *ε*_*ij*_ is autoregressive and we assumed: $cor\left({\varepsilon}_k,{\varepsilon}_l\right)={\rho}_{\varepsilon}^{k-l}$ where *ρ*_*ε*_ = 0.2,0.5 *or* 0.7.

For LLCT simulation, we simulated 1000 gene sets in each run and each *p*-value was calculated based on 1000 permutations. In simulations of PAVR, the results are based on 50 permutations.

## Supplementary information

**Additional file 1.** The methodology descriptions of the generalizations of LLCT methods for analysis of: (1) Family-based data (2) Time-course microarray data.

**Additional file 2.** Results of LLCT examining the differential expressions of different gene sets in association with various measures of blood pressure for UNRELATED subjects in GAW19 dataset.

**Additional file 3.** Results of LLCT examining the differential expressions of different gene sets in association with various measures of blood pressure for RELATED subjects in GAW19 dataset.

**Additional file 4.** Results of LLCT analysis examining the differential time-course expressions of different gene sets in association with the position of the wound (skin or tongue).

## Data Availability

The GAW19 dataset supporting the first application of this article is available to any researcher who requests them from Genetic Analysis Workshops (http://www.gaworkshop.org). The dataset supporting the wound healing application is accessible in the GEO data repository (http://www.ncbi.nlm.nih.gov/geo/) under accession number GSE23006. LLCT package for conducting LLCT analyses was produced by R3.5.3 and is available at https://github.com/its-likeli-jeff/LLCT.

## Abbreviations

DBP: Diastolic blood pressure
GAW: Genetic Analysis Workshop
GEE: Generalized Estimating Equations
GEO: Gene Expression Omnibus
GSA: Gene Set Analysis
GWAS: Genome Wide Association Studies
HTN: hypertension status
IGA: Individual Gene Analysis
KEGG: Keyoto Encyclopedia of Genes and Genomes
LCGM: Latent class growth modeling
LLCT: Longitudinal linear combination test
LMM: Linear Mixed Models
PAVR: Pathway Analysis via Regression method
PP: Pulse pressure
SAFHS: San Antonio Family Heart Study
SBP: Systolic blood pressure
TCGA: The Cancer Genome Atlas
VC: Variance components
VIF: Variance Inflation Factor
