# Supplementary material for: Longitudinal linear combination test for gene set analysis
Source: BMC Bioinformatics. 2019 Dec 10;20:650. doi: 10.1186/s12859-019-3221-7 (PMC6902471; doi:10.1186/s12859-019-3221-7)
Supplement: Supplementary file 1 — Additional file 1. The methodology descriptions of the generalizations of LLCT methods for analysis of: (1) Family-based data (2) Time-course microarray data. [file 12859_2019_3221_MOESM1_ESM.docx]

**Generalization 1: LLCT for Family-Based Data**

Consider a microarray study on $I$ subjects in which $M$ longitudinal phenotype is measured for $F$ families. Also consider that the number of subjects in family $f$ is $I_{f}$ and the number of repeated measurements for subject $i$ in family $f$ is $n_{fi}$ so that $\sum_{f=1}^{F} \sum_{i=1}^{I_{f}} n_{fi}=n_{..}$ is the total number of observations in the study. Let $Y_{mfij}$ be the $j th$ measurement ($j=1,\ldots,n_{fi}$) of the $m$*th* phenotype $(m=1,\ldots,M)$ recorded for the $i$th subject who belongs to family $f$ and measured at time $t_{fij}$. Also consider that the study measured the expressions of a predefined set of $P$ genes for the $i$th subject of family $f$, and we define the vector of the expressions of the $p$th gene as $G_{p}=\left( G_{11p}, \ldots{,G}_{1I_{1}p}, \ldots,G_{F1p}, \ldots,G_{FI_{F}p} \right)^{T},p=1,\ldots,P$. We are interested to test if there is a significant linear relationship between the gene set $G_{fi}$and the longitudinal trajectory of all longitudinal phenotypes$Y_{mfij}$. The null hypothesis is that the changes in $Y$s over time are not dependent to the expressions of the genes in the gene set of interest.

In the first step of our generalized method, we model the within-family variation for phenotypes separately using mixed effect model. So, in a matrix format, we define:

| $Y_{m}=X\beta_{m}+W\gamma_{m}+Zb_{m}+\varepsilon_{m}$ | (1) |
| --- | --- |

In this equation,$Y_{m}$ denotes a $(n_{..}\times1)$ vector of the $m$th phenotype measurements for all families and all subjects, $X$($n_{..}\times R)$ denotes a matrix of time variables (usually including a vector of $t$ and different functions, such as $t^{2},t^{3})$, and $Z$ is ($n_{..}\times(F.Q))$ matrix of the potential covariates for random effects with the format of $Z=\left( \begin{matrix} Z_{1} & 0 & 0 \\ 0 & \ddots& 0 \\ 0 & 0 & Z_{F} \end{matrix} \right)$ where$Z_{f}\in\mathbb{R}^{I_{f}\times Q}$. Also, $\beta_{m}$ is a $(R\times1)$ vector of family fixed regression coefficients for time variables corresponding to $m$th phenotype. $W$($n_{..}\times R')$ represents a matrix of potentially time-dependent and time-independent (but subject-variant) covariates for which the estimations are adjusted. The vector of coefficients is denoted by $\gamma_{m}(R'\times1)$. The $((F.Q)\times1)$ vector of random effects is defined as $b_{m}$ and varies by families. The ($n_{..}\times1)$ vector of residuals is $\varepsilon_{m}$ and we have $\left( \begin{matrix} b_{m} \\ \varepsilon_{m} \end{matrix} \right)\sim N_{Q+n_{..}}\left( \left( \begin{matrix} 0 \\ 0 \end{matrix} \right),\left( \begin{matrix} \Psi& 0 \\ 0 & \Omega\end{matrix} \right) \right)$ where $\Omega=\left( \begin{matrix} \Sigma_{n_{f1}} & 0 & 0 \\ 0 & \ddots& 0 \\ 0 & 0 & \Sigma_{n_{fI_{f}}} \end{matrix} \right)$ and $\Psi$is the covariance matrix of random effects that must be estimated.

Gene expressions are also correlated within families. Therefore, we will take an additional step to accommodate the within-family association of gene expressions using random intercept model:

| $G_{p}=\Xi_{p}+\xi_{p}+\varepsilon_{p}^{*}$ | (2) |
| --- | --- |

In this model, $G_{p}$is $\left( I\times1 \right)$vector of gene expressions of $p$th gene for all families, $\varepsilon_{p}^{*}$is $\left( I\times1 \right)$vector of residuals, $\Xi_{p}$is $\left( I\times1 \right)$vector of fixed intercept, a constant for all families, $\xi_{p}$is $(I\times1)$ vector of random intercepts and its elements vary for each family.

In the second step of our method, we will use LCT for multiple phenotypes to examine the between-family variations. If there is no gene set related variability in the family-specific regression coefficient, there will be no relationship between the gene set expressions and changing trend of M longitudinal phenotypes. In other words, there is no linear combination of family-specific phenotype trajectories $b={[b_{1}^{T}|\ldots|b_{M}^{T}]}^{T}$ associated to any linear combination of family-specific gene set expression measurements $\xi=[\xi_{1}|\ldots|\xi_{P}]$. The null hypothesis, here, is defined to be no association between any of the linear combination of $\xi_{1}, \ldots, \xi_{P}$ with any linear combination of columns of b.

Let G be a $((F.Q)\times P)$ matrix which is created by vertically merging the vectors of $\xi_{p}$s and duplicating each row for Q times. Then, let

| $Z\left( G,A \right)=\left[ \begin{matrix} \begin{matrix} \xi_{11} & \ldots& \xi_{1P} \\ \vdots& \ddots& \vdots\\ \xi_{11} & \ldots& \xi_{1P} \end{matrix} \\ \begin{matrix} \xi_{21} & \ldots& \xi_{2P} \\ \vdots& \ddots& \vdots\\ \xi_{21} & \ldots& \xi_{2P} \end{matrix} \\ \begin{matrix} \vdots& \vdots& \vdots\\ \xi_{(F.Q)1} & \cdots& \xi_{(F.Q)P} \\ \xi_{(F.Q)1} & \ldots& \xi_{(F.Q)P} \end{matrix} \end{matrix} \right]_{(F.Q)\times P}\times\left[ \begin{matrix} \begin{matrix} \alpha_{1} \\ \alpha_{2} \end{matrix} \\ \vdots\\ \alpha_{P} \end{matrix} \right]_{(P)\times1}$ | (3) |
| --- | --- |

be a linear combination of the columns of matrix G, and,

| $Z\left( B, \Gamma\right)=b_{(F.Q)\times(M)}\times\left[ \begin{matrix} \begin{matrix} \gamma_{1} \\ \gamma_{2} \end{matrix} \\ \vdots\\ \gamma_{M} \end{matrix} \right]_{(M)\times1}$ | (4) |
| --- | --- |

a linear combination of the columns of $b$s. As before, the null hypothesis can be written as an optimization problem, more precisely, identifying A and B to maximize the correlation of $Z\left( G,A \right)$ and $\left( B, \Gamma\right)$ , and then test if this maximum correlation is significant or not.

**Generalization 2: Time-Course Microarray Data Analysis**

We considered this application as a special case of the general framework of analyzing multiple longitudinal data. In this case, the longitudinal gene expressions measurements of a specific gene set are treated as multiple longitudinal phenotypes. Consider a microarray study on $I$ subjects where the gene expressions of a specific gene set are measured for $n_{i}$ times for the $i$th subject, $i=1,\ldots,I$. Let $G_{pij}$ be the $j th$ measurement ($j=1,\ldots,n_{i}$) of the $p$th gene expression in the gene set $(p=1,\ldots,P)$ for the $i$th subject that happened at time $t_{ij}$ and let $G_{pi}={(G_{pi1}, \ldots, G_{pin_{i}})}^{T}$ be the vector of $n_{i}$ expression measurements of the $p$th gene for the $i$th subject ($\sum_{i=1}^{I} n_{i}=n_{.})$ and $G_{i}=\left( G_{1i},\ldots,G_{Pi} \right)$ the ($n_{i}\times P)$ matrix of phenotype measurements of the $i$th subject. We are interested to test if there is a significant linear relationship between the specific gene set $G$ and a set of time-invariant covariates $C$. The null hypothesis is that the changes in predefined gene set G over time are not dependent on the covariates C. In other words, the genes in a specific gene set are not differentially expressed over time in response to the changes of covariates.

In this application we only modify the first step of our proposed LCT method to analyze within-subject variations. The second step, where LCT is employed to analyze between-subject variations, remains unchanged.



Consider the following model:

| $G_{ip}=Z_{i}\beta_{ip}+W_{i}\gamma_{ip}+\varepsilon_{ip}$ | (5) |
| --- | --- |

where $Z_{i}$ is ($n_{i}\times Q)$ matrix of the time variables and it usually includes $t_{i}=\left( t_{i1},\ldots.,t_{in_{i}} \right)$ and different functions of $t_{i}$ (e.g. $t_{i}^{2}, t_{i}^{3}$ ). $W_{i}$ is the matrix of potential time-dependent covariates for which we would like the estimations to be adjusted, with corresponding ($Q'\times1)$ vector of coefficients of $\gamma_{ip}$. Also, $\beta_{ip}$ denotes a ($Q\times1)$ vector of coefficients of time variables corresponding to $p$th gene, with components denoted as $\beta_{iqp}$. We define $\beta_{i}$a ($Q\times P)$ matrix of regression coefficients generated by column-wise binding of $\beta_{ip}$s.

In the second step, we use LCT to examine the relationship between the covariates and the changing trend of the longitudinal gene set expressions. If there is no covariate related variability in the gene set-specific regression coefficient, there will be no relationship between the covariates and the changing trend of the specific gene set expressions. In other words, there is no linear combination of the columns of $\beta={[\beta_{1}^{T}|\ldots|\beta_{I}^{T}]}^{T}$ associated to any linear combination of covariates measurements. Therefore, if $C_{i}=(C_{i1}, \ldots, C_{iU})$ is the vector of time-invariant covariates for the $i$th subject and $C_{u}=\left( C_{1u}, \ldots, C_{Iu} \right), u=1,\ldots,U$, the null hypothesis can be formulated as:

“There is no association between any of the linear combination of $C_{1},\ldots, C_{U}$ with any linear combination of columns of $\beta$.”

Let $C^{*}$ be a $((I.Q)\times\left( U \right))$ matrix obtained by duplicating the covariates measurements of subject $i$for $Q$ times. Then, let

| $Z\left( C^{*},A \right)=\left[ \begin{matrix} \begin{matrix} C_{11} & C_{12} \\ \vdots& \vdots\end{matrix} & \begin{matrix} \cdots& C_{1U} \\ \ddots& \vdots\end{matrix} \\ \begin{matrix} C_{11} & C_{12} \\ \begin{matrix} C_{21} \\ \vdots\\ \begin{matrix} C_{21} \\ \vdots\\ \begin{matrix} C_{I1} \\ C_{I1} \end{matrix} \end{matrix} \end{matrix} & \begin{matrix} C_{22} \\ \vdots\\ \begin{matrix} C_{22} \\ \vdots\\ \begin{matrix} C_{I2} \\ C_{I2} \end{matrix} \end{matrix} \end{matrix} \end{matrix} & \begin{matrix} \cdots& C_{1U} \\ \begin{matrix} \cdots\\ \ddots\\ \begin{matrix} \cdots\\ \ddots\\ \begin{matrix} \cdots\\ \cdots\end{matrix} \end{matrix} \end{matrix} & \begin{matrix} C_{2U} \\ \vdots\\ \begin{matrix} C_{2U} \\ \vdots\\ \begin{matrix} C_{IU} \\ C_{IU} \end{matrix} \end{matrix} \end{matrix} \end{matrix} \end{matrix} \right]_{(I.Q)\times(U)}\times\left[ \begin{matrix} \begin{matrix} \alpha_{1} \\ \alpha_{2} \end{matrix} \\ \vdots\\ \alpha_{U} \end{matrix} \right]_{U\times1}$ | (6) |
| --- | --- |

be a linear combination of the columns of matrix $C^{*},$and,

| $Z\left( B, \Gamma\right)=\beta_{(I.Q)\times P}\times\left[ \begin{matrix} \begin{matrix} \gamma_{1} \\ \gamma_{2} \end{matrix} \\ \vdots\\ \gamma_{P} \end{matrix} \right]_{(P)\times1}$ | (7) |
| --- | --- |

be a linear combination of columns of $\beta$. As before, LCT can be used to find out the maximum correlation between Z(C*, A) and Z(B,Γ), and test its significance using permutation tests
