# Supplementary material for: Longitudinal linear combination test for gene set analysis
Source: BMC Bioinformatics. 2019 Dec 10;20:650. doi: 10.1186/s12859-019-3221-7 (PMC6902471; doi:10.1186/s12859-019-3221-7)
Supplement: Supplementary file 2 — Additional file 2. Results of LLCT examining the differential expressions of different gene sets in association with various measures of blood pressure for UNRELATED subjects in GAW19 dataset. [file 12859_2019_3221_MOESM2_ESM.docx]

Additional File 2: Results of LLCT examining the differential expressions of different gene sets in association with various measures of blood pressure for UNRELATED subjects in GAW19 dataset

|  |  | **Gene set size** |  | **adjusted for Smoking Status** | | | | |  | **Adjusted for Antihypertensive Medication** | | | | |  | **No Adjustment** | | | | |
| --- | --- | --- | --- | --- | --- | --- | --- | --- | --- | --- | --- | --- | --- | --- | --- | --- | --- | --- | --- | --- |
|  |  |  |  | **SBP** | **DBP** | **SBP&DBP** | **SBP-DBP** | **HTN** |  | **SBP** | **DBP** | **SBP&DBP** | **SBP-DBP** | **HTN** |  | **SBP** | **DBP** | **DBP&SBP** | **SBP-DBP** | **HTN** |
|  | |  |  |  |  |  |  |  |  |  |  |  |  |  |  |  |  |  |  |  |
| **Molecular function** | |  |  |  |  |  |  |  |  |  |  |  |  |  |  |  |  |  |  |  |
|  | Organic Hydroxy Compound Transmembrane Transporter Activity | 32 | p-value | 0.19 | 0.78 | 0.146 | 0.029** | 0.899 |  | 0.034** | 0.768 | 0.039** | 0.044** | 0.519 |  | 0.09* | 1 | 0.106 | 0.027** | 0.848 |
|  |  |  | q-value | 0.306 | 1.000 | 0.367 | 0.141 | 0.969 |  | 0.578 | 1.000 | 0.531 | 0.579 | 0.555 |  | 0.392 | 1.000 | 0.376 | 0.167 | 0.991 |
|  | Snrna Binding | 31 | p-value | 0.297 | 0.96 | 0.321 | 0.111 | 0.891 |  | 0.017** | 0.632 | 0.017** | 0.014** | 0.293 |  | 0.103 | 0.99 | 0.143 | 0.063* | 0.684 |
|  |  |  | q-value | 0.307 | 1.000 | 0.367 | 0.141 | 0.969 |  | 0.578 | 1.000 | 0.531 | 0.579 | 0.555 |  | 0.392 | 1.000 | 0.376 | 0.167 | 0.991 |
|  | Voltage Gated Calcium Channel Activity | 21 | p-value | 0.038** | 0.62 | 0.044** | 0.012** | 0.532 |  | 0.026** | 0.365 | 0.044** | 0.033** | 0.143 |  | 0.015** | 0.48 | 0.026** | 0.014** | 0.425 |
|  |  |  | q-value | 0.306 | 1.000 | 0.367 | 0.141 | 0.969 |  | 0.578 | 1.000 | 0.531 | 0.579 | 0.555 |  | 0.392 | 1.000 | 0.376 | 0.167 | 0.991 |
|  | |  |  |  |  |  |  |  |  |  |  |  |  |  |  |  |  |  |  |  |
| **Cell Component** | |  |  |  |  |  |  |  |  |  |  |  |  |  |  |  |  |  |  |  |
|  | Copi Coated Vesicle | 22 | p-value | 0.025** | 0.093* | 0.048** | 0.052* | 0.803 |  | 0.007*** | 0.044** | 0.015** | 0.009*** | 0.928 |  | 0.019** | 0.072* | 0.05* | 0.071* | 0.608 |
|  |  |  | q-value | 0.306 | 1.000 | 0.367 | 0.141 | 0.969 |  | 0.578 | 1.000 | 0.531 | 0.579 | 0.557 |  | 0.392 | 1.000 | 0.376 | 0.167 | 0.991 |
|  | Synaptonemal Complex | 17 | p-value | 0.082* | 0.34 | 0.179 | 0.075* | 0.259 |  | 0.013** | 0.148 | 0.036** | 0.02** | 0.178 |  | 0.047** | 0.16 | 0.121 | 0.123 | 0.296 |
|  |  |  | q-value | 0.306 | 1.000 | 0.367 | 0.141 | 0.969 |  | 0.578 | 1.000 | 0.531 | 0.579 | 0.555 |  | 0.392 | 1.000 | 0.376 | 0.167 | 0.991 |
|  | Organellar Small Ribosomal Subunit | 25 | p-value | 0.044** | 0.27 | 0.115 | 0.06* | 0.297 |  | 0.033** | 0.096* | 0.044** | 0.03** | 0.404 |  | 0.023** | 0.1 | 0.056* | 0.068* | 0.307 |
|  |  |  | q-value | 0.306 | 1.000 | 0.367 | 0.141 | 0.969 |  | 0.578 | 1.000 | 0.531 | 0.579 | 0.555 |  | 0.392 | 1.000 | 0.376 | 0.167 | 0.991 |
|  | Cytosolic Ribosome | 97 | p-value | 0.205 | 0.83 | 0.313 | 0.117 | 0.577 |  | 0.03** | 0.194 | 0.044** | 0.026** | 0.046** | | 0.071* | 0.56 | 0.181 | 0.089* | 0.451 |
|  |  |  | q-value | 0.306 | 1.000 | 0.367 | 0.141 | 0.969 |  | 0.578 | 1.000 | 0.531 | 0.579 | 0.555 |  | 0.392 | 1.000 | 0.376 | 0.167 | 0.991 |
|  | |  |  |  |  |  |  |  |  |  |  |  |  |  |  |  |  |  |  |  |
| **Biological Process** | |  |  |  |  |  |  |  |  |  |  |  |  |  |  |  |  |  |  |  |
|  | |  |  |  |  |  |  |  |  |  |  |  |  |  |  |  |  |  |  |  |
| **Immune System Process** | |  |  |  |  |  |  |  |  |  |  |  |  |  |  |  |  |  |  |  |
|  | Regulation Of Inflammatory Response To Antigenic Stimulus | 15 | p-value | 0.016** | 0.19 | 0.033** | 0.007*** | 0.602 |  | 0.015** | 0.118 | 0.018** | 0.011** | 0.626 |  | 0.004*** | 0.087* | 0.019** | 0.017** | 0.710 |
|  |  |  | q-value | 0.306 | 1.000 | 0.367 | 0.141 | 0.969 |  | 0.578 | 1.000 | 0.531 | 0.579 | 0.555 |  | 0.392 | 1.000 | 0.376 | 0.167 | 0.991 |
|  | Negative Regulation Of Toll Like Receptor Signaling Pathway | 16 | p-value | 0.392 | 0.49 | 0.653 | 0.55 | 0.412 |  | 0.018** | 0.066* | 0.045** | 0.026** | 0.251 |  | 0.169 | 0.27 | 0.352 | 0.379 | 0.259 |
|  |  |  | q-value | 0.315 | 1.000 | 0.393 | 0.212 | 0.969 |  | 0.578 | 1.000 | 0.531 | 0.579 | 0.555 |  | 0.392 | 1.000 | 0.376 | 0.194 | 0.991 |
|  | Negative Regulation Of Osteoclast Differentiation | 16 | p-value | 0.059* | 0.18 | 0.113 | 0.107 | 0.279 |  | 0.001*** | 0.03** | 0.009*** | 0.007*** | 0.089* | | 0.031** | 0.17 | 0.074* | 0.071* | 0.272 |
|  |  |  | q-value | 0.306 | 1.000 | 0.367 | 0.141 | 0.969 |  | 0.578 | 1.000 | 0.531 | 0.579 | 0.555 |  | 0.392 | 1.000 | 0.376 | 0.167 | 0.991 |
|  |  |  |  |  |  |  |  |  |  |  |  |  |  |  |  |  |  |  |  |  |
|  | |  |  |  |  |  |  |  |  |  |  |  |  |  |  |  |  |  |  |  |
| **Cellular Process** | |  |  |  |  |  |  |  |  |  |  |  |  |  |  |  |  |  |  |  |
|  | |  |  |  |  |  |  |  |  |  |  |  |  |  |  |  |  |  |  |  |
| **Cellular Process: Cellular Response to Stimulus** | |  |  |  |  |  |  |  |  |  |  |  |  |  |  |  |  |  |  |  |
|  | Response To Ph | 22 | p-value | 0.251 | 0.56 | 0.124 | 0.043** | 0.623 |  | 0.034** | 0.586 | 0.046** | 0.039** | 0.71 |  | 0.048** | 0.96 | 0.048** | 0.012** | 0.328 |
|  |  |  | q-value | 0.306 | 1.000 | 0.367 | 0.141 | 0.969 |  | 0.578 | 1.000 | 0.531 | 0.579 | 0.555 |  | 0.392 | 1.000 | 0.376 | 0.167 | 0.991 |
|  | Regulation Of Chemotaxis | 119 | p-value | 0.136 | 0.92 | 0.114 | 0.027** | 0.465 |  | 0.04** | 0.624 | 0.039** | 0.028** | 0.153 |  | 0.047** | 1 | 0.068* | 0.028** | 0.341 |
|  |  |  | q-value | 0.306 | 1.000 | 0.367 | 0.141 | 0.969 |  | 0.578 | 1.000 | 0.531 | 0.579 | 0.555 |  | 0.392 | 1.000 | 0.376 | 0.167 | 0.991 |
|  | |  |  |  |  |  |  |  |  |  |  |  |  |  |  |  |  |  |  |  |
| **Cellular Process: Cell Communication** | |  |  |  |  |  |  |  |  |  |  |  |  |  |  |  |  |  |  |  |
|  | |  |  |  |  |  |  |  |  |  |  |  |  |  |  |  |  |  |  |  |
| **Cellular Process: Cell Communication: Cell-cell Signaling** | |  |  |  |  |  |  |  |  |  |  |  |  |  |  |  |  |  |  |  |
|  | Beta Catenin Destruction Complex Disassembly | 16 | p-value | 0.094* | 0.33 | 0.157 | 0.093* | 0.424 |  | 0.027** | 0.078* | 0.032** | 0.03** | 0.33 |  | 0.038** | 0.14 | 0.085* | 0.109 | 0.375 |
|  |  |  | q-value | 0.306 | 1.000 | 0.367 | 0.141 | 0.969 |  | 0.578 | 1.000 | 0.531 | 0.579 | 0.555 |  | 0.392 | 1.000 | 0.376 | 0.167 | 0.991 |
| **Cellular Process: Cell Communication: Signal Transduction** | |  |  |  |  |  |  |  |  |  |  |  |  |  |  |  |  |  |  |  |
|  | G Protein Coupled Receptor Signaling Pathway Coupled To Cyclic Nucleotide Second Messenger | 71 | p-value | 0.068* | 0.62 | 0.088* | 0.03** | 0.473 |  | 0.041** | 0.379 | 0.045** | 0.038** | 0.271 |  | 0.034** | 0.63 | 0.064* | 0.029** | 0.422 |
|  |  |  | q-value | 0.306 | 1.000 | 0.367 | 0.141 | 0.969 |  | 0.578 | 1.000 | 0.531 | 0.579 | 0.555 |  | 0.392 | 1.000 | 0.376 | 0.167 | 0.991 |
|  | |  |  |  |  |  |  |  |  |  |  |  |  |  |  |  |  |  |  |  |
| **Cellular Process: Cell Communication: Others** | |  |  |  |  |  |  |  |  |  |  |  |  |  |  |  |  |  |  |  |
|  | Cellular Response To Starvation | 83 | p-value | 0.038** | 0.28 | 0.079* | 0.03** | 0.766 |  | 0.019** | 0.079* | 0.028** | 0.02** | 0.582 |  | 0.02** | 0.13 | 0.039** | 0.032** | 0.744 |
|  |  |  | q-value | 0.306 | 1.000 | 0.367 | 0.141 | 0.969 |  | 0.578 | 1.000 | 0.531 | 0.579 | 0.555 |  | 0.392 | 1.000 | 0.376 | 0.167 | 0.991 |
|  | |  |  |  |  |  |  |  |  |  |  |  |  |  |  |  |  |  |  |  |
| **Cellular Process: Cellular Metabolic Process** | |  |  |  |  |  |  |  |  |  |  |  |  |  |  |  |  |  |  |  |
|  | Regulation Of Protein Deacetylation | 22 | p-value | 0.086* | 0.67 | 0.095* | 0.031** | 0.381 |  | 0.018** | 0.098* | 0.041** | 0.023** | 0.202 |  | 0.045** | 0.33 | 0.105 | 0.052* | 0.463 |
|  |  |  | q-value | 0.306 | 1.000 | 0.367 | 0.141 | 0.969 |  | 0.578 | 1.000 | 0.531 | 0.579 | 0.555 |  | 0.392 | 1.000 | 0.376 | 0.167 | 0.991 |
|  | Regulation Of Receptor Internalization | 23 | p-value | 0.267 | 0.95 | 0.277 | 0.128 | 0.948 |  | 0.015** | 0.188 | 0.023** | 0.009*** | 0.892 |  | 0.068* | 0.53 | 0.14 | 0.05* | 0.722 |
|  |  |  | q-value | 0.306 | 1.000 | 0.367 | 0.141 | 0.969 |  | 0.578 | 1.000 | 0.531 | 0.579 | 0.555 |  | 0.392 | 1.000 | 0.376 | 0.167 | 0.991 |
|  | Pteridine Containing Compound Metabolic Process | 24 | p-value | 0.092* | 0.77 | 0.127 | 0.037** | 0.221 |  | 0.018** | 0.294 | 0.038** | 0.021** | 0.691 |  | 0.055* | 0.65 | 0.118 | 0.047** | 0.245 |
|  |  |  | q-value | 0.306 | 1.000 | 0.367 | 0.141 | 0.969 |  | 0.578 | 1.000 | 0.531 | 0.579 | 0.555 |  | 0.392 | 1.000 | 0.376 | 0.167 | 0.991 |
|  | Peptidyl Lysine Trimethylation | 21 | p-value | 0.086* | 0.76 | 0.081* | 0.019** | 0.972 |  | 0.022** | 0.306 | 0.031** | 0.019** | 0.931 |  | 0.021** | 0.57 | 0.037** | 0.009*** | 0.779 |
|  |  |  | q-value | 0.306 | 1.000 | 0.367 | 0.141 | 0.972 |  | 0.578 | 1.000 | 0.531 | 0.579 | 0.557 |  | 0.392 | 1.000 | 0.376 | 0.167 | 0.991 |
|  | |  |  |  |  |  |  |  |  |  |  |  |  |  |  |  |  |  |  |  |
| **Cellular Process: Multi-organism Cellular Process** | |  |  |  |  |  |  |  |  |  |  |  |  |  |  |  |  |  |  |  |
|  | Multi Organism Organelle Organization | 19 | p-value | 0.101 | 0.35 | 0.177 | 0.11 | 0.629 |  | 0.024** | 0.155 | 0.039** | 0.033** | 0.833 |  | 0.04** | 0.22 | 0.11 | 0.078* | 0.761 |
|  |  |  | q-value | 0.306 | 1.000 | 0.367 | 0.141 | 0.969 |  | 0.578 | 1.000 | 0.531 | 0.579 | 0.555 |  | 0.392 | 1.000 | 0.376 | 0.167 | 0.991 |
|  | |  |  |  |  |  |  |  |  |  |  |  |  |  |  |  |  |  |  |  |
|  | |  |  |  |  |  |  |  |  |  |  |  |  |  |  |  |  |  |  |  |
| **Cellular Process: Others** | |  |  |  |  |  |  |  |  |  |  |  |  |  |  |  |  |  |  |  |
|  | B Cell Proliferation | 20 | p-value | 0.036** | 0.22 | 0.075* | 0.027** | 0.395 |  | 0.017** | 0.093* | 0.044** | 0.025** | 0.481 |  | 0.019** | 0.13 | 0.037** | 0.046** | 0.378 |
|  |  |  | q-value | 0.306 | 1.000 | 0.367 | 0.141 | 0.969 |  | 0.578 | 1.000 | 0.531 | 0.579 | 0.555 |  | 0.392 | 1.000 | 0.376 | 0.167 | 0.991 |
|  | Negative Regulation Of Muscle Cell Apoptotic Process | 19 | p-value | 0.086* | 0.73 | 0.095* | 0.021** | 0.807 |  | 0.027** | 0.524 | 0.042** | 0.038** | 0.336 |  | 0.038** | 0.63 | 0.069* | 0.019** | 0.877 |
|  |  |  | q-value | 0.306 | 1.000 | 0.367 | 0.141 | 0.969 |  | 0.578 | 1.000 | 0.531 | 0.579 | 0.555 |  | 0.392 | 1.000 | 0.376 | 0.167 | 0.991 |
|  | Positive Regulation Of T Helper Cell Differentiation | 16 | p-value | 0.032** | 0.3 | 0.055* | 0.021** | 0.942 |  | 0.027** | 0.282 | 0.034** | 0.036** | 0.844 |  | 0.013** | 0.19 | 0.033** | 0.022** | 0.982 |
|  |  |  | q-value | 0.306 | 1.000 | 0.367 | 0.141 | 0.969 |  | 0.578 | 1.000 | 0.531 | 0.579 | 0.555 |  | 0.392 | 1.000 | 0.376 | 0.167 | 0.993 |
|  | |  |  |  |  |  |  |  |  |  |  |  |  |  |  |  |  |  |  |  |
| **Multicellular Organismal Process** | |  |  |  |  |  |  |  |  |  |  |  |  |  |  |  |  |  |  |  |
|  | Regulation Of Bone Resorption | 18 | p-value | 0.538 | 0.2 | 0.07* | 0.063* | 0.950 |  | 0.023** | 0.27 | 0.018** | 0.013** | 0.904 |  | 0.023** | 0.98 | 0.04** | 0.021** | 0.387 |
|  |  |  | q-value | 0.345 | 1.000 | 0.367 | 0.141 | 0.969 |  | 0.578 | 1.000 | 0.531 | 0.579 | 0.555 |  | 0.392 | 1.000 | 0.376 | 0.167 | 0.991 |
|  | |  |  |  |  |  |  |  |  |  |  |  |  |  |  |  |  |  |  |  |
| **Multicellular Organismal Process: System Process** | |  |  |  |  |  |  |  |  |  |  |  |  |  |  |  |  |  |  |  |
|  | Regulation Of Vasodilation | 27 | p-value | 0.059* | 0.25 | 0.125 | 0.088* | 0.924 |  | 0.02** | 0.222 | 0.025** | 0.015** | 0.838 |  | 0.023** | 0.18 | 0.065* | 0.056* | 0.883 |
|  |  |  | q-value | 0.306 | 1.000 | 0.367 | 0.141 | 0.969 |  | 0.578 | 1.000 | 0.531 | 0.579 | 0.555 |  | 0.392 | 1.000 | 0.376 | 0.167 | 0.991 |
|  | |  |  |  |  |  |  |  |  |  |  |  |  |  |  |  |  |  |  |  |
| **Metabolic Process** | |  |  |  |  |  |  |  |  |  |  |  |  |  |  |  |  |  |  |  |
|  | Multicellular Organism Metabolic Process | 42 | p-value | 0.132 | 0.52 | 0.228 | 0.135 | 0.851 |  | 0.009*** | 0.112 | 0.022** | 0.012** | 0.183 |  | 0.055* | 0.3 | 0.145 | 0.107 | 0.716 |
|  |  |  | q-value | 0.306 | 1.000 | 0.367 | 0.141 | 0.969 |  | 0.578 | 1.000 | 0.531 | 0.579 | 0.555 |  | 0.392 | 1.000 | 0.376 | 0.167 | 0.991 |

*Significance level of 0.1

**Significant level of 0.05

***Significance level of 0.001

† The multiple analysis of systolic and diastolic blood pressure measurements

‡The pulse pressure: difference between systolic and diastolic blood pressure values
