# Supplementary material for: Longitudinal linear combination test for gene set analysis
Source: BMC Bioinformatics. 2019 Dec 10;20:650. doi: 10.1186/s12859-019-3221-7 (PMC6902471; doi:10.1186/s12859-019-3221-7)
Supplement: Supplementary file 3 — Additional file 3. Results of LLCT examining the differential expressions of different gene sets in association with various measures of blood pressure for RELATED subjects in GAW19 dataset. [file 12859_2019_3221_MOESM3_ESM.docx]

Additional File 3: Results of LLCT examining the differential expressions of different gene sets in association with various measures of blood pressure for RELATED subjects in GAW19 dataset

|  |  | **GS size** |  | **adjusted for Smoking Status** | | | | |  | **Adjusted for Antihypertensive Medication** | | | | |  | **No Adjustment** | | | | |
| --- | --- | --- | --- | --- | --- | --- | --- | --- | --- | --- | --- | --- | --- | --- | --- | --- | --- | --- | --- | --- |
|  |  |  |  | **SBP** | **DBP** | **SBP&DBP** | **SBP-DBP** | **HTN** |  | **SBP** | **DBP** | **SBP&DBP** | **SBP-DBP** | **HTN** |  | **SBP** | **DBP** | **DBP&SBP** | **SBP-DBP** | **HTN** |
| **Molecular fuction** | |  |  |  |  |  |  |  |  |  |  |  |  |  |  |  |  |  |  |  |
| **Binding** | |  |  |  |  |  |  |  |  |  |  |  |  |  |  |  |  |  |  |  |
|  | Flavin Adenine Dinucleotide Binding | 49 | p-value | 0.568 | 0.161 | 0.105 | 0.051* | 0.099* |  | 0.081* | 0.218 | 0.031** | 0.008*** | 0.323 |  | 0.507 | 0.171 | 0.152 | 0.063* | 0.069* |
|  |  |  | q-value | 0.850 | 0.952 | 0.743 | 0.337 | 0.351 |  | 0.287 | 0.997 | 0.424 | 0.085 | 1.000 |  | 0.849 | 0.830 | 0.778 | 0.516 | 0.269 |
|  | Antigen Binding | 65 | p-value | 0.063* | 0.303 | 0.162 | 0.065* | 0.17 |  | 0.006*** | 0.283 | 0.012** | 0.008*** | 0.179 |  | 0.067* | 0.293 | 0.177 | 0.087* | 0.159 |
|  |  |  | q-value | 0.850 | 0.952 | 0.743 | 0.341 | 0.355 |  | 0.234 | 0.997 | 0.424 | 0.085 | 1.000 |  | 0.849 | 0.830 | 0.778 | 0.516 | 0.269 |
|  | Basal Transcription Machinery Binding | 24 | p-value | 0.78 | 0.014** | 0.017** | 0.081* | 0.251 |  | 0.604 | 0.031** | 0.026** | 0.048** | 0.236 |  | 0.738 | 0.023** | 0.024** | 0.125 | 0.187 |
|  |  |  | q-value | 0.850 | 0.952 | 0.743 | 0.342 | 0.361 |  | 0.388 | 0.997 | 0.424 | 0.088 | 1.000 |  | 0.849 | 0.830 | 0.778 | 0.516 | 0.270 |
|  | Single Stranded Dna Binding | 74 | p-value | 0.417 | 0.007*** | 0.021** | 0.218 | 0.055* |  | 0.291 | 0.024** | 0.025** | 0.039** | 0.236 |  | 0.436 | 0.015** | 0.027** | 0.243 | 0.051* |
|  |  |  | q-value | 0.850 | 0.952 | 0.743 | 0.370 | 0.351 |  | 0.332 | 0.997 | 0.424 | 0.087 | 1.000 |  | 0.849 | 0.830 | 0.778 | 0.517 | 0.269 |
|  | Integrin Binding | 62 | p-value | 0.066* | 0.915 | 0.061* | 0.009*** | 0.329 |  | 0.002*** | 0.833 | 0.001*** | 0.001*** | 0.568 |  | 0.087* | 0.838 | 0.095* | 0.017** | 0.247 |
|  |  |  | q-value | 0.850 | 0.952 | 0.743 | 0.334 | 0.369 |  | 0.182 | 0.997 | 0.267 | 0.050 | 1.000 |  | 0.849 | 0.831 | 0.778 | 0.516 | 0.279 |
|  | Damaged Dna Binding | 53 | p-value | 0.113 | 0.797 | 0.204 | 0.106 | 0.34 |  | 0.009*** | 0.776 | 0.031** | 0.02** | 0.605 |  | 0.141 | 0.798 | 0.307 | 0.134 | 0.263 |
|  |  |  | q-value | 0.850 | 0.952 | 0.743 | 0.353 | 0.369 |  | 0.234 | 0.997 | 0.424 | 0.085 | 1.000 |  | 0.849 | 0.830 | 0.778 | 0.516 | 0.279 |
|  | Snap Receptor Activity | 35 | p-value | 0.207 | 0.161 | 0.012** | 0*** | 0.567 |  | 0.085* | 0.298 | 0.001*** | 0*** | 0.865 |  | 0.239 | 0.201 | 0.016** | 0.003*** | 0.429 |
|  |  |  | q-value | 0.850 | 0.952 | 0.743 | 0.000 | 0.391 |  | 0.290 | 0.997 | 0.267 | 0.000 | 1.000 |  | 0.849 | 0.830 | 0.778 | 0.516 | 0.292 |
|  | Transcription Cofactor Binding | 18 | p-value | 0.297 | 0.187 | 0.08* | 0.019** | 0.192 |  | 0.053* | 0.32 | 0.016** | 0.004*** | 0.872 |  | 0.31 | 0.234 | 0.094* | 0.033** | 0.161 |
|  |  |  | q-value | 0.850 | 0.952 | 0.743 | 0.337 | 0.355 |  | 0.270 | 0.997 | 0.424 | 0.076 | 1.000 |  | 0.849 | 0.830 | 0.778 | 0.516 | 0.269 |
|  | Growth Factor Activity | 66 | p-value | 0.003*** | 0.37 | 0.012** | 0.019** | 0.417 |  | 0*** | 0.221 | 0*** | 0.011** | 0.03** |  | 0.002*** | 0.303 | 0.01** | 0.015** | 0.414 |
|  |  |  | q-value | 0.850 | 0.952 | 0.743 | 0.337 | 0.375 |  | 0.000 | 0.997 | 0.000 | 0.085 | 1.000 |  | 0.849 | 0.830 | 0.778 | 0.516 | 0.291 |
|  | Heat Shock Protein Binding | 74 | p-value | 0.462 | 0.052* | 0.084* | 0.125 | 0.016** |  | 0.181 | 0.118 | 0.049** | 0.015** | 0.505 |  | 0.425 | 0.068* | 0.131 | 0.187 | 0.019** |
|  |  |  | q-value | 0.850 | 0.952 | 0.743 | 0.355 | 0.343 |  | 0.314 | 0.997 | 0.424 | 0.085 | 1.000 |  | 0.849 | 0.830 | 0.778 | 0.516 | 0.269 |
|  | Actin Binding | 262 | p-value | 0.189 | 0.512 | 0.399 | 0.208 | 0.3 |  | 0.012** | 0.519 | 0.049** | 0.035** | 0.669 |  | 0.205 | 0.558 | 0.442 | 0.199 | 0.198 |
|  |  |  | q-value | 0.850 | 0.952 | 0.743 | 0.369 | 0.368 |  | 0.234 | 0.997 | 0.424 | 0.087 | 1.000 |  | 0.849 | 0.830 | 0.778 | 0.517 | 0.271 |
|  | |  |  |  |  |  |  |  |  |  |  |  |  |  |  |  |  |  |  |  |
| **Catalytic Activity** | |  |  |  |  |  |  |  |  |  |  |  |  |  |  |  |  |  |  |  |
|  | Hydrolase Activity Hydrolyzing N Glycosyl Compounds | 19 | p-value | 0.49 | 0.507 | 0.294 | 0.076* | 0.219 |  | 0.061* | 0.676 | 0.049** | 0.012** | 0.633 |  | 0.482 | 0.593 | 0.313 | 0.086* | 0.207 |
|  |  |  | q-value | 0.850 | 0.952 | 0.743 | 0.342 | 0.355 |  | 0.280 | 0.997 | 0.424 | 0.085 | 1.000 |  | 0.849 | 0.830 | 0.778 | 0.516 | 0.273 |
|  | Metalloendopeptidase Activity | 62 | p-value | 0.224 | 0.265 | 0.086* | 0.009*** | 0.35 |  | 0.214 | 0.301 | 0.042** | 0.01** | 0.902 |  | 0.294 | 0.254 | 0.082* | 0.013** | 0.297 |
|  |  |  | q-value | 0.850 | 0.952 | 0.743 | 0.334 | 0.369 |  | 0.321 | 0.997 | 0.424 | 0.085 | 1.000 |  | 0.849 | 0.830 | 0.778 | 0.516 | 0.280 |
|  |  |  |  |  |  |  |  |  |  |  |  |  |  |  |  |  |  |  |  |  |
|  | |  |  |  |  |  |  |  |  |  |  |  |  |  |  |  |  |  |  |  |
| **Cell Component** | |  |  |  |  |  |  |  |  |  |  |  |  |  |  |  |  |  |  |  |
|  | |  |  |  |  |  |  |  |  |  |  |  |  |  |  |  |  |  |  |  |
| **Organelle** | |  |  |  |  |  |  |  |  |  |  |  |  |  |  |  |  |  |  |  |
|  | Vesicle Membrane | 337 | p-value | 0.414 | 0.271 | 0.229 | 0.04** | 0.116 |  | 0.2 | 0.445 | 0.033** | 0.002*** | 0.961 |  | 0.432 | 0.26 | 0.242 | 0.04** | 0.115 |
|  |  |  | q-value | 0.850 | 0.952 | 0.743 | 0.337 | 0.352 |  | 0.317 | 0.997 | 0.424 | 0.065 | 1.000 |  | 0.849 | 0.830 | 0.778 | 0.516 | 0.269 |
|  | Organellar Large Ribosomal Subunit | 31 | p-value | 0.141 | 0.137 | 0.041** | 0.007*** | 0.35 |  | 0.047** | 0.13 | 0.039** | 0.005*** | 0.296 |  | 0.14 | 0.121 | 0.039** | 0.005*** | 0.325 |
|  |  |  | q-value | 0.850 | 0.952 | 0.743 | 0.334 | 0.369 |  | 0.268 | 0.997 | 0.424 | 0.076 | 1.000 |  | 0.849 | 0.830 | 0.778 | 0.516 | 0.282 |
|  | Vacuolar Membrane | 441 | p-value | 0.352 | 0.216 | 0.093* | 0.023** | 0.471 |  | 0.169 | 0.317 | 0.043** | 0.017** | 0.074* |  | 0.355 | 0.24 | 0.131 | 0.044** | 0.453 |
|  |  |  | q-value | 0.850 | 0.952 | 0.743 | 0.337 | 0.382 |  | 0.314 | 0.997 | 0.424 | 0.085 | 1.000 |  | 0.849 | 0.830 | 0.778 | 0.516 | 0.294 |
|  | |  |  |  |  |  |  |  |  |  |  |  |  |  |  |  |  |  |  |  |
| **cell: intrecellular** | |  |  |  |  |  |  |  |  |  |  |  |  |  |  |  |  |  |  |  |
|  | U2 Type Spliceosomal Complex | 24 | p-value | 0.318 | 0.906 | 0.304 | 0.085* | 0.14 |  | 0.013** | 0.92 | 0.023** | 0.012** | 0.803 |  | 0.287 | 0.871 | 0.287 | 0.092* | 0.122 |
|  |  |  | q-value | 0.850 | 0.952 | 0.743 | 0.342 | 0.355 |  | 0.237 | 0.997 | 0.424 | 0.085 | 1.000 |  | 0.849 | 0.831 | 0.778 | 0.516 | 0.269 |
|  | Inclusion Body | 56 | p-value | 0.029** | 0.701 | 0.052* | 0.01** | 0.619 |  | 0.004*** | 0.698 | 0.016** | 0.009*** | 0.203 |  | 0.037** | 0.685 | 0.068* | 0.022** | 0.604 |
|  |  |  | q-value | 0.850 | 0.952 | 0.743 | 0.334 | 0.396 |  | 0.223 | 0.997 | 0.424 | 0.085 | 1.000 |  | 0.849 | 0.830 | 0.778 | 0.516 | 0.311 |
|  | Coated Vesicle Membrane | 108 | p-value | 0.06* | 0.575 | 0.064* | 0.004*** | 0.807 |  | 0.006*** | 0.621 | 0.008*** | 0.002*** | 0.18 |  | 0.073* | 0.623 | 0.092* | 0.018** | 0.723 |
|  |  |  | q-value | 0.850 | 0.952 | 0.743 | 0.316 | 0.426 |  | 0.234 | 0.997 | 0.424 | 0.065 | 1.000 |  | 0.849 | 0.830 | 0.778 | 0.516 | 0.327 |
|  | Vacuolar Part | 508 | p-value | 0.022** | 0.222 | 0.045** | 0.025** | 0.516 |  | 0.01** | 0.243 | 0.026** | 0.024** | 0.731 |  | 0.016** | 0.181 | 0.043** | 0.04** | 0.45 |
|  |  |  | q-value | 0.850 | 0.952 | 0.743 | 0.337 | 0.386 |  | 0.234 | 0.997 | 0.424 | 0.086 | 1.000 |  | 0.849 | 0.830 | 0.778 | 0.516 | 0.294 |
|  | Intrinsic Component Of Mitochondrial Inner Membrane | 17 | p-value | 0.016** | 0.656 | 0.075* | 0.057* | 0.148 |  | 0.007*** | 0.665 | 0.022** | 0.021** | 0.237 |  | 0.03** | 0.714 | 0.086* | 0.053* | 0.151 |
|  |  |  | q-value | 0.850 | 0.952 | 0.743 | 0.337 | 0.355 |  | 0.234 | 0.997 | 0.424 | 0.085 | 1.000 |  | 0.849 | 0.830 | 0.778 | 0.516 | 0.269 |
|  | Cyclin Dependent Protein Kinase Holoenzyme Complex | 26 | p-value | 0.045** | 0.602 | 0.078* | 0.008*** | 0.216 |  | 0.016** | 0.656 | 0.021** | 0.009*** | 0.397 |  | 0.074* | 0.542 | 0.088* | 0.012** | 0.223 |
|  |  |  | q-value | 0.850 | 0.952 | 0.743 | 0.334 | 0.355 |  | 0.262 | 0.997 | 0.424 | 0.085 | 1.000 |  | 0.849 | 0.830 | 0.778 | 0.516 | 0.276 |
|  | |  |  |  |  |  |  |  |  |  |  |  |  |  |  |  |  |  |  |  |
| **cell: intracellular: intracellular organelle** | |  |  |  |  |  |  |  |  |  |  |  |  |  |  |  |  |  |  |  |
|  | Intermediate Filament | 52 | p-value | 0.001*** | 0.82 | 0.006*** | 0.003*** | 0.244 |  | 0.001*** | 0.781 | 0.003*** | 0.006*** | 0.266 |  | 0.001*** | 0.788 | 0.011** | 0.002*** | 0.333 |
|  |  |  | q-value | 0.850 | 0.952 | 0.743 | 0.316 | 0.359 |  | 0.144 | 0.997 | 0.424 | 0.081 | 1.000 |  | 0.849 | 0.830 | 0.778 | 0.516 | 0.283 |
|  | Centrosome | 378 | p-value | 0.071* | 0.812 | 0.159 | 0.061* | 0.516 |  | 0.005*** | 0.795 | 0.027** | 0.018** | 0.331 |  | 0.069* | 0.855 | 0.179 | 0.074* | 0.445 |
|  |  |  | q-value | 0.850 | 0.952 | 0.743 | 0.337 | 0.386 |  | 0.234 | 0.997 | 0.424 | 0.085 | 1.000 |  | 0.849 | 0.831 | 0.778 | 0.516 | 0.293 |
|  | Synaptonemal Complex | 17 | p-value | 0.119 | 0.002*** | 0.004*** | 0.003*** | 0.013** |  | 0.076* | 0.011** | 0.001*** | 0.001*** | 0.111 |  | 0.119 | 0.006*** | 0.009*** | 0.006*** | 0.016** |
|  |  |  | q-value | 0.850 | 0.952 | 0.743 | 0.316 | 0.343 |  | 0.285 | 0.997 | 0.267 | 0.050 | 1.000 |  | 0.849 | 0.830 | 0.778 | 0.516 | 0.269 |
|  | Transcription Elongation Factor Complex | 45 | p-value | 0.045** | 0.485 | 0.097* | 0.035** | 0.628 |  | 0.008*** | 0.394 | 0.017** | 0.015** | 0.207 |  | 0.027** | 0.406 | 0.089* | 0.059* | 0.579 |
|  |  |  | q-value | 0.850 | 0.952 | 0.743 | 0.337 | 0.398 |  | 0.234 | 0.997 | 0.424 | 0.085 | 1.000 |  | 0.849 | 0.830 | 0.778 | 0.516 | 0.308 |
|  | Cop9 Signalosome | 30 | p-value | 0.041** | 0.846 | 0.018** | 0.002*** | 0.517 |  | 0.048** | 0.8 | 0.032** | 0.01** | 0.979 |  | 0.046** | 0.825 | 0.025** | 0.004*** | 0.523 |
|  |  |  | q-value | 0.850 | 0.952 | 0.743 | 0.257 | 0.386 |  | 0.268 | 0.997 | 0.424 | 0.085 | 1.000 |  | 0.849 | 0.830 | 0.778 | 0.516 | 0.301 |
|  | |  |  |  |  |  |  |  |  |  |  |  |  |  |  |  |  |  |  |  |
| **endomembrane system** | |  |  |  |  |  |  |  |  |  |  |  |  |  |  |  |  |  |  |  |
|  | Platelet Alpha Granule | 52 | p-value | 0.023** | 0.7 | 0.059* | 0.022** | 0.699 |  | 0*** | 0.487 | 0.004*** | 0.008*** | 0.092* |  | 0.031** | 0.662 | 0.075* | 0.028** | 0.664 |
|  |  |  | q-value | 0.850 | 0.952 | 0.743 | 0.337 | 0.408 |  | 0.000 | 0.997 | 0.424 | 0.085 | 1.000 |  | 0.849 | 0.830 | 0.778 | 0.516 | 0.319 |
|  | Recycling Endosome Membrane | 28 | p-value | 0.051* | 0.846 | 0.146 | 0.037** | 0.828 |  | 0.007*** | 0.744 | 0.03** | 0.022** | 0.096* |  | 0.065* | 0.82 | 0.18 | 0.069* | 0.767 |
|  |  |  | q-value | 0.850 | 0.952 | 0.743 | 0.337 | 0.430 |  | 0.234 | 0.997 | 0.424 | 0.085 | 1.000 |  | 0.849 | 0.830 | 0.778 | 0.516 | 0.334 |
|  | Recycling Endosome | 88 | p-value | 0.073* | 0.812 | 0.099* | 0.033** | 0.5 |  | 0.004*** | 0.693 | 0.01** | 0.004*** | 0.21 |  | 0.091* | 0.752 | 0.148 | 0.065* | 0.354 |
|  |  |  | q-value | 0.850 | 0.952 | 0.743 | 0.337 | 0.385 |  | 0.223 | 0.997 | 0.424 | 0.076 | 1.000 |  | 0.849 | 0.830 | 0.778 | 0.516 | 0.283 |
|  | |  |  |  |  |  |  |  |  |  |  |  |  |  |  |  |  |  |  |  |
| **membrane** | |  |  |  |  |  |  |  |  |  |  |  |  |  |  |  |  |  |  |  |
|  | Clathrin Coat | 42 | p-value | 0.147 | 0.583 | 0.033** | 0.002*** | 0.203 |  | 0.064* | 0.695 | 0.02** | 0.001*** | 0.955 |  | 0.183 | 0.553 | 0.059* | 0.006*** | 0.167 |
|  |  |  | q-value | 0.850 | 0.952 | 0.743 | 0.257 | 0.355 |  | 0.280 | 0.997 | 0.424 | 0.050 | 1.000 |  | 0.849 | 0.830 | 0.778 | 0.516 | 0.269 |
|  | Extrinsic Component Of Cytoplasmic Side Of Plasma Membrane | 63 | p-value | 0.076* | 0.093* | 0.216 | 0.05* | 0.013** |  | 0.013** | 0.256 | 0.014** | 0.002*** | 0.253 |  | 0.087* | 0.156 | 0.222 | 0.052* | 0.023** |
|  |  |  | q-value | 0.850 | 0.952 | 0.743 | 0.337 | 0.343 |  | 0.237 | 0.997 | 0.424 | 0.065 | 1.000 |  | 0.849 | 0.830 | 0.778 | 0.516 | 0.269 |
|  | |  |  |  |  |  |  |  |  |  |  |  |  |  |  |  |  |  |  |  |
| **Others** | |  |  |  |  |  |  |  |  |  |  |  |  |  |  |  |  |  |  |  |
|  | Excitatory Synapse | 105 | p-value | 0.026** | 0.906 | 0.044** | 0.01** | 0.95 |  | 0.012** | 0.846 | 0.006*** | 0.004*** | 0.254 |  | 0.03** | 0.911 | 0.064* | 0.006*** | 0.92 |
|  |  |  | q-value | 0.850 | 0.952 | 0.743 | 0.334 | 0.456 |  | 0.234 | 0.997 | 0.424 | 0.076 | 1.000 |  | 0.849 | 0.832 | 0.778 | 0.516 | 0.363 |
|  | Lamellipodium | 122 | p-value | 0.123 | 0.281 | 0.037** | 0.002*** | 0.018** |  | 0.012** | 0.434 | 0.002*** | 0*** | 0.98 |  | 0.139 | 0.313 | 0.057* | 0.007*** | 0.016** |
|  |  |  | q-value | 0.850 | 0.952 | 0.743 | 0.257 | 0.343 |  | 0.234 | 0.997 | 0.424 | 0.000 | 1.000 |  | 0.849 | 0.830 | 0.778 | 0.516 | 0.269 |
|  | Intercellular Bridge | 37 | p-value | 0.119 | 0.717 | 0.234 | 0.086* | 0.176 |  | 0.012** | 0.661 | 0.024** | 0.02** | 0.179 |  | 0.104 | 0.651 | 0.252 | 0.104 | 0.184 |
|  |  |  | q-value | 0.850 | 0.952 | 0.743 | 0.342 | 0.355 |  | 0.234 | 0.997 | 0.424 | 0.085 | 1.000 |  | 0.849 | 0.830 | 0.778 | 0.516 | 0.269 |
|  | Presynapse | 163 | p-value | 0.262 | 0.735 | 0.377 | 0.077* | 0.17 |  | 0.006*** | 0.596 | 0.008*** | 0.004*** | 0.075* |  | 0.249 | 0.688 | 0.387 | 0.098* | 0.166 |
|  |  |  | q-value | 0.850 | 0.952 | 0.743 | 0.342 | 0.355 |  | 0.234 | 0.997 | 0.424 | 0.076 | 1.000 |  | 0.849 | 0.830 | 0.778 | 0.516 | 0.269 |
|  | |  |  |  |  |  |  |  |  |  |  |  |  |  |  |  |  |  |  |  |
| **Biological Process** | |  |  |  |  |  |  |  |  |  |  |  |  |  |  |  |  |  |  |  |
|  | |  |  |  |  |  |  |  |  |  |  |  |  |  |  |  |  |  |  |  |
| **developmental process: multicellular organismal process: system development** | |  |  |  |  |  |  |  |  |  |  |  |  |  |  |  |  |  |  |  |
|  | Cardiac Chamber Development | 78 | p-value | 0.393 | 0.863 | 0.642 | 0.246 | 0.129 |  | 0.017** | 0.716 | 0.046** | 0.03** | 0.396 |  | 0.395 | 0.828 | 0.653 | 0.278 | 0.111 |
|  |  |  | q-value | 0.850 | 0.952 | 0.748 | 0.377 | 0.352 |  | 0.262 | 0.997 | 0.424 | 0.087 | 1.000 |  | 0.849 | 0.830 | 0.779 | 0.517 | 0.269 |
|  | Endothelial Cell Development | 36 | p-value | 0.237 | 0.838 | 0.46 | 0.256 | 0.342 |  | 0.016** | 0.661 | 0.045** | 0.044** | 0.447 |  | 0.232 | 0.827 | 0.471 | 0.254 | 0.33 |
|  |  |  | q-value | 0.850 | 0.952 | 0.743 | 0.378 | 0.369 |  | 0.262 | 0.997 | 0.424 | 0.087 | 1.000 |  | 0.849 | 0.830 | 0.778 | 0.517 | 0.282 |
|  | Coronary Vasculature Development | 24 | p-value | 0.34 | 0.64 | 0.225 | 0.035** | 0.294 |  | 0.02** | 0.699 | 0.027** | 0.006*** | 0.409 |  | 0.331 | 0.653 | 0.235 | 0.066* | 0.242 |
|  |  |  | q-value | 0.850 | 0.952 | 0.743 | 0.337 | 0.368 |  | 0.264 | 0.997 | 0.424 | 0.081 | 1.000 |  | 0.849 | 0.830 | 0.778 | 0.516 | 0.279 |
|  | Pituitary Gland Development | 16 | p-value | 0.278 | 0*** | 0.003*** | 0.51 | 0.003*** |  | 0.092* | 0*** | 0.004*** | 0.046** | 0.006*** |  | 0.228 | 0.002*** | 0.006*** | 0.575 | 0.008*** |
|  |  |  | q-value | 0.850 | 0.000 | 0.743 | 0.427 | 0.320 |  | 0.294 | 0.000 | 0.424 | 0.087 | 1.000 |  | 0.849 | 0.830 | 0.778 | 0.563 | 0.269 |
|  | Ventral Spinal Cord Development | 19 | p-value | 0.688 | 0.083* | 0.045** | 0.045** | 0.271 |  | 0.345 | 0.147 | 0.039** | 0.018** | 0.779 |  | 0.714 | #N/A | 0.054* | 0.053* | 0.283 |
|  |  |  | q-value | 0.850 | 0.952 | 0.743 | 0.337 | 0.363 |  | 0.342 | 0.997 | 0.424 | 0.085 | 1.000 |  | 0.849 | 0.830 | 0.778 | 0.516 | 0.280 |
|  | Digestive System Development | 67 | p-value | 0.02** | 0.397 | 0.066* | 0.068* | 0.593 |  | 0.007*** | 0.264 | 0.018** | 0.049** | 0.111 |  | 0.026** | 0.313 | 0.057* | 0.084* | 0.662 |
|  |  |  | q-value | 0.850 | 0.952 | 0.743 | 0.342 | 0.395 |  | 0.234 | 0.997 | 0.424 | 0.088 | 1.000 |  | 0.849 | 0.830 | 0.778 | 0.516 | 0.319 |
|  | Embryonic Heart Tube Development | 37 | p-value | 0.173 | 0.694 | 0.04** | 0.007*** | 0.804 |  | 0.087* | 0.657 | 0.038** | 0.005*** | 0.989 |  | 0.19 | 0.651 | 0.051* | 0.004*** | 0.698 |
|  |  |  | q-value | 0.850 | 0.952 | 0.743 | 0.334 | 0.426 |  | 0.292 | 0.997 | 0.424 | 0.076 | 1.000 |  | 0.849 | 0.830 | 0.778 | 0.516 | 0.323 |
|  | Exocrine System Development | 26 | p-value | 0.408 | 0.165 | 0.156 | 0.054* | 0.049** |  | 0.187 | 0.207 | 0.036** | 0.009*** | 0.9 |  | 0.467 | 0.149 | 0.16 | 0.06* | 0.065* |
|  |  |  | q-value | 0.850 | 0.952 | 0.743 | 0.337 | 0.351 |  | 0.315 | 0.997 | 0.424 | 0.085 | 1.000 |  | 0.849 | 0.830 | 0.778 | 0.516 | 0.269 |
|  | Blood Vessel Morphogenesis | 216 | p-value | 0.03** | 0.912 | 0.028** | 0.004*** | 0.642 |  | 0.008*** | 0.947 | 0.015** | 0.005*** | 0.467 |  | 0.048** | 0.919 | 0.049** | 0.01** | 0.643 |
|  |  |  | q-value | 0.850 | 0.952 | 0.743 | 0.316 | 0.401 |  | 0.234 | 0.997 | 0.424 | 0.076 | 1.000 |  | 0.849 | 0.833 | 0.778 | 0.516 | 0.317 |
|  | Organ Growth | 37 | p-value | 0.001*** | 0.453 | 0.01** | 0.002*** | 0.675 |  | 0.011** | 0.537 | 0.03** | 0.012** | 0.509 |  | 0.005*** | 0.528 | 0.012** | 0.001*** | 0.679 |
|  |  |  | q-value | 0.850 | 0.952 | 0.743 | 0.257 | 0.404 |  | 0.234 | 0.997 | 0.424 | 0.085 | 1.000 |  | 0.849 | 0.830 | 0.778 | 0.516 | 0.320 |
|  | Olfactory Lobe Development | 17 | p-value | 0.055* | 0.815 | 0.073* | 0.005*** | 0.968 |  | 0.034** | 0.823 | 0.048** | 0.006*** | 0.845 |  | 0.083* | 0.839 | 0.09* | 0.013** | 0.955 |
|  |  |  | q-value | 0.850 | 0.952 | 0.743 | 0.334 | 0.460 |  | 0.266 | 0.997 | 0.424 | 0.081 | 1.000 |  | 0.849 | 0.831 | 0.778 | 0.516 | 0.371 |
|  | Negative Regulation Of Developmental Process | 463 | p-value | 0.188 | 0.426 | 0.084* | 0.017** | 0.944 |  | 0.035** | 0.531 | 0.032** | 0.006*** | 0.937 |  | 0.205 | 0.4 | 0.111 | 0.025** | 0.92 |
|  |  |  | q-value | 0.850 | 0.952 | 0.743 | 0.337 | 0.455 |  | 0.268 | 0.997 | 0.424 | 0.081 | 1.000 |  | 0.849 | 0.830 | 0.778 | 0.516 | 0.363 |
|  | Developmental Process Involved In Reproduction | 343 | p-value | 0.016** | 0.347 | 0.056* | 0.059* | 0.525 |  | 0.011** | 0.312 | 0.02** | 0.037** | 0.876 |  | 0.019** | 0.333 | 0.066* | 0.063* | 0.532 |
|  |  |  | q-value | 0.850 | 0.952 | 0.743 | 0.337 | 0.387 |  | 0.234 | 0.997 | 0.424 | 0.087 | 1.000 |  | 0.849 | 0.830 | 0.778 | 0.516 | 0.302 |
|  | Regulation Of Keratinocyte Differentiation | 18 | p-value | 0.188 | 0.287 | 0.066* | 0.013** | 0.032** |  | 0.015** | 0.42 | 0.006*** | 0*** | 0.632 |  | 0.169 | 0.275 | 0.097* | 0.025** | 0.049** |
|  |  |  | q-value | 0.850 | 0.952 | 0.743 | 0.334 | 0.343 |  | 0.259 | 0.997 | 0.424 | 0.000 | 1.000 |  | 0.849 | 0.830 | 0.778 | 0.516 | 0.269 |
|  | Positive Regulation Of Muscle Tissue Development | 28 | p-value | 0.009*** | 0.395 | 0.024** | 0.011** | 0.129 |  | 0.002*** | 0.423 | 0.006*** | 0.013** | 0.026** |  | 0.011** | 0.395 | 0.038** | 0.016** | 0.172 |
|  |  |  | q-value | 0.850 | 0.952 | 0.743 | 0.334 | 0.352 |  | 0.182 | 0.997 | 0.424 | 0.085 | 1.000 |  | 0.849 | 0.830 | 0.778 | 0.516 | 0.269 |
|  | Positive Regulation Of Dendritic Spine Development | 23 | p-value | 0.94 | 0.136 | 0.055* | 0.068* | 0.025** |  | 0.227 | 0.145 | 0.017** | 0.001*** | 0.72 |  | 0.947 | 0.097* | 0.07* | 0.096* | 0.021** |
|  |  |  | q-value | 0.850 | 0.952 | 0.743 | 0.334 | 0.352 |  | 0.182 | 0.997 | 0.424 | 0.085 | 1.000 |  | 0.849 | 0.830 | 0.778 | 0.516 | 0.269 |
|  | Embryonic Heart Tube Morphogenesis | 28 | p-value | 0.349 | 0.863 | 0.346 | 0.061* | 0.25 |  | 0.019** | 0.914 | 0.025** | 0.007*** | 0.701 |  | 0.327 | 0.856 | 0.409 | 0.09* | 0.215 |
|  |  |  | q-value | 0.850 | 0.952 | 0.743 | 0.337 | 0.361 |  | 0.264 | 0.997 | 0.424 | 0.085 | 1.000 |  | 0.849 | 0.831 | 0.778 | 0.516 | 0.273 |
|  | Positive Regulation Of Neuron Projection Development | 146 | p-value | 0.005*** | 0.92 | 0.006*** | 0*** | 0.344 |  | 0.005*** | 0.976 | 0.02** | 0.009*** | 0.757 |  | 0.012** | 0.956 | 0.006*** | 0.003*** | 0.393 |
|  |  |  | q-value | 0.850 | 0.952 | 0.743 | 0.000 | 0.369 |  | 0.234 | 0.997 | 0.424 | 0.085 | 1.000 |  | 0.849 | 0.835 | 0.778 | 0.516 | 0.289 |
|  | Developmental Growth | 199 | p-value | 0.058* | 0.138 | 0.116 | 0.208 | 0.533 |  | 0.003*** | 0.096* | 0.007*** | 0.041** | 0.139 |  | 0.047** | 0.13 | 0.087* | 0.258 | 0.443 |
|  |  |  | q-value | 0.850 | 0.952 | 0.743 | 0.369 | 0.387 |  | 0.210 | 0.997 | 0.424 | 0.087 | 1.000 |  | 0.849 | 0.830 | 0.778 | 0.517 | 0.293 |
| **localization** | |  |  |  |  |  |  |  |  |  |  |  |  |  |  |  |  |  |  |  |
|  | Regulation Of Telomerase Rna Localization To Cajal Body | 15 | p-value | 0.083* | 0.957 | 0.088* | 0.015** | 0.951 |  | 0.018** | 0.921 | 0.037** | 0.017** | 0.566 |  | 0.091* | 0.927 | 0.094* | 0.022** | 0.922 |
|  |  |  | q-value | 0.850 | 0.953 | 0.743 | 0.337 | 0.457 |  | 0.264 | 0.997 | 0.424 | 0.085 | 1.000 |  | 0.849 | 0.833 | 0.778 | 0.516 | 0.363 |
|  | Regulation Of Leukocyte Migration | 105 | p-value | 0.126 | 0.76 | 0.06* | 0.006*** | 0.315 |  | 0.052* | 0.758 | 0.029** | 0.006*** | 0.991 |  | 0.167 | 0.645 | 0.068* | 0.008*** | 0.341 |
|  |  |  | q-value | 0.850 | 0.952 | 0.743 | 0.334 | 0.368 |  | 0.270 | 0.997 | 0.424 | 0.081 | 1.000 |  | 0.849 | 0.830 | 0.778 | 0.516 | 0.283 |
|  | Ameboidal Type Cell Migration | 86 | p-value | 0.254 | 0.758 | 0.332 | 0.057* | 0.397 |  | 0.009*** | 0.731 | 0.016** | 0.003*** | 0.65 |  | 0.235 | 0.766 | 0.374 | 0.069* | 0.321 |
|  |  |  | q-value | 0.850 | 0.952 | 0.743 | 0.337 | 0.373 |  | 0.234 | 0.997 | 0.424 | 0.076 | 1.000 |  | 0.849 | 0.830 | 0.778 | 0.516 | 0.280 |
|  | Negative Regulation Of Establishment Of Protein Localization | 147 | p-value | 0.002*** | 0.212 | 0.019** | 0.03** | 0.667 |  | 0.002*** | 0.151 | 0.008*** | 0.021** | 0.084* |  | 0.003*** | 0.207 | 0.021** | 0.027** | 0.666 |
|  |  |  | q-value | 0.850 | 0.952 | 0.743 | 0.337 | 0.404 |  | 0.182 | 0.997 | 0.424 | 0.085 | 1.000 |  | 0.849 | 0.830 | 0.778 | 0.516 | 0.319 |
|  | Protein Localization To Chromosome | 36 | p-value | 0.347 | 0.123 | 0.021** | 0.008*** | 0.339 |  | 0.247 | 0.098* | 0.014** | 0.009*** | 0.647 |  | 0.424 | 0.099* | 0.018** | 0.008*** | 0.267 |
|  |  |  | q-value | 0.850 | 0.952 | 0.743 | 0.334 | 0.369 |  | 0.326 | 0.997 | 0.424 | 0.085 | 1.000 |  | 0.849 | 0.830 | 0.778 | 0.516 | 0.280 |
|  | Regulation Of Cellular Extravasation | 17 | p-value | 0.178 | 0.869 | 0.184 | 0.024** | 0.129 |  | 0.006*** | 0.928 | 0.016** | 0.005*** | 0.709 |  | 0.183 | 0.864 | 0.23 | 0.047** | 0.106 |
|  |  |  | q-value | 0.850 | 0.952 | 0.743 | 0.337 | 0.352 |  | 0.234 | 0.997 | 0.424 | 0.076 | 1.000 |  | 0.849 | 0.831 | 0.778 | 0.516 | 0.269 |
|  | Regulation Of Ryanodine Sensitive Calcium Release Channel Activity | 15 | p-value | 0.101 | 0.773 | 0.216 | 0.106 | 0.216 |  | 0.003*** | 0.569 | 0.007*** | 0.022** | 0.292 |  | #N/A | 0.746 | 0.236 | 0.134 | 0.183 |
|  |  |  | q-value | 0.850 | 0.952 | 0.743 | 0.353 | 0.355 |  | 0.210 | 0.997 | 0.424 | 0.085 | 1.000 |  | 0.849 | 0.830 | 0.778 | 0.516 | 0.269 |
|  | Divalent Inorganic Cation Transport | 152 | p-value | 0.177 | 0.78 | 0.2 | 0.025** | 0.367 |  | 0.023** | 0.831 | 0.029** | 0.001*** | 0.924 |  | 0.181 | 0.76 | 0.166 | 0.027** | 0.277 |
|  |  |  | q-value | 0.850 | 0.952 | 0.743 | 0.337 | 0.371 |  | 0.264 | 0.997 | 0.424 | 0.050 | 1.000 |  | 0.849 | 0.830 | 0.778 | 0.516 | 0.280 |
|  | |  |  |  |  |  |  |  |  |  |  |  |  |  |  |  |  |  |  |  |
| **metabolic process** | |  |  |  |  |  |  |  |  |  |  |  |  |  |  |  |  |  |  |  |
|  | Polysaccharide Catabolic Process | 16 | p-value | 0.057* | 0.774 | 0.144 | 0.052* | 0.251 |  | 0.001*** | 0.431 | 0.004*** | 0.009*** | 0.145 |  | 0.051* | 0.633 | 0.125 | 0.068* | 0.237 |
|  |  |  | q-value | 0.850 | 0.952 | 0.743 | 0.337 | 0.361 |  | 0.144 | 0.997 | 0.424 | 0.085 | 1.000 |  | 0.849 | 0.830 | 0.778 | 0.516 | 0.278 |
|  | Cellular Carbohydrate Catabolic Process | 22 | p-value | 0.128 | 0.951 | 0.217 | 0.038** | 0.059* |  | 0*** | 0.899 | 0.002*** | 0.001*** | 0.318 |  | 0.14 | 0.944 | 0.245 | 0.061* | 0.065* |
|  |  |  | q-value | 0.850 | 0.953 | 0.743 | 0.337 | 0.351 |  | 0.000 | 0.997 | 0.424 | 0.050 | 1.000 |  | 0.849 | 0.833 | 0.778 | 0.516 | 0.269 |
|  | Gpi Anchor Metabolic Process | 28 | p-value | 0.09* | 0.898 | 0.123 | 0.032** | 0.646 |  | 0.012** | 0.758 | 0.027** | 0.008*** | 0.356 |  | 0.121 | 0.845 | 0.163 | 0.051* | 0.545 |
|  |  |  | q-value | 0.850 | 0.952 | 0.743 | 0.337 | 0.402 |  | 0.234 | 0.997 | 0.424 | 0.085 | 1.000 |  | 0.849 | 0.831 | 0.778 | 0.516 | 0.304 |
|  | Alcohol Metabolic Process | 218 | p-value | 0.7 | 0.057* | 0.09* | 0.119 | 0.003*** |  | 0.468 | 0.098* | 0.047** | 0.02** | 0.468 |  | 0.696 | 0.052* | 0.103 | 0.178 | 0.003*** |
|  |  |  | q-value | 0.850 | 0.952 | 0.743 | 0.355 | 0.320 |  | 0.367 | 0.997 | 0.424 | 0.085 | 1.000 |  | 0.849 | 0.830 | 0.778 | 0.516 | 0.269 |
|  | Fucosylation | 15 | p-value | 0.176 | 0.606 | 0.062* | 0.002*** | 0.092* |  | 0.018** | 0.787 | 0.008*** | 0.001*** | 0.885 |  | 0.243 | 0.651 | 0.098* | 0.014** | 0.068* |
|  |  |  | q-value | 0.850 | 0.952 | 0.743 | 0.257 | 0.351 |  | 0.264 | 0.997 | 0.424 | 0.050 | 1.000 |  | 0.849 | 0.830 | 0.778 | 0.516 | 0.269 |
|  | Lipid Catabolic Process | 137 | p-value | 0.285 | 0.163 | 0.01** | 0.001*** | 0.937 |  | 0.234 | 0.195 | 0.017** | 0.01** | 0.861 |  | 0.355 | 0.196 | 0.028** | 0.006*** | 0.851 |
|  |  |  | q-value | 0.850 | 0.952 | 0.743 | 0.257 | 0.453 |  | 0.324 | 0.997 | 0.424 | 0.085 | 1.000 |  | 0.849 | 0.830 | 0.778 | 0.516 | 0.350 |
|  | Ubiquitin Dependent Protein Catabolic Process Via The Multivesicular Body Sorting Pathway | 15 | p-value | 0.624 | 0.515 | 0.333 | 0.119 | 0.071* |  | 0.062* | 0.654 | 0.039** | 0.008*** | 0.88 |  | 0.644 | 0.526 | 0.391 | 0.175 | 0.065* |
|  |  |  | q-value | 0.850 | 0.952 | 0.743 | 0.355 | 0.351 |  | 0.280 | 0.997 | 0.424 | 0.085 | 1.000 |  | 0.849 | 0.830 | 0.778 | 0.516 | 0.269 |
|  | Regulation Of Autophasome Assembly | 30 | p-value | 0.189 | 0.162 | 0.243 | 0.4 | 0.339 |  | 0.002*** | 0.075* | 0.006*** | 0.044** | 0.047** |  | 0.146 | 0.12 | 0.182 | 0.468 | 0.278 |
|  |  |  | q-value | 0.850 | 0.952 | 0.743 | 0.410 | 0.369 |  | 0.182 | 0.997 | 0.424 | 0.087 | 1.000 |  | 0.849 | 0.830 | 0.778 | 0.553 | 0.280 |
|  | Protein O Linked Glycosylation | 62 | p-value | 0.512 | 0.572 | 0.371 | 0.097* | 0.125 |  | 0.026** | 0.637 | 0.02** | 0.005*** | 0.703 |  | 0.551 | 0.523 | 0.409 | 0.133 | 0.135 |
|  |  |  | q-value | 0.850 | 0.952 | 0.743 | 0.350 | 0.352 |  | 0.264 | 0.997 | 0.424 | 0.076 | 1.000 |  | 0.849 | 0.830 | 0.778 | 0.516 | 0.269 |
|  | Regulation Of Gluconeogenesis | 31 | p-value | 0.121 | 0.838 | 0.171 | 0.071* | 0.383 |  | 0.024** | 0.821 | 0.049** | 0.011** | 0.42 |  | 0.144 | 0.82 | 0.218 | 0.084* | 0.372 |
|  |  |  | q-value | 0.850 | 0.952 | 0.761 | 0.487 | 0.372 |  | 0.390 | 0.997 | 0.514 | 0.152 | 1.000 |  | 0.849 | 0.830 | 0.793 | 0.589 | 0.297 |
|  | Multicellular Organismal Macromolecule Metabolic Process | 36 | p-value | 0.394 | 0.119 | 0.054* | 0.019** | 0.059* |  | 0.283 | 0.223 | 0.022** | 0.008*** | 0.619 |  | 0.418 | 0.145 | 0.069* | 0.036** | 0.065* |
|  |  |  | q-value | 0.850 | 0.952 | 0.743 | 0.337 | 0.351 |  | 0.332 | 0.997 | 0.424 | 0.085 | 1.000 |  | 0.849 | 0.830 | 0.778 | 0.516 | 0.269 |
|  | |  |  |  |  |  |  |  |  |  |  |  |  |  |  |  |  |  |  |  |
| **immune system process** | |  |  |  |  |  |  |  |  |  |  |  |  |  |  |  |  |  |  |  |
|  | Negative Regulation Of Production Of Molecular Mediator Of Immune Response | 19 | p-value | 0.258 | 0.392 | 0.097* | 0.042** | 0.06* |  | 0.034** | 0.496 | 0.019** | 0.005*** | 0.986 |  | 0.285 | 0.367 | 0.12 | 0.039** | 0.063* |
|  |  |  | q-value | 0.850 | 0.952 | 0.743 | 0.337 | 0.351 |  | 0.266 | 0.997 | 0.424 | 0.076 | 1.000 |  | 0.849 | 0.830 | 0.778 | 0.516 | 0.269 |
|  | Toll Like Receptor Signaling Pathway | 71 | p-value | 0.151 | 0.739 | 0.046** | 0.006*** | 0.082* |  | 0.011** | 0.669 | 0.005*** | 0.002*** | 0.712 |  | 0.183 | 0.687 | 0.074* | 0.013** | 0.089* |
|  |  |  | q-value | 0.850 | 0.952 | 0.743 | 0.334 | 0.351 |  | 0.234 | 0.997 | 0.424 | 0.065 | 1.000 |  | 0.849 | 0.830 | 0.778 | 0.516 | 0.269 |
|  | Regulation Of Megakaryocyte Differentiation | 20 | p-value | 0.07* | 0.825 | 0.087* | 0.022** | 0.536 |  | 0.012** | 0.762 | 0.023** | 0.006*** | 0.352 |  | 0.087* | 0.802 | 0.129 | 0.043** | 0.425 |
|  |  |  | q-value | 0.850 | 0.952 | 0.743 | 0.337 | 0.387 |  | 0.234 | 0.997 | 0.424 | 0.081 | 1.000 |  | 0.849 | 0.830 | 0.778 | 0.516 | 0.292 |
|  | Positive T Cell Selection | 17 | p-value | 0.264 | 0.572 | 0.092* | 0.009*** | 0.2 |  | 0.019** | 0.739 | 0.011** | 0.002*** | 0.924 |  | 0.274 | 0.653 | 0.117 | 0.024** | 0.176 |
|  |  |  | q-value | 0.850 | 0.952 | 0.743 | 0.334 | 0.355 |  | 0.264 | 0.997 | 0.424 | 0.065 | 1.000 |  | 0.849 | 0.830 | 0.778 | 0.516 | 0.269 |
|  | Negative Regulation Of Myeloid Leukocyte Differentiation | 30 | p-value | 0.447 | 0.215 | 0.067* | 0.018** | 0.301 |  | 0.121 | 0.349 | 0.022** | 0.009*** | 0.904 |  | 0.467 | 0.208 | 0.079* | 0.042** | 0.263 |
|  |  |  | q-value | 0.850 | 0.952 | 0.743 | 0.337 | 0.368 |  | 0.302 | 0.997 | 0.424 | 0.085 | 1.000 |  | 0.849 | 0.830 | 0.778 | 0.516 | 0.279 |
|  | Osteoclast Differentiation | 20 | p-value | 0.739 | 0.126 | 0.109 | 0.044** | 0.026** |  | 0.083* | 0.271 | 0.017** | 0.004*** | 0.898 |  | 0.768 | 0.171 | 0.142 | 0.088* | 0.023** |
|  |  |  | q-value | 0.850 | 0.952 | 0.743 | 0.337 | 0.343 |  | 0.289 | 0.997 | 0.424 | 0.076 | 1.000 |  | 0.849 | 0.830 | 0.778 | 0.516 | 0.269 |
|  | Negative Regulation Of Osteoclast Differentiation | 16 | p-value | 0.11 | 0.547 | 0.053* | 0.007*** | 0.718 |  | 0.037** | 0.69 | 0.021** | 0.008*** | 0.782 |  | 0.111 | 0.575 | 0.063* | 0.014** | 0.544 |
|  |  |  | q-value | 0.850 | 0.952 | 0.743 | 0.334 | 0.411 |  | 0.268 | 0.997 | 0.424 | 0.085 | 1.000 |  | 0.849 | 0.830 | 0.778 | 0.516 | 0.304 |
|  | Regulation Of Production Of Molecular Mediator Of Immune Response | 70 | p-value | 0.001*** | 0.396 | 0.009*** | 0.012** | 0.868 |  | 0*** | 0.339 | 0.004*** | 0.023** | 0.211 |  | 0.001*** | 0.374 | 0.008*** | 0.012** | 0.942 |
|  |  |  | q-value | 0.850 | 0.952 | 0.743 | 0.334 | 0.439 |  | 0.000 | 0.997 | 0.424 | 0.085 | 1.000 |  | 0.849 | 0.830 | 0.778 | 0.516 | 0.367 |
|  | Regulation Of B Cell Differentiation | 18 | p-value | 0.236 | 0.552 | 0.119 | 0.013** | 0.356 |  | 0.057* | 0.688 | 0.046** | 0.013** | 0.868 |  | 0.257 | 0.622 | 0.136 | 0.035** | 0.285 |
|  |  |  | q-value | 0.850 | 0.952 | 0.743 | 0.334 | 0.370 |  | 0.275 | 0.997 | 0.424 | 0.085 | 1.000 |  | 0.849 | 0.830 | 0.778 | 0.516 | 0.280 |
|  | |  |  |  |  |  |  |  |  |  |  |  |  |  |  |  |  |  |  |  |
| **response to stimulus** | |  |  |  |  |  |  |  |  |  |  |  |  |  |  |  |  |  |  |  |
|  | Response To Acid Chemical | 200 | p-value | 0.323 | 0.999 | 0.485 | 0.214 | 0.079* |  | 0.014** | 0.966 | 0.023** | 0.014** | 0.226 |  | 0.327 | 0.998 | 0.51 | 0.207 | 0.102 |
|  |  |  | q-value | 0.850 | 0.960 | 0.743 | 0.369 | 0.351 |  | 0.248 | 0.997 | 0.424 | 0.085 | 1.000 |  | 0.849 | 0.845 | 0.778 | 0.517 | 0.269 |
|  | Regulation Of Intracellular Estrogen Receptor Signaling Pathway | 20 | p-value | 0.072* | 0.991 | 0.059* | 0.013** | 0.322 |  | 0.012** | 0.999 | 0.017** | 0.004*** | 0.507 |  | 0.096* | 0.974 | 0.083* | 0.014** | 0.314 |
|  |  |  | q-value | 0.850 | 0.952 | 0.743 | 0.408 | 0.351 |  | 0.369 | 0.997 | 0.492 | 0.165 | 1.000 |  | 0.849 | 0.830 | 0.778 | 0.546 | 0.269 |
|  | Response To Camp | 62 | p-value | 0.021** | 0.536 | 0.052* | 0.044** | 0.559 |  | 0.001*** | 0.39 | 0.01** | 0.02** | 0.334 |  | 0.018** | 0.488 | 0.059* | 0.053* | 0.501 |
|  |  |  | q-value | 0.850 | 0.952 | 0.743 | 0.337 | 0.391 |  | 0.144 | 0.997 | 0.424 | 0.085 | 1.000 |  | 0.849 | 0.830 | 0.778 | 0.516 | 0.299 |
|  | Detection Of Biotic Stimulus | 18 | p-value | 0.691 | 0.2 | 0.16 | 0.097* | 0.018** |  | 0.128 | 0.327 | 0.02** | 0.005*** | 0.712 |  | 0.644 | 0.221 | 0.234 | 0.112 | 0.016** |
|  |  |  | q-value | 0.850 | 0.952 | 0.743 | 0.350 | 0.343 |  | 0.303 | 0.997 | 0.424 | 0.076 | 1.000 |  | 0.849 | 0.830 | 0.778 | 0.516 | 0.269 |
|  | Detection Of Stimulus | 141 | p-value | 0.136 | 0.845 | 0.31 | 0.166 | 0.363 |  | 0.002*** | 0.771 | 0.015** | 0.016** | 0.648 |  | 0.166 | 0.866 | 0.334 | 0.206 | 0.389 |
|  |  |  | q-value | 0.850 | 0.952 | 0.743 | 0.362 | 0.371 |  | 0.182 | 0.997 | 0.424 | 0.085 | 1.000 |  | 0.849 | 0.831 | 0.778 | 0.517 | 0.288 |
|  | Response To Gonadotropin | 17 | p-value | 0.091* | 0.508 | 0.006*** | 0.001*** | 0.286 |  | 0.01** | 0.615 | 0.001*** | 0*** | 0.965 |  | 0.136 | 0.524 | 0.015** | 0.002*** | 0.222 |
|  |  |  | q-value | 0.850 | 0.952 | 0.743 | 0.257 | 0.366 |  | 0.234 | 0.997 | 0.267 | 0.000 | 1.000 |  | 0.849 | 0.830 | 0.778 | 0.516 | 0.276 |
|  | Regulation Of Camp Metabolic Process | 69 | p-value | 0.082* | 0.556 | 0.24 | 0.126 | 0.397 |  | 0.007*** | 0.33 | 0.022** | 0.036** | 0.099* |  | 0.072* | 0.503 | 0.202 | 0.116 | 0.414 |
|  |  |  | q-value | 0.850 | 0.952 | 0.743 | 0.355 | 0.373 |  | 0.234 | 0.997 | 0.424 | 0.087 | 1.000 |  | 0.849 | 0.830 | 0.778 | 0.516 | 0.291 |
|  | Cellular Defense Response | 45 | p-value | 0.101 | 0.902 | 0.098* | 0.012** | 0.595 |  | 0.032** | 0.948 | 0.043** | 0.016** | 0.631 |  | 0.121 | 0.893 | 0.125 | 0.026** | 0.492 |
|  |  |  | q-value | 0.850 | 0.952 | 0.743 | 0.334 | 0.395 |  | 0.264 | 0.997 | 0.424 | 0.085 | 1.000 |  | 0.849 | 0.831 | 0.778 | 0.516 | 0.298 |
|  | Positive Chemotaxis | 15 | p-value | 0.116 | 0.262 | 0.015** | 0.001*** | 0.475 |  | 0.211 | 0.218 | 0.028** | 0.013** | 0.893 |  | 0.161 | 0.227 | 0.025** | 0.006*** | 0.468 |
|  |  |  | q-value | 0.850 | 0.952 | 0.743 | 0.257 | 0.383 |  | 0.321 | 0.997 | 0.424 | 0.085 | 1.000 |  | 0.849 | 0.830 | 0.778 | 0.516 | 0.297 |
|  | |  |  |  |  |  |  |  |  |  |  |  |  |  |  |  |  |  |  |  |
| **cellular process** | |  |  |  |  |  |  |  |  |  |  |  |  |  |  |  |  |  |  |  |
|  | |  |  |  |  |  |  |  |  |  |  |  |  |  |  |  |  |  |  |  |
| **cellular process: cell communication: cell-cell signaling** | |  |  |  |  |  |  |  |  |  |  |  |  |  |  |  |  |  |  |  |
|  | Non Canonical Wnt Signaling Pathway | 104 | p-value | 0.022** | 0.603 | 0.052* | 0.04** | 0.278 |  | 0.003*** | 0.592 | 0.022** | 0.018** | 0.126 |  | 0.034** | 0.638 | 0.09* | 0.055* | 0.284 |
|  |  |  | q-value | 0.850 | 0.952 | 0.743 | 0.337 | 0.364 |  | 0.210 | 0.997 | 0.424 | 0.085 | 1.000 |  | 0.849 | 0.830 | 0.778 | 0.516 | 0.280 |
|  | Excitatory Postsynaptic Potential | 15 | p-value | 0.95 | 0.307 | 0.198 | 0.155 | 0.059* |  | 0.236 | 0.434 | 0.041** | 0.014** | 0.936 |  | 0.968 | 0.257 | 0.234 | 0.198 | 0.044** |
|  |  |  | q-value | 0.856 | 0.952 | 0.743 | 0.359 | 0.351 |  | 0.324 | 0.997 | 0.424 | 0.085 | 1.000 |  | 0.858 | 0.830 | 0.778 | 0.517 | 0.269 |
|  | Canonical Wnt Signaling Pathway | 55 | p-value | 0.043** | 0.763 | 0.039** | 0.002*** | 0.381 |  | 0.027** | 0.817 | 0.049** | 0.011** | 0.741 |  | 0.052* | 0.754 | 0.052* | 0.017** | 0.395 |
|  |  |  | q-value | 0.850 | 0.952 | 0.743 | 0.257 | 0.372 |  | 0.264 | 0.997 | 0.424 | 0.085 | 1.000 |  | 0.849 | 0.830 | 0.778 | 0.516 | 0.289 |
|  | Signal Release | 98 | p-value | 0.622 | 0.246 | 0.094* | 0.04** | 0.115 |  | 0.069* | 0.489 | 0.024** | 0.004*** | 0.914 |  | 0.625 | 0.324 | 0.145 | 0.06* | 0.096* |
|  |  |  | q-value | 0.850 | 0.952 | 0.743 | 0.337 | 0.352 |  | 0.281 | 0.997 | 0.424 | 0.076 | 1.000 |  | 0.849 | 0.830 | 0.778 | 0.516 | 0.269 |
|  | |  |  |  |  |  |  |  |  |  |  |  |  |  |  |  |  |  |  |  |
| **cellular process: cell communication: signal transduction** | |  |  |  |  |  |  |  |  |  |  |  |  |  |  |  |  |  |  |  |
|  | Platelet Derived Growth Factor Receptor Signaling Pathway | 25 | p-value | 0.135 | 0.955 | 0.194 | 0.043** | 0.161 |  | 0.011** | 0.927 | 0.021** | 0.007*** | 0.372 |  | 0.123 | 0.956 | 0.211 | 0.044** | 0.161 |
|  |  |  | q-value | 0.850 | 0.952 | 0.759 | 0.417 | 0.355 |  | 0.392 | 0.997 | 0.480 | 0.127 | 1.000 |  | 0.849 | 0.830 | 0.794 | 0.546 | 0.269 |
|  | Negative Regulation Of Erk1 And Erk2 Cascade | 39 | p-value | 0.276 | 0.473 | 0.492 | 0.437 | 0.136 |  | 0.013** | 0.305 | 0.038** | 0.041** | 0.284 |  | 0.242 | 0.448 | 0.435 | 0.425 | 0.142 |
|  |  |  | q-value | 0.850 | 0.952 | 0.743 | 0.417 | 0.355 |  | 0.237 | 0.997 | 0.424 | 0.087 | 1.000 |  | 0.849 | 0.830 | 0.778 | 0.545 | 0.269 |
|  | Negative Regulation Of Signal Transduction In Absence Of Ligand | 22 | p-value | 0.46 | 0.448 | 0.245 | 0.091* | 0.054* |  | 0.034** | 0.734 | 0.033** | 0.008*** | 0.665 |  | 0.397 | 0.481 | 0.298 | 0.112 | 0.05* |
|  |  |  | q-value | 0.850 | 0.952 | 0.743 | 0.346 | 0.351 |  | 0.266 | 0.997 | 0.424 | 0.085 | 1.000 |  | 0.849 | 0.830 | 0.778 | 0.516 | 0.269 |
|  | Positive Regulation Of Erk1 And Erk2 Cascade | 106 | p-value | 0.055* | 0.852 | 0.062* | 0.01** | 0.159 |  | 0.001*** | 0.764 | 0*** | 0.002*** | 0.416 |  | 0.064* | 0.818 | 0.088* | 0.026** | 0.136 |
|  |  |  | q-value | 0.850 | 0.952 | 0.743 | 0.334 | 0.355 |  | 0.144 | 0.997 | 0.000 | 0.065 | 1.000 |  | 0.849 | 0.830 | 0.778 | 0.516 | 0.269 |
|  | |  |  |  |  |  |  |  |  |  |  |  |  |  |  |  |  |  |  |  |
| **cellular process: cellular metabolic process** | |  |  |  |  |  |  |  |  |  |  |  |  |  |  |  |  |  |  |  |
|  | Phospholipid Dephosphorylation | 22 | p-value | 0.031** | 0.652 | 0.062* | 0.029** | 0.206 |  | 0.008*** | 0.564 | 0.024** | 0.013** | 0.197 |  | 0.032** | 0.603 | 0.085* | 0.033** | 0.21 |
|  |  |  | q-value | 0.850 | 0.952 | 0.743 | 0.337 | 0.355 |  | 0.234 | 0.997 | 0.424 | 0.085 | 1.000 |  | 0.849 | 0.830 | 0.778 | 0.516 | 0.273 |
|  | Glutamate Metabolic Process | 20 | p-value | 0.125 | 0.911 | 0.287 | 0.141 | 0.228 |  | 0.015** | 0.76 | 0.048** | 0.033** | 0.276 |  | 0.118 | 0.907 | 0.284 | 0.114 | 0.209 |
|  |  |  | q-value | 0.850 | 0.952 | 0.743 | 0.355 | 0.358 |  | 0.259 | 0.997 | 0.424 | 0.087 | 1.000 |  | 0.849 | 0.832 | 0.778 | 0.516 | 0.273 |
|  | Ribonucleoside Triphosphate Biosynthetic Process | 42 | p-value | 0.212 | 0.223 | 0.367 | 0.375 | 0.164 |  | 0.01** | 0.173 | 0.035** | 0.04** | 0.172 |  | 0.209 | 0.206 | 0.283 | 0.417 | 0.153 |
|  |  |  | q-value | 0.850 | 0.952 | 0.743 | 0.403 | 0.355 |  | 0.234 | 0.997 | 0.424 | 0.087 | 1.000 |  | 0.849 | 0.830 | 0.778 | 0.544 | 0.269 |
|  | Positive Regulation Of Phosphorus Metabolic Process | 659 | p-value | 0.15 | 0.334 | 0.028** | 0.002*** | 0.335 |  | 0.067* | 0.426 | 0.024** | 0.003*** | 0.244 |  | 0.165 | 0.389 | 0.049** | 0.006*** | 0.369 |
|  |  |  | q-value | 0.850 | 0.952 | 0.743 | 0.257 | 0.369 |  | 0.281 | 0.997 | 0.424 | 0.076 | 1.000 |  | 0.849 | 0.830 | 0.778 | 0.516 | 0.285 |
|  | Telomere Maintenance Via Recombination | 25 | p-value | 0.024** | 0.329 | 0.067* | 0.021** | 0.04** |  | 0.002*** | 0.423 | 0.003*** | 0.001*** | 0.634 |  | 0.043** | 0.359 | 0.128 | 0.033** | 0.036** |
|  |  |  | q-value | 0.850 | 0.952 | 0.743 | 0.337 | 0.351 |  | 0.182 | 0.997 | 0.424 | 0.050 | 1.000 |  | 0.849 | 0.830 | 0.778 | 0.516 | 0.269 |
|  | Positive Regulation Of Nucleotide Metabolic Process | 73 | p-value | 0.063* | 0.84 | 0.085* | 0.012** | 0.501 |  | 0.002*** | 0.718 | 0.009*** | 0.004*** | 0.582 |  | 0.071* | 0.777 | 0.091* | 0.015** | 0.49 |
|  |  |  | q-value | 0.850 | 0.952 | 0.743 | 0.334 | 0.385 |  | 0.182 | 0.997 | 0.424 | 0.076 | 1.000 |  | 0.849 | 0.830 | 0.778 | 0.516 | 0.298 |
|  | Negative Regulation Of Dephosphorylation | 49 | p-value | 0.637 | 0.182 | 0.134 | 0.097* | 0.037** |  | 0.17 | 0.243 | 0.048** | 0.017** | 0.799 |  | 0.674 | 0.152 | 0.156 | 0.128 | 0.042** |
|  |  |  | q-value | 0.850 | 0.952 | 0.743 | 0.350 | 0.347 |  | 0.314 | 0.997 | 0.424 | 0.085 | 1.000 |  | 0.849 | 0.830 | 0.778 | 0.516 | 0.269 |
|  | Mrna Transcription | 16 | p-value | 0.056* | 0.215 | 0.146 | 0.212 | 0.342 |  | 0.001*** | 0.151 | 0.007*** | 0.023** | 0.137 |  | 0.066* | 0.184 | 0.131 | 0.242 | 0.324 |
|  |  |  | q-value | 0.850 | 0.952 | 0.743 | 0.369 | 0.369 |  | 0.144 | 0.997 | 0.424 | 0.085 | 1.000 |  | 0.849 | 0.830 | 0.778 | 0.517 | 0.281 |
|  | Positive Regulation Of Dephosphorylation | 33 | p-value | 0.389 | 0.555 | 0.318 | 0.091* | 0.136 |  | 0.046** | 0.918 | 0.049** | 0.014** | 0.674 |  | 0.356 | 0.657 | 0.299 | 0.092* | 0.146 |
|  |  |  | q-value | 0.850 | 0.952 | 0.743 | 0.346 | 0.355 |  | 0.268 | 0.997 | 0.424 | 0.085 | 1.000 |  | 0.849 | 0.830 | 0.778 | 0.516 | 0.269 |
|  | |  |  | |  |  |  |  |  |  |  |  |  |  |  |  |  |  |  |  |
| **cellular process: cellular metabolic process: cellular macromolecule metabolic process: cellular protein metabolic process** | |  |  | |  |  |  |  |  |  |  |  |  |  |  |  |  |  |  |  |
|  | Histone Methylation | 64 | p-value | 0.157 | 0.94 | 0.131 | 0.017** | 0.663 |  | 0.029** | 0.968 | 0.037** | 0.001*** | 0.94 |  | 0.159 | 0.957 | 0.163 | 0.028** | 0.604 |
|  |  |  | q-value | 0.850 | 0.952 | 0.743 | 0.337 | 0.403 |  | 0.264 | 0.997 | 0.424 | 0.050 | 1.000 |  | 0.849 | 0.835 | 0.778 | 0.516 | 0.311 |
|  | Histone Monoubiquitination | 20 | p-value | 0.722 | 0.243 | 0.109 | 0.047** | 0.21 |  | 0.272 | 0.326 | 0.036** | 0.011** | 0.638 |  | 0.774 | 0.279 | 0.173 | 0.103 | 0.148 |
|  |  |  | q-value | 0.850 | 0.952 | 0.743 | 0.337 | 0.355 |  | 0.328 | 0.997 | 0.424 | 0.085 | 1.000 |  | 0.849 | 0.830 | 0.778 | 0.516 | 0.269 |
|  | Protein Polyubiquitination | 204 | p-value | 0.4 | 0.786 | 0.238 | 0.049** | 0.099* |  | 0.026** | 0.942 | 0.009*** | 0.002*** | 0.941 |  | 0.411 | 0.789 | 0.292 | 0.066* | 0.088* |
|  |  |  | q-value | 0.850 | 0.952 | 0.743 | 0.337 | 0.351 |  | 0.264 | 0.997 | 0.424 | 0.065 | 1.000 |  | 0.849 | 0.830 | 0.778 | 0.516 | 0.269 |
|  | Peptidyl Glutamic Acid Modification | 16 | p-value | 0.022** | 0.9 | 0.044** | 0.013** | 0.351 |  | 0*** | 0.804 | 0.003*** | 0*** | 0.381 |  | 0.029** | 0.908 | 0.048** | 0.012** | 0.304 |
|  |  |  | q-value | 0.850 | 0.952 | 0.743 | 0.334 | 0.369 |  | 0.000 | 0.997 | 0.424 | 0.000 | 1.000 |  | 0.849 | 0.832 | 0.778 | 0.516 | 0.280 |
|  | Protein Dephosphorylation | 138 | p-value | 0.031** | 0.735 | 0.088* | 0.047** | 0.907 |  | 0.003*** | 0.588 | 0.011** | 0.016** | 0.096* |  | 0.032** | 0.721 | 0.086* | 0.04** | 0.874 |
|  |  |  | q-value | 0.850 | 0.952 | 0.743 | 0.337 | 0.448 |  | 0.210 | 0.997 | 0.424 | 0.085 | 1.000 |  | 0.849 | 0.830 | 0.778 | 0.516 | 0.354 |
|  | |  |  | |  |  |  |  |  |  |  |  |  |  |  |  |  |  |  |  |
| **cellular process: cellular response to stimulus** | |  |  | |  |  |  |  |  |  |  |  |  |  |  |  |  |  |  |  |
|  | Intrinsic Apoptotic Signaling Pathway In Response To Dna Damage | 56 | p-value | 0.441 | 0.214 | 0.109 | 0.036** | 0.101 |  | 0.08* | 0.29 | 0.029** | 0.004*** | 0.601 |  | 0.514 | 0.204 | 0.149 | 0.072* | 0.066* |
|  |  |  | q-value | 0.850 | 0.952 | 0.743 | 0.337 | 0.351 |  | 0.287 | 0.997 | 0.424 | 0.076 | 1.000 |  | 0.849 | 0.830 | 0.778 | 0.516 | 0.269 |
|  | Nucleotide Excision Repair | 103 | p-value | 0.696 | 0.045** | 0.055* | 0.119 | 0.073* |  | 0.549 | 0.076* | 0.037** | 0.036** | 0.417 |  | 0.653 | 0.033** | 0.063* | 0.155 | 0.065* |
|  |  |  | q-value | 0.850 | 0.952 | 0.743 | 0.355 | 0.351 |  | 0.379 | 0.997 | 0.424 | 0.087 | 1.000 |  | 0.849 | 0.830 | 0.778 | 0.516 | 0.269 |
|  | Positive Regulation Of Dna Repair | 29 | p-value | 0.316 | 0.171 | 0.053* | 0.013** | 0.177 |  | 0.127 | 0.303 | 0.021** | 0.008*** | 0.986 |  | 0.313 | 0.181 | 0.062* | 0.027** | 0.158 |
|  |  |  | q-value | 0.850 | 0.952 | 0.743 | 0.334 | 0.355 |  | 0.303 | 0.997 | 0.424 | 0.085 | 1.000 |  | 0.849 | 0.830 | 0.778 | 0.516 | 0.269 |
|  | Regulation Of Response To Reactive Oxygen Species | 28 | p-value | 0.667 | 0.059* | 0.016** | 0.023** | 0.23 |  | 0.357 | 0.126 | 0.036** | 0.015** | 0.49 |  | 0.698 | 0.078* | 0.024** | 0.028** | 0.162 |
|  |  |  | q-value | 0.850 | 0.952 | 0.743 | 0.337 | 0.358 |  | 0.344 | 0.997 | 0.424 | 0.085 | 1.000 |  | 0.849 | 0.830 | 0.778 | 0.516 | 0.269 |
|  | Cellular Response To Amino Acid Stimulus | 37 | p-value | 0.72 | 0.065* | 0.063* | 0.068* | 0.052* |  | 0.226 | 0.123 | 0.029** | 0.014** | 0.601 |  | 0.711 | 0.071* | 0.09* | 0.11 | 0.048** |
|  |  |  | q-value | 0.850 | 0.952 | 0.743 | 0.342 | 0.351 |  | 0.324 | 0.997 | 0.424 | 0.085 | 1.000 |  | 0.849 | 0.830 | 0.778 | 0.516 | 0.269 |
|  | |  |  | |  |  |  |  |  |  |  |  |  |  |  |  |  |  |  |  |
| **cellular process: cell differentiation** | |  |  | |  |  |  |  |  |  |  |  |  |  |  |  |  |  |  |  |
|  | Positive Regulation Of Fat Cell Differentiation | 27 | p-value | 0.013** | 0.702 | 0.045** | 0.013** | 0.087* |  | 0.019** | 0.737 | 0.042** | 0.02** | 0.415 |  | 0.027** | 0.715 | 0.061* | 0.017** | 0.095* |
|  |  |  | q-value | 0.850 | 0.952 | 0.743 | 0.334 | 0.351 |  | 0.264 | 0.997 | 0.424 | 0.085 | 1.000 |  | 0.849 | 0.830 | 0.778 | 0.516 | 0.269 |
|  | Fat Cell Differentiation | 74 | p-value | 0.062* | 0.561 | 0.165 | 0.143 | 0.589 |  | 0.008*** | 0.475 | 0.033** | 0.035** | 0.187 |  | 0.066* | 0.585 | 0.194 | 0.143 | 0.581 |
|  |  |  | q-value | 0.850 | 0.952 | 0.743 | 0.356 | 0.394 |  | 0.234 | 0.997 | 0.424 | 0.087 | 1.000 |  | 0.849 | 0.830 | 0.778 | 0.516 | 0.308 |
|  | |  |  | |  |  |  |  |  |  |  |  |  |  |  |  |  |  |  |  |
| **cellular process: cell activation** | |  |  | |  |  |  |  |  |  |  |  |  |  |  |  |  |  |  |  |
|  | B Cell Activation | 93 | p-value | 0.019** | 0.941 | 0.024** | 0.002*** | 0.656 |  | 0.012** | 0.897 | 0.031** | 0.024** | 0.847 |  | 0.025** | 0.877 | 0.022** | 0.008*** | 0.688 |
|  |  |  | q-value | 0.850 | 0.952 | 0.743 | 0.257 | 0.402 |  | 0.234 | 0.997 | 0.424 | 0.086 | 1.000 |  | 0.849 | 0.831 | 0.778 | 0.516 | 0.321 |
|  | Lipoprotein Biosynthetic Process | 68 | p-value | 0.049** | 0.805 | 0.068* | 0.005*** | 0.755 |  | 0.014** | 0.819 | 0.02** | 0.009*** | 0.46 |  | 0.054* | 0.782 | 0.073* | 0.009*** | 0.739 |
|  |  |  | q-value | 0.850 | 0.952 | 0.743 | 0.334 | 0.419 |  | 0.248 | 0.997 | 0.424 | 0.085 | 1.000 |  | 0.849 | 0.830 | 0.778 | 0.516 | 0.329 |
|  | |  |  | |  |  |  |  |  |  |  |  |  |  |  |  |  |  |  |  |
| **cellular process: cell cycle** | |  |  | |  |  |  |  |  |  |  |  |  |  |  |  |  |  |  |  |
|  | Negative Regulation Of Mitotic Nuclear Division | 26 | p-value | 0.439 | 0.634 | 0.216 | 0.052* | 0.078* |  | 0.038** | 0.883 | 0.034** | 0.002*** | 0.737 |  | 0.446 | 0.703 | 0.242 | 0.073* | 0.067* |
|  |  |  | q-value | 0.850 | 0.952 | 0.743 | 0.337 | 0.351 |  | 0.268 | 0.997 | 0.424 | 0.065 | 1.000 |  | 0.849 | 0.830 | 0.778 | 0.516 | 0.269 |
|  | Regulation Of Cell Cycle Phase Transition | 261 | p-value | 0.185 | 0.32 | 0.087* | 0.007*** | 0.235 |  | 0.043** | 0.36 | 0.027** | 0.008*** | 0.531 |  | 0.217 | 0.291 | 0.095* | 0.009*** | 0.235 |
|  |  |  | q-value | 0.850 | 0.952 | 0.743 | 0.334 | 0.358 |  | 0.268 | 0.997 | 0.424 | 0.085 | 1.000 |  | 0.849 | 0.830 | 0.778 | 0.516 | 0.278 |
|  | |  |  | |  |  |  |  |  |  |  |  |  |  |  |  |  |  |  |  |
| **cellular process: others** | |  |  | |  |  |  |  |  |  |  |  |  |  |  |  |  |  |  |  |
|  | Chaperone Mediated Protein Folding | 40 | p-value | 0.019** | 0.437 | 0.065* | 0.051* | 0.636 |  | 0.004*** | 0.417 | 0.035** | 0.045** | 0.185 |  | 0.02** | 0.421 | 0.061* | 0.048** | 0.592 |
|  |  |  | q-value | 0.850 | 0.952 | 0.743 | 0.337 | 0.399 |  | 0.223 | 0.997 | 0.424 | 0.087 | 1.000 |  | 0.849 | 0.830 | 0.778 | 0.516 | 0.309 |
|  | Positive Regulation Of Protein Oligomerization | 15 | p-value | 0.149 | 0.799 | 0.272 | 0.068* | 0.439 |  | 0.009*** | 0.653 | 0.031** | 0.013** | 0.402 |  | 0.137 | 0.785 | 0.232 | 0.092* | 0.443 |
|  |  |  | q-value | 0.850 | 0.952 | 0.743 | 0.342 | 0.378 |  | 0.234 | 0.997 | 0.424 | 0.085 | 1.000 |  | 0.849 | 0.830 | 0.778 | 0.516 | 0.293 |
|  | |  |  |  |  |  |  |  |  |  |  |  |  |  |  |  |  |  |  |  |
| **cellular component organization or biogenesis** | |  |  |  |  |  |  |  |  |  |  |  |  |  |  |  |  |  |  |  |
|  | Regulation Of Cell Size | 117 | p-value | 0.849 | 0.071* | 0.054* | 0.071* | 0.188 |  | 0.431 | #N/A | 0.037** | 0.026** | 0.669 |  | 0.889 | 0.071* | 0.072* | 0.092* | 0.118 |
|  |  |  | q-value | 0.850 | 0.952 | 0.743 | 0.342 | 0.355 |  | 0.360 | 0.997 | 0.424 | 0.086 | 1.000 |  | 0.851 | 0.830 | 0.778 | 0.516 | 0.269 |
|  | Membrane Invagination | 24 | p-value | 0.687 | 0.103 | 0.044** | 0.032** | 0.237 |  | 0.26 | 0.16 | 0.026** | 0.016** | 0.692 |  | 0.721 | 0.117 | 0.055* | 0.051* | 0.204 |
|  |  |  | q-value | 0.850 | 0.952 | 0.743 | 0.337 | 0.358 |  | 0.328 | 0.997 | 0.424 | 0.085 | 1.000 |  | 0.849 | 0.830 | 0.778 | 0.516 | 0.272 |
|  | Positive Regulation Of Protein Polymerization | 64 | p-value | 0.427 | 0.693 | 0.373 | 0.088* | 0.174 |  | 0.024** | 0.746 | 0.035** | 0.005*** | 0.754 |  | 0.441 | 0.702 | 0.391 | 0.131 | 0.159 |
|  |  |  | q-value | 0.850 | 0.952 | 0.743 | 0.344 | 0.355 |  | 0.264 | 0.997 | 0.424 | 0.076 | 1.000 |  | 0.849 | 0.830 | 0.778 | 0.516 | 0.269 |
|  | Lamellipodium Assembly | 21 | p-value | 0.017** | 0.815 | 0.021** | 0.001*** | 0.801 |  | 0.017** | 0.735 | 0.031** | 0.001*** | 0.655 |  | 0.028** | 0.754 | 0.026** | 0.001*** | 0.704 |
|  |  |  | q-value | 0.850 | 0.952 | 0.743 | 0.257 | 0.425 |  | 0.262 | 0.997 | 0.424 | 0.050 | 1.000 |  | 0.849 | 0.830 | 0.778 | 0.516 | 0.325 |
|  | |  |  |  |  |  |  |  |  |  |  |  |  |  |  |  |  |  |  |  |
| **biological regulation** | |  |  |  |  |  |  |  |  |  |  |  |  |  |  |  |  |  |  |  |
|  | Regulation Of Monooxygenase Activity | 41 | p-value | 0.186 | 0.488 | 0.102 | 0.019** | 0.391 |  | 0.029** | 0.59 | 0.021** | 0.007*** | 0.676 |  | 0.176 | 0.509 | 0.125 | 0.032** | 0.38 |
|  |  |  | q-value | 0.850 | 0.952 | 0.743 | 0.337 | 0.373 |  | 0.264 | 0.997 | 0.424 | 0.085 | 1.000 |  | 0.849 | 0.830 | 0.778 | 0.516 | 0.287 |
|  | Negative Regulation Of Nf Kappab Transcription Factor Activity | 47 | p-value | 0.03** | 0.888 | 0.041** | 0.004*** | 0.985 |  | 0.008*** | 0.81 | 0.03** | 0.004*** | 0.379 |  | 0.032** | 0.841 | 0.069* | 0.011** | 0.971 |
|  |  |  | q-value | 0.850 | 0.952 | 0.743 | 0.316 | 0.464 |  | 0.234 | 0.997 | 0.424 | 0.076 | 1.000 |  | 0.849 | 0.831 | 0.778 | 0.516 | 0.374 |
|  | Endoplasmic Reticulum Calcium Ion Homeostasis | 17 | p-value | 0.14 | 0.892 | 0.138 | 0.024** | 0.449 |  | 0.012** | 0.879 | 0.021** | 0.005*** | 0.56 |  | 0.159 | 0.862 | 0.169 | 0.048** | 0.459 |
|  |  |  | q-value | 0.850 | 0.952 | 0.743 | 0.337 | 0.379 |  | 0.234 | 0.997 | 0.424 | 0.076 | 1.000 |  | 0.849 | 0.831 | 0.778 | 0.516 | 0.295 |
|  | Regulation Of Heart Rate By Cardiac Conduction | 15 | p-value | 0.135 | 0.867 | 0.113 | 0.026** | 0.117 |  | 0.012** | 0.972 | 0.023** | 0.002*** | 0.874 |  | 0.145 | 0.879 | 0.154 | 0.044** | 0.12 |
|  |  |  | q-value | 0.850 | 0.952 | 0.743 | 0.337 | 0.352 |  | 0.234 | 0.997 | 0.424 | 0.065 | 1.000 |  | 0.849 | 0.831 | 0.778 | 0.516 | 0.269 |
|  | Cardiac Muscle Cell Action Potential | 17 | p-value | 0.084* | 0.742 | 0.057* | 0.01** | 0.339 |  | 0.015** | 0.712 | 0.021** | 0.003*** | 0.508 |  | 0.091* | 0.722 | 0.082* | 0.015** | 0.382 |
|  |  |  | q-value | 0.850 | 0.952 | 0.743 | 0.334 | 0.369 |  | 0.259 | 0.997 | 0.424 | 0.076 | 1.000 |  | 0.849 | 0.830 | 0.778 | 0.516 | 0.287 |
|  | |  |  |  |  |  |  |  |  |  |  |  |  |  |  |  |  |  |  |  |
| **multicellular organismal process** | |  |  |  |  |  |  |  |  |  |  |  |  |  |  |  |  |  |  |  |
|  | Regulation Of Tumor Necrosis Factor Superfamily Cytokine Production | 83 | p-value | 0.163 | 0.874 | 0.189 | 0.044** | 0.65 |  | 0.027** | 0.914 | 0.046** | 0.014** | 0.707 |  | 0.14 | 0.869 | 0.215 | 0.054* | 0.512 |
|  |  |  | q-value | 0.850 | 0.952 | 0.743 | 0.337 | 0.402 |  | 0.264 | 0.997 | 0.424 | 0.085 | 1.000 |  | 0.849 | 0.831 | 0.778 | 0.516 | 0.300 |
|  | Positive Regulation Of Type I Interferon Production | 64 | p-value | 0.87 | 0.166 | 0.129 | 0.075* | 0.128 |  | 0.169 | 0.427 | 0.044** | 0.007*** | 0.993 |  | 0.88 | 0.265 | 0.166 | 0.126 | 0.083* |
|  |  |  | q-value | 0.850 | 0.952 | 0.743 | 0.342 | 0.352 |  | 0.314 | 0.997 | 0.424 | 0.085 | 1.000 |  | 0.849 | 0.830 | 0.778 | 0.516 | 0.269 |
|  | Regulation Of Tumor Necrosis Factor Biosynthetic Process | 15 | p-value | 0.371 | 0.23 | 0.036** | 0.003*** | 0.504 |  | 0.06* | 0.23 | 0.013** | 0*** | 0.361 |  | 0.427 | 0.233 | 0.06* | 0.015** | 0.445 |
|  |  |  | q-value | 0.850 | 0.952 | 0.743 | 0.316 | 0.386 |  | 0.279 | 0.997 | 0.424 | 0.000 | 1.000 |  | 0.849 | 0.830 | 0.778 | 0.516 | 0.293 |
|  | Fertilization | 73 | p-value | 0.175 | 0.98 | 0.203 | 0.065* | 0.197 |  | 0.007*** | 0.978 | 0.014** | 0.003*** | 0.632 |  | 0.185 | 0.989 | 0.24 | 0.076* | 0.211 |
|  |  |  | q-value | 0.850 | 0.957 | 0.743 | 0.341 | 0.355 |  | 0.234 | 0.997 | 0.424 | 0.076 | 1.000 |  | 0.849 | 0.841 | 0.778 | 0.516 | 0.273 |
|  | Negative Regulation Of Endothelial Cell Migration | 25 | p-value | 0.196 | 0.83 | 0.299 | 0.071* | 0.131 |  | 0.011** | 0.86 | 0.019** | 0.005*** | 0.749 |  | 0.186 | 0.853 | 0.308 | 0.096* | 0.153 |
|  |  |  | q-value | 0.850 | 0.952 | 0.743 | 0.342 | 0.354 |  | 0.234 | 0.997 | 0.424 | 0.076 | 1.000 |  | 0.849 | 0.831 | 0.778 | 0.516 | 0.269 |
|  | |  |  |  |  |  |  |  |  |  |  |  |  |  |  |  |  |  |  |  |
| **multicellular organismal process: system process** | |  |  |  |  |  |  |  |  |  |  |  |  |  |  |  |  |  |  |  |
|  | Sensory Perception Of Mechanical Stimulus | 75 | p-value | 0.079* | 0.513 | 0.193 | 0.107 | 0.211 |  | 0.004*** | 0.329 | 0.015** | 0.019** | 0.215 |  | 0.086* | 0.488 | 0.204 | 0.11 | 0.256 |
|  |  |  | q-value | 0.850 | 0.952 | 0.743 | 0.353 | 0.355 |  | 0.223 | 0.997 | 0.424 | 0.085 | 1.000 |  | 0.849 | 0.830 | 0.778 | 0.516 | 0.279 |
|  | Positive Regulation Of Smooth Muscle Contraction | 18 | p-value | 0.042** | 0.566 | 0.138 | 0.071* | 0.76 |  | 0.004*** | 0.286 | 0.009*** | 0.037** | 0.06* |  | 0.054* | 0.471 | 0.135 | 0.059* | 0.698 |
|  |  |  | q-value | 0.850 | 0.952 | 0.743 | 0.342 | 0.419 |  | 0.223 | 0.997 | 0.424 | 0.087 | 1.000 |  | 0.849 | 0.830 | 0.778 | 0.516 | 0.323 |
|  | Regulation Of Vascular Permeability | 19 | p-value | 0.638 | 0.102 | 0.046** | 0.012** | 0.503 |  | 0.32 | 0.14 | 0.038** | 0.018** | 0.594 |  | 0.668 | 0.127 | 0.057* | 0.028** | 0.422 |
|  |  |  | q-value | 0.850 | 0.952 | 0.743 | 0.359 | 0.369 |  | 0.364 | 0.997 | 0.432 | 0.093 | 1.000 |  | 0.849 | 0.830 | 0.778 | 0.517 | 0.280 |
|  | |  |  |  |  |  |  |  |  |  |  |  |  |  |  |  |  |  |  |  |
| **multi-organism process** | |  |  |  |  |  |  |  |  |  |  |  |  |  |  |  |  |  |  |  |
|  | Response To Protozoan | 17 | p-value | 0.051* | 0.606 | 0.144 | 0.144 | 0.291 |  | 0.004*** | 0.514 | 0.017** | 0.036** | 0.102 |  | 0.07* | 0.578 | 0.188 | 0.188 | 0.24 |
|  |  |  | q-value | 0.850 | 0.952 | 0.743 | 0.356 | 0.367 |  | 0.223 | 0.997 | 0.424 | 0.087 | 1.000 |  | 0.849 | 0.830 | 0.778 | 0.516 | 0.279 |
|  | Negative Regulation Of Multi Organism Process | 107 | p-value | 0.005*** | 0.482 | 0.035** | 0.027** | 0.681 |  | 0.001*** | 0.369 | 0.002*** | 0.004*** | 0.08* |  | 0.014** | 0.468 | 0.032** | 0.032** | 0.63 |
|  |  |  | q-value | 0.850 | 0.952 | 0.743 | 0.337 | 0.405 |  | 0.144 | 0.997 | 0.424 | 0.076 | 1.000 |  | 0.849 | 0.830 | 0.778 | 0.516 | 0.314 |
|  | Response To Fungus | 31 | p-value | 0.726 | 0.255 | 0.093* | 0.048** | 0.154 |  | 0.112 | 0.52 | 0.029** | 0.005*** | 0.984 |  | 0.713 | 0.304 | 0.138 | 0.061* | 0.119 |
|  |  |  | q-value | 0.850 | 0.952 | 0.743 | 0.337 | 0.355 |  | 0.299 | 0.997 | 0.424 | 0.076 | 1.000 |  | 0.849 | 0.830 | 0.778 | 0.516 | 0.269 |
|  | |  |  |  |  |  |  |  |  |  |  |  |  |  |  |  |  |  |  |  |
| **others** | |  |  |  |  |  |  |  |  |  |  |  |  |  |  |  |  |  |  |  |
|  | Positive Regulation Of Cell Matrix Adhesion | 24 | p-value | 0.126 | 0.918 | 0.205 | 0.061* | 0.242 |  | 0.023** | 0.913 | 0.041** | 0.011** | 0.711 |  | 0.162 | 0.904 | 0.214 | 0.089* | 0.233 |
|  |  |  | q-value | 0.850 | 0.952 | 0.743 | 0.337 | 0.358 |  | 0.264 | 0.997 | 0.424 | 0.085 | 1.000 |  | 0.849 | 0.832 | 0.778 | 0.516 | 0.277 |
|  | Regulation Of Cation Transmembrane Transport | 118 | p-value | 0.371 | 0.503 | 0.099* | 0.017** | 0.107 |  | 0.027** | 0.798 | 0.014** | 0.001*** | 0.998 |  | 0.374 | 0.569 | 0.117 | 0.037** | 0.106 |
|  |  |  | q-value | 0.856 | 0.952 | 0.754 | 0.443 | 0.351 |  | 0.356 | 0.997 | 0.432 | 0.093 | 1.000 |  | 0.858 | 0.830 | 0.790 | 0.572 | 0.269 |

*Significance level of 0.1

**Significant level of 0.05

***Significance level of 0.001

† The multiple analysis of systolic and diastolic blood pressure measurements

‡The pulse pressure: difference between systolic and diastolic blood pressure values
