# Supplementary material for: Longitudinal linear combination test for gene set analysis
Source: BMC Bioinformatics. 2019 Dec 10;20:650. doi: 10.1186/s12859-019-3221-7 (PMC6902471; doi:10.1186/s12859-019-3221-7)
Supplement: Supplementary file 4 — Additional file 4. Results of LLCT analysis examining the differential time-course expressions of different gene sets in association with the position of the wound (skin or tongue). [file 12859_2019_3221_MOESM4_ESM.docx]

Additional File 4. Results of LLCT analysis examining the differential time-course expressions of different gene sets in association with the position of the wound (skin or tongue)

|  |  |  | Unwounded (time 0) | | Hemostasis (0-12 hours) | | Inflammation (12-72 hours) | | Proliferation (3-7 days) | | 0-7 days | |
| --- | --- | --- | --- | --- | --- | --- | --- | --- | --- | --- | --- | --- |
| KEGG pathway entry | KEGG Pathway Name (Mus musculus (mouse)) | Geneset size | p-value | q-value | p-value | q-value | p-value | q-value | p-value | q-value | p-value | q-value |
| mmu00010 | Glycolysis / Gluconeogenesis | 834 | 0.000 | 0.000 | 0.012 | 0.047 | 0.063 | 0.107 | 0.000 | 0.000 | 0.000 | 0.000 |
| mmu00020 | Citrate cycle (TCA cycle) | 468 | 0.000 | 0.000 | 0.579 | 0.732 | 0.000 | 0.000 | 0.963 | 0.981 | 0.004 | 0.006 |
| mmu00030 | Pentose phosphate pathway | 348 | 0.900 | 0.909 | 0.902 | 0.958 | 0.850 | 0.889 | 0.000 | 0.000 | 0.000 | 0.000 |
| mmu00040 | Pentose and glucuronate interconversions | 276 | 0.131 | 0.172 | 0.083 | 0.204 | 0.318 | 0.411 | 0.031 | 0.052 | 0.007 | 0.010 |
| mmu00051 | Fructose and mannose metabolism | 426 | 0.000 | 0.000 | 0.001 | 0.006 | 0.310 | 0.405 | 0.000 | 0.000 | 0.000 | 0.000 |
| mmu00052 | Galactose metabolism | 342 | 0.003 | 0.006 | 0.034 | 0.101 | 0.956 | 0.961 | 0.004 | 0.008 | 0.009 | 0.012 |
| mmu00053 | Ascorbate and aldarate metabolism | 174 | 0.527 | 0.590 | 0.294 | 0.469 | 0.952 | 0.961 | 0.008 | 0.015 | 0.025 | 0.032 |
| mmu00061 | Fatty acid biosynthesis | 222 | 0.455 | 0.524 | 0.940 | 0.965 | 0.689 | 0.758 | 0.174 | 0.222 | 0.367 | 0.386 |
| mmu00062 | Fatty acid elongation | 390 | 0.130 | 0.171 | 0.913 | 0.958 | 0.707 | 0.773 | 0.001 | 0.002 | 0.000 | 0.000 |
| mmu00071 | Fatty acid degradation | 498 | 0.007 | 0.012 | 0.002 | 0.011 | 0.375 | 0.464 | 0.357 | 0.423 | 0.003 | 0.004 |
| mmu00072 | Synthesis and degradation of ketone bodies | 120 | 0.602 | 0.662 | 0.033 | 0.100 | 0.954 | 0.961 | 0.423 | 0.492 | 0.017 | 0.022 |
| mmu00100 | Steroid biosynthesis | 240 | 0.000 | 0.000 | 0.002 | 0.011 | 0.100 | 0.160 | 0.200 | 0.248 | 0.001 | 0.002 |
| mmu00120 | Primary bile acid biosynthesis | 156 | 0.822 | 0.851 | 0.840 | 0.934 | 0.577 | 0.672 | 0.586 | 0.643 | 0.000 | 0.000 |
| mmu00130 | Ubiquinone and other terpenoid-quinone biosynthesis | 90 | 0.000 | 0.000 | 0.381 | 0.550 | 0.011 | 0.025 | 0.355 | 0.423 | 0.609 | 0.621 |
| mmu00140 | Steroid hormone biosynthesis | 606 | 0.091 | 0.125 | 0.067 | 0.170 | 0.114 | 0.177 | 0.000 | 0.000 | 0.000 | 0.000 |
| mmu00190 | Oxidative phosphorylation | 1452 | 0.000 | 0.000 | 0.003 | 0.015 | 0.151 | 0.221 | 0.809 | 0.853 | 0.000 | 0.000 |
| mmu00220 | Arginine biosynthesis | 222 | 0.000 | 0.000 | 0.208 | 0.375 | 0.802 | 0.859 | 0.014 | 0.025 | 0.010 | 0.014 |
| mmu00230 | Purine metabolism | 1578 | 0.000 | 0.000 | 0.000 | 0.000 | 0.014 | 0.030 | 0.002 | 0.004 | 0.000 | 0.000 |
| mmu00232 | Caffeine metabolism | 36 | 0.796 | 0.835 | 0.457 | 0.634 | 0.318 | 0.411 | 0.000 | 0.000 | 0.001 | 0.002 |
| mmu00240 | Pyrimidine metabolism | 702 | 0.032 | 0.048 | 0.691 | 0.813 | 0.000 | 0.000 | 0.580 | 0.640 | 0.524 | 0.546 |
| mmu00250 | Alanine, aspartate and glutamate metabolism | 450 | 0.015 | 0.024 | 0.155 | 0.315 | 0.343 | 0.436 | 0.111 | 0.156 | 0.007 | 0.010 |
| mmu00260 | Glycine, serine and threonine metabolism | 396 | 0.313 | 0.378 | 0.699 | 0.819 | 0.384 | 0.469 | 0.034 | 0.056 | 0.168 | 0.187 |
| mmu00270 | Cysteine and methionine metabolism | 642 | 0.000 | 0.000 | 0.147 | 0.301 | 0.126 | 0.190 | 0.988 | 0.994 | 0.226 | 0.245 |
| mmu00280 | Valine, leucine and isoleucine degradation | 612 | 0.000 | 0.000 | 0.055 | 0.147 | 0.001 | 0.003 | 0.303 | 0.368 | 0.046 | 0.056 |
| mmu00290 | Valine, leucine and isoleucine biosynthesis | 36 | 0.785 | 0.826 | 0.084 | 0.205 | 0.007 | 0.016 | 0.124 | 0.168 | 0.131 | 0.149 |
| mmu00310 | Lysine degradation | 768 | 0.005 | 0.009 | 0.002 | 0.011 | 0.191 | 0.276 | 0.200 | 0.248 | 0.012 | 0.016 |
| mmu00330 | Arginine and proline metabolism | 468 | 0.280 | 0.344 | 0.260 | 0.434 | 0.000 | 0.000 | 0.126 | 0.170 | 0.038 | 0.048 |
| mmu00340 | Histidine metabolism | 240 | 0.132 | 0.173 | 0.666 | 0.796 | 0.233 | 0.327 | 0.220 | 0.271 | 0.558 | 0.576 |
| mmu00350 | Tyrosine metabolism | 366 | 0.477 | 0.545 | 0.896 | 0.956 | 0.673 | 0.745 | 0.081 | 0.118 | 0.155 | 0.174 |
| mmu00360 | Phenylalanine metabolism | 210 | 0.166 | 0.213 | 0.083 | 0.204 | 0.855 | 0.891 | 0.004 | 0.008 | 0.002 | 0.003 |
| mmu00380 | Tryptophan metabolism | 456 | 0.021 | 0.033 | 0.850 | 0.938 | 0.318 | 0.411 | 0.148 | 0.193 | 0.531 | 0.552 |
| mmu00400 | Phenylalanine, tyrosine and tryptophan biosynthesis | 78 | 0.002 | 0.004 | 0.205 | 0.372 | 0.113 | 0.176 | 0.082 | 0.119 | 0.087 | 0.102 |
| mmu00410 | beta-Alanine metabolism | 318 | 0.122 | 0.161 | 0.247 | 0.421 | 0.058 | 0.102 | 0.119 | 0.162 | 0.048 | 0.058 |
| mmu00430 | Taurine and hypotaurine metabolism | 120 | 0.000 | 0.000 | 0.121 | 0.263 | 0.848 | 0.889 | 0.558 | 0.618 | 0.350 | 0.370 |
| mmu00440 | Phosphonate and phosphinate metabolism | 96 | 0.007 | 0.012 | 0.672 | 0.800 | 0.017 | 0.034 | 0.439 | 0.507 | 0.801 | 0.804 |
| mmu00450 | Selenocompound metabolism | 240 | 0.478 | 0.545 | 0.950 | 0.965 | 0.535 | 0.632 | 0.002 | 0.004 | 0.003 | 0.004 |
| mmu00471 | D-Glutamine and D-glutamate metabolism | 48 | 0.427 | 0.498 | 0.234 | 0.410 | 0.268 | 0.364 | 0.784 | 0.842 | 0.503 | 0.528 |
| mmu00472 | D-Arginine and D-ornithine metabolism | 6 | 0.105 | 0.141 | 0.727 | 0.841 | 0.220 | 0.315 | 0.311 | 0.374 | 0.597 | 0.612 |
| mmu00480 | Glutathione metabolism | 582 | 0.141 | 0.184 | 0.000 | 0.000 | 0.581 | 0.672 | 0.141 | 0.187 | 0.001 | 0.002 |
| mmu00500 | Starch and sucrose metabolism | 336 | 0.000 | 0.000 | 0.291 | 0.469 | 0.873 | 0.899 | 0.190 | 0.239 | 0.002 | 0.003 |
| mmu00510 | N-Glycan biosynthesis | 552 | 0.000 | 0.000 | 0.033 | 0.100 | 0.039 | 0.072 | 0.135 | 0.181 | 0.074 | 0.087 |
| mmu00511 | Other glycan degradation | 174 | 0.000 | 0.000 | 0.003 | 0.015 | 0.014 | 0.030 | 0.093 | 0.134 | 0.003 | 0.004 |
| mmu00512 | Mucin type O-glycan biosynthesis | 312 | 0.008 | 0.014 | 0.007 | 0.029 | 0.001 | 0.003 | 0.006 | 0.012 | 0.003 | 0.004 |
| mmu00514 | Other types of O-glycan biosynthesis | 246 | 0.000 | 0.000 | 0.065 | 0.167 | 0.101 | 0.160 | 0.027 | 0.046 | 0.002 | 0.003 |
| mmu00515 | Mannose type O-glycan biosynthesis | 282 | 0.046 | 0.066 | 0.138 | 0.290 | 0.874 | 0.899 | 0.622 | 0.677 | 0.029 | 0.037 |
| mmu00520 | Amino sugar and nucleotide sugar metabolism | 612 | 0.952 | 0.958 | 0.036 | 0.104 | 0.141 | 0.208 | 0.040 | 0.064 | 0.053 | 0.064 |
| mmu00524 | Neomycin, kanamycin and gentamicin biosynthesis | 60 | 0.161 | 0.208 | 0.269 | 0.442 | 0.806 | 0.859 | 0.115 | 0.158 | 0.272 | 0.292 |
| mmu00531 | Glycosaminoglycan degradation | 216 | 0.585 | 0.648 | 0.030 | 0.093 | 0.010 | 0.023 | 0.000 | 0.000 | 0.000 | 0.000 |
| mmu00532 | Glycosaminoglycan biosynthesis - chondroitin sulfate / dermatan sulfate | 174 | 0.000 | 0.000 | 0.557 | 0.710 | 0.018 | 0.036 | 0.543 | 0.606 | 0.779 | 0.784 |
| mmu00533 | Glycosaminoglycan biosynthesis - keratan sulfate | 144 | 0.032 | 0.048 | 0.646 | 0.778 | 0.377 | 0.464 | 0.068 | 0.102 | 0.071 | 0.084 |
| mmu00534 | Glycosaminoglycan biosynthesis - heparan sulfate / heparin | 306 | 0.079 | 0.110 | 0.607 | 0.742 | 0.689 | 0.758 | 0.062 | 0.094 | 0.312 | 0.333 |
| mmu00561 | Glycerolipid metabolism | 738 | 0.014 | 0.023 | 0.028 | 0.088 | 0.292 | 0.393 | 0.000 | 0.000 | 0.001 | 0.002 |
| mmu00562 | Inositol phosphate metabolism | 1008 | 0.415 | 0.489 | 0.585 | 0.732 | 0.398 | 0.483 | 0.543 | 0.606 | 0.550 | 0.570 |
| mmu00563 | Glycosylphosphatidylinositol (GPI)-anchor biosynthesis | 336 | 0.031 | 0.047 | 0.930 | 0.960 | 0.246 | 0.340 | 0.002 | 0.004 | 0.004 | 0.006 |
| mmu00564 | Glycerophospholipid metabolism | 1242 | 0.000 | 0.000 | 0.005 | 0.023 | 0.090 | 0.146 | 0.000 | 0.000 | 0.000 | 0.000 |
| mmu00565 | Ether lipid metabolism | 558 | 0.004 | 0.007 | 0.244 | 0.418 | 0.831 | 0.878 | 0.006 | 0.012 | 0.040 | 0.050 |
| mmu00590 | Arachidonic acid metabolism | 654 | 0.051 | 0.073 | 0.283 | 0.461 | 0.228 | 0.322 | 0.000 | 0.000 | 0.002 | 0.003 |
| mmu00591 | Linoleic acid metabolism | 354 | 0.117 | 0.156 | 0.186 | 0.345 | 0.972 | 0.972 | 0.018 | 0.032 | 0.017 | 0.022 |
| mmu00592 | alpha-Linolenic acid metabolism | 270 | 0.596 | 0.658 | 0.924 | 0.960 | 0.788 | 0.846 | 0.006 | 0.012 | 0.046 | 0.056 |
| mmu00600 | Sphingolipid metabolism | 594 | 0.003 | 0.006 | 0.629 | 0.766 | 0.579 | 0.672 | 0.000 | 0.000 | 0.000 | 0.000 |
| mmu00601 | Glycosphingolipid biosynthesis - lacto and neolacto series | 360 | 0.049 | 0.070 | 0.603 | 0.742 | 0.005 | 0.012 | 0.002 | 0.004 | 0.000 | 0.000 |
| mmu00603 | Glycosphingolipid biosynthesis - globo and isoglobo series | 180 | 0.008 | 0.014 | 0.844 | 0.935 | 0.358 | 0.446 | 0.039 | 0.063 | 0.196 | 0.216 |
| mmu00604 | Glycosphingolipid biosynthesis - ganglio series | 168 | 0.000 | 0.000 | 0.451 | 0.631 | 0.138 | 0.205 | 0.033 | 0.055 | 0.022 | 0.028 |
| mmu00620 | Pyruvate metabolism | 534 | 0.000 | 0.000 | 0.487 | 0.658 | 0.000 | 0.000 | 0.055 | 0.086 | 0.079 | 0.093 |
| mmu00630 | Glyoxylate and dicarboxylate metabolism | 378 | 0.000 | 0.000 | 0.279 | 0.456 | 0.004 | 0.010 | 0.804 | 0.853 | 0.090 | 0.105 |
| mmu00640 | Propanoate metabolism | 300 | 0.000 | 0.000 | 0.910 | 0.958 | 0.000 | 0.000 | 0.875 | 0.900 | 0.512 | 0.536 |
| mmu00650 | Butanoate metabolism | 264 | 0.816 | 0.851 | 0.006 | 0.027 | 0.261 | 0.356 | 0.515 | 0.581 | 0.038 | 0.048 |
| mmu00670 | One carbon pool by folate | 264 | 0.000 | 0.000 | 0.000 | 0.000 | 0.004 | 0.010 | 0.983 | 0.994 | 0.000 | 0.000 |
| mmu00730 | Thiamine metabolism | 198 | 0.011 | 0.018 | 0.063 | 0.163 | 0.250 | 0.344 | 0.004 | 0.008 | 0.007 | 0.010 |
| mmu00740 | Riboflavin metabolism | 132 | 0.838 | 0.865 | 0.017 | 0.062 | 0.294 | 0.394 | 0.008 | 0.015 | 0.003 | 0.004 |
| mmu00750 | Vitamin B6 metabolism | 78 | 0.016 | 0.025 | 0.156 | 0.315 | 0.869 | 0.899 | 0.143 | 0.189 | 0.002 | 0.003 |
| mmu00760 | Nicotinate and nicotinamide metabolism | 378 | 0.000 | 0.000 | 0.701 | 0.819 | 0.024 | 0.046 | 0.696 | 0.750 | 0.658 | 0.666 |
| mmu00770 | Pantothenate and CoA biosynthesis | 270 | 0.001 | 0.002 | 0.960 | 0.972 | 0.003 | 0.008 | 0.192 | 0.240 | 0.561 | 0.577 |
| mmu00780 | Biotin metabolism | 42 | 0.682 | 0.733 | 0.495 | 0.663 | 0.748 | 0.809 | 0.007 | 0.014 | 0.012 | 0.016 |
| mmu00785 | Lipoic acid metabolism | 36 | 0.000 | 0.000 | 0.393 | 0.565 | 0.591 | 0.681 | 0.321 | 0.385 | 0.171 | 0.190 |
| mmu00790 | Folate biosynthesis | 234 | 0.000 | 0.000 | 0.043 | 0.119 | 0.000 | 0.000 | 0.474 | 0.542 | 0.161 | 0.180 |
| mmu00830 | Retinol metabolism | 510 | 0.856 | 0.881 | 0.989 | 0.992 | 0.910 | 0.927 | 0.008 | 0.015 | 0.047 | 0.057 |
| mmu00860 | Porphyrin and chlorophyll metabolism | 420 | 0.000 | 0.000 | 0.448 | 0.630 | 0.000 | 0.000 | 0.001 | 0.002 | 0.002 | 0.003 |
| mmu00900 | Terpenoid backbone biosynthesis | 294 | 0.000 | 0.000 | 0.107 | 0.239 | 0.302 | 0.400 | 0.234 | 0.287 | 0.318 | 0.338 |
| mmu00910 | Nitrogen metabolism | 192 | 0.553 | 0.615 | 0.343 | 0.518 | 0.507 | 0.607 | 0.003 | 0.007 | 0.024 | 0.031 |
| mmu00920 | Sulfur metabolism | 96 | 0.000 | 0.000 | 0.486 | 0.658 | 0.525 | 0.625 | 0.046 | 0.073 | 0.193 | 0.213 |
| mmu00970 | Aminoacyl-tRNA biosynthesis | 510 | 0.000 | 0.000 | 0.000 | 0.000 | 0.357 | 0.446 | 0.803 | 0.853 | 0.000 | 0.000 |
| mmu00980 | Metabolism of xenobiotics by cytochrome P450 | 456 | 0.028 | 0.043 | 0.176 | 0.332 | 0.754 | 0.813 | 0.022 | 0.038 | 0.029 | 0.037 |
| mmu00982 | Drug metabolism - cytochrome P450 | 468 | 0.000 | 0.000 | 0.108 | 0.239 | 0.072 | 0.120 | 0.080 | 0.117 | 0.036 | 0.045 |
| mmu00983 | Drug metabolism - other enzymes | 744 | 0.095 | 0.130 | 0.519 | 0.679 | 0.003 | 0.008 | 0.368 | 0.435 | 0.733 | 0.740 |
| mmu01040 | Biosynthesis of unsaturated fatty acids | 420 | 0.000 | 0.000 | 0.215 | 0.383 | 0.571 | 0.667 | 0.875 | 0.900 | 0.000 | 0.000 |
| mmu02010 | ABC transporters | 528 | 0.399 | 0.471 | 0.510 | 0.672 | 0.015 | 0.031 | 0.078 | 0.115 | 0.079 | 0.093 |
| mmu03008 | Ribosome biogenesis in eukaryotes | 972 | 0.000 | 0.000 | 0.000 | 0.000 | 0.224 | 0.318 | 0.001 | 0.002 | 0.000 | 0.000 |
| mmu03010 | Ribosome | 1932 | 0.000 | 0.000 | 0.537 | 0.694 | 0.001 | 0.003 | 0.000 | 0.000 | 0.000 | 0.000 |
| mmu03013 | RNA transport | 2244 | 0.000 | 0.000 | 0.000 | 0.000 | 0.723 | 0.786 | 0.001 | 0.002 | 0.000 | 0.000 |
| mmu03015 | mRNA surveillance pathway | 1362 | 0.000 | 0.000 | 0.002 | 0.011 | 0.337 | 0.432 | 0.000 | 0.000 | 0.000 | 0.000 |
| mmu03018 | RNA degradation | 1044 | 0.001 | 0.002 | 0.000 | 0.000 | 0.076 | 0.126 | 0.810 | 0.853 | 0.006 | 0.009 |
| mmu03020 | RNA polymerase | 372 | 0.984 | 0.984 | 0.000 | 0.000 | 0.646 | 0.726 | 0.166 | 0.213 | 0.001 | 0.002 |
| mmu03022 | Basal transcription factors | 468 | 0.016 | 0.025 | 0.168 | 0.325 | 0.340 | 0.434 | 0.103 | 0.145 | 0.004 | 0.006 |
| mmu03030 | DNA replication | 474 | 0.000 | 0.000 | 0.000 | 0.000 | 0.000 | 0.000 | 0.000 | 0.000 | 0.000 | 0.000 |
| mmu03040 | Spliceosome | 1968 | 0.000 | 0.000 | 0.000 | 0.000 | 0.000 | 0.000 | 0.000 | 0.000 | 0.000 | 0.000 |
| mmu03050 | Proteasome | 576 | 0.044 | 0.064 | 0.000 | 0.000 | 0.835 | 0.879 | 0.526 | 0.591 | 0.000 | 0.000 |
| mmu03060 | Protein export | 372 | 0.064 | 0.090 | 0.005 | 0.023 | 0.381 | 0.467 | 0.076 | 0.112 | 0.003 | 0.004 |
| mmu03320 | PPAR signaling pathway | 930 | 0.486 | 0.550 | 0.095 | 0.220 | 0.336 | 0.432 | 0.000 | 0.000 | 0.000 | 0.000 |
| mmu03410 | Base excision repair | 420 | 0.000 | 0.000 | 0.022 | 0.075 | 0.122 | 0.186 | 0.000 | 0.000 | 0.000 | 0.000 |
| mmu03420 | Nucleotide excision repair | 582 | 0.006 | 0.011 | 0.499 | 0.666 | 0.346 | 0.438 | 0.000 | 0.000 | 0.000 | 0.000 |
| mmu03430 | Mismatch repair | 270 | 0.000 | 0.000 | 0.003 | 0.015 | 0.001 | 0.003 | 0.000 | 0.000 | 0.000 | 0.000 |
| mmu03440 | Homologous recombination | 480 | 0.000 | 0.000 | 0.001 | 0.006 | 0.129 | 0.193 | 0.001 | 0.002 | 0.000 | 0.000 |
| mmu03450 | Non-homologous end-joining | 132 | 0.104 | 0.141 | 0.512 | 0.672 | 0.067 | 0.112 | 0.934 | 0.958 | 0.876 | 0.876 |
| mmu03460 | Fanconi anemia pathway | 636 | 0.000 | 0.000 | 0.044 | 0.121 | 0.177 | 0.257 | 0.103 | 0.145 | 0.018 | 0.023 |
| mmu04010 | MAPK signaling pathway | 4254 | 0.089 | 0.123 | 0.024 | 0.080 | 0.022 | 0.043 | 0.000 | 0.000 | 0.000 | 0.000 |
| mmu04012 | ErbB signaling pathway | 1440 | 0.002 | 0.004 | 0.340 | 0.516 | 0.101 | 0.160 | 0.000 | 0.000 | 0.000 | 0.000 |
| mmu04014 | Ras signaling pathway | 3132 | 0.230 | 0.285 | 0.020 | 0.070 | 0.008 | 0.018 | 0.000 | 0.000 | 0.000 | 0.000 |
| mmu04015 | Rap1 signaling pathway | 3048 | 0.290 | 0.353 | 0.373 | 0.541 | 0.021 | 0.042 | 0.000 | 0.000 | 0.000 | 0.000 |
| mmu04020 | Calcium signaling pathway | 2412 | 0.000 | 0.000 | 0.007 | 0.029 | 0.000 | 0.000 | 0.000 | 0.000 | 0.000 | 0.000 |
| mmu04022 | cGMP-PKG signaling pathway | 2448 | 0.118 | 0.157 | 0.233 | 0.410 | 0.000 | 0.000 | 0.000 | 0.000 | 0.000 | 0.000 |
| mmu04024 | cAMP signaling pathway | 2766 | 0.115 | 0.154 | 0.129 | 0.278 | 0.000 | 0.000 | 0.000 | 0.000 | 0.000 | 0.000 |
| mmu04060 | Cytokine-cytokine receptor interaction | 2616 | 0.000 | 0.000 | 0.000 | 0.000 | 0.000 | 0.000 | 0.000 | 0.000 | 0.000 | 0.000 |
| mmu04061 | Viral protein interaction with cytokine and cytokine receptor | 834 | 0.000 | 0.000 | 0.165 | 0.325 | 0.000 | 0.000 | 0.000 | 0.000 | 0.000 | 0.000 |
| mmu04062 | Chemokine signaling pathway | 2484 | 0.000 | 0.000 | 0.189 | 0.349 | 0.000 | 0.000 | 0.000 | 0.000 | 0.000 | 0.000 |
| mmu04064 | NF-kappa B signaling pathway | 1404 | 0.000 | 0.000 | 0.000 | 0.000 | 0.000 | 0.000 | 0.000 | 0.000 | 0.000 | 0.000 |
| mmu04066 | HIF-1 signaling pathway | 1608 | 0.000 | 0.000 | 0.000 | 0.000 | 0.000 | 0.000 | 0.000 | 0.000 | 0.000 | 0.000 |
| mmu04068 | FoxO signaling pathway | 2016 | 0.002 | 0.004 | 0.001 | 0.006 | 0.115 | 0.177 | 0.868 | 0.900 | 0.000 | 0.000 |
| mmu04070 | Phosphatidylinositol signaling system | 1386 | 0.381 | 0.453 | 0.757 | 0.869 | 0.016 | 0.033 | 0.119 | 0.162 | 0.173 | 0.192 |
| mmu04071 | Sphingolipid signaling pathway | 1818 | 0.173 | 0.219 | 0.130 | 0.278 | 0.000 | 0.000 | 0.000 | 0.000 | 0.000 | 0.000 |
| mmu04072 | Phospholipase D signaling pathway | 2052 | 0.009 | 0.015 | 0.040 | 0.112 | 0.534 | 0.632 | 0.000 | 0.000 | 0.000 | 0.000 |
| mmu04080 | Neuroactive ligand-receptor interaction | 3114 | 0.698 | 0.747 | 0.002 | 0.011 | 0.000 | 0.000 | 0.000 | 0.000 | 0.000 | 0.000 |
| mmu04110 | Cell cycle | 1914 | 0.000 | 0.000 | 0.000 | 0.000 | 0.673 | 0.745 | 0.000 | 0.000 | 0.000 | 0.000 |
| mmu04114 | Oocyte meiosis | 1770 | 0.009 | 0.015 | 0.352 | 0.522 | 0.622 | 0.713 | 0.139 | 0.186 | 0.103 | 0.119 |
| mmu04115 | p53 signaling pathway | 1020 | 0.000 | 0.000 | 0.001 | 0.006 | 0.045 | 0.083 | 0.066 | 0.100 | 0.007 | 0.010 |
| mmu04120 | Ubiquitin mediated proteolysis | 2340 | 0.671 | 0.728 | 0.989 | 0.992 | 0.063 | 0.107 | 0.164 | 0.211 | 0.609 | 0.621 |
| mmu04122 | Sulfur relay system | 102 | 0.007 | 0.012 | 0.040 | 0.112 | 0.869 | 0.899 | 0.276 | 0.336 | 0.104 | 0.119 |
| mmu04130 | SNARE interactions in vesicular transport | 528 | 0.036 | 0.053 | 0.095 | 0.220 | 0.120 | 0.184 | 0.062 | 0.094 | 0.094 | 0.109 |
| mmu04136 | Autophagy - other | 462 | 0.428 | 0.498 | 0.250 | 0.424 | 0.000 | 0.000 | 0.011 | 0.020 | 0.005 | 0.007 |
| mmu04137 | Mitophagy - animal | 978 | 0.054 | 0.077 | 0.235 | 0.410 | 0.286 | 0.387 | 0.014 | 0.025 | 0.009 | 0.012 |
| mmu04140 | Autophagy - animal | 1992 | 0.101 | 0.137 | 0.002 | 0.011 | 0.022 | 0.043 | 0.001 | 0.002 | 0.000 | 0.000 |
| mmu04141 | Protein processing in endoplasmic reticulum | 2328 | 0.000 | 0.000 | 0.000 | 0.000 | 0.000 | 0.000 | 0.000 | 0.000 | 0.000 | 0.000 |
| mmu04142 | Lysosome | 1542 | 0.000 | 0.000 | 0.000 | 0.000 | 0.000 | 0.000 | 0.000 | 0.000 | 0.000 | 0.000 |
| mmu04144 | Endocytosis | 3696 | 0.000 | 0.000 | 0.162 | 0.323 | 0.222 | 0.316 | 0.000 | 0.000 | 0.000 | 0.000 |
| mmu04145 | Phagosome | 1908 | 0.000 | 0.000 | 0.399 | 0.571 | 0.000 | 0.000 | 0.000 | 0.000 | 0.000 | 0.000 |
| mmu04146 | Peroxisome | 1002 | 0.177 | 0.223 | 0.591 | 0.736 | 0.013 | 0.028 | 0.652 | 0.707 | 0.022 | 0.028 |
| mmu04150 | mTOR signaling pathway | 2322 | 0.044 | 0.064 | 0.000 | 0.000 | 0.000 | 0.000 | 0.112 | 0.157 | 0.000 | 0.000 |
| mmu04151 | PI3K-Akt signaling pathway | 4854 | 0.000 | 0.000 | 0.948 | 0.965 | 0.000 | 0.000 | 0.000 | 0.000 | 0.000 | 0.000 |
| mmu04152 | AMPK signaling pathway | 1902 | 0.084 | 0.117 | 0.000 | 0.000 | 0.299 | 0.399 | 0.000 | 0.000 | 0.000 | 0.000 |
| mmu04210 | Apoptosis | 1902 | 0.000 | 0.000 | 0.030 | 0.093 | 0.000 | 0.000 | 0.000 | 0.000 | 0.000 | 0.000 |
| mmu04211 | Longevity regulating pathway | 1416 | 0.327 | 0.392 | 0.076 | 0.189 | 0.142 | 0.209 | 0.099 | 0.142 | 0.008 | 0.011 |
| mmu04213 | Longevity regulating pathway - multiple species | 978 | 0.480 | 0.545 | 0.351 | 0.522 | 0.628 | 0.713 | 0.984 | 0.994 | 0.002 | 0.003 |
| mmu04215 | Apoptosis - multiple species | 504 | 0.001 | 0.002 | 0.559 | 0.710 | 0.000 | 0.000 | 0.185 | 0.233 | 0.054 | 0.065 |
| mmu04216 | Ferroptosis | 576 | 0.006 | 0.011 | 0.117 | 0.256 | 0.002 | 0.006 | 0.009 | 0.017 | 0.002 | 0.003 |
| mmu04217 | Necroptosis | 1698 | 0.893 | 0.904 | 0.008 | 0.033 | 0.000 | 0.000 | 0.000 | 0.000 | 0.000 | 0.000 |
| mmu04218 | Cellular senescence | 2430 | 0.000 | 0.000 | 0.210 | 0.376 | 0.217 | 0.312 | 0.471 | 0.540 | 0.211 | 0.230 |
| mmu04260 | Cardiac muscle contraction | 1104 | 0.000 | 0.000 | 0.001 | 0.006 | 0.096 | 0.154 | 0.039 | 0.063 | 0.000 | 0.000 |
| mmu04261 | Adrenergic signaling in cardiomyocytes | 2184 | 0.000 | 0.000 | 0.003 | 0.015 | 0.006 | 0.014 | 0.002 | 0.004 | 0.000 | 0.000 |
| mmu04270 | Vascular smooth muscle contraction | 1752 | 0.045 | 0.065 | 0.877 | 0.952 | 0.008 | 0.018 | 0.004 | 0.008 | 0.000 | 0.000 |
| mmu04310 | Wnt signaling pathway | 2358 | 0.000 | 0.000 | 0.000 | 0.000 | 0.000 | 0.000 | 0.073 | 0.109 | 0.000 | 0.000 |
| mmu04330 | Notch signaling pathway | 558 | 0.000 | 0.000 | 0.025 | 0.081 | 0.559 | 0.656 | 0.144 | 0.189 | 0.042 | 0.052 |
| mmu04340 | Hedgehog signaling pathway | 756 | 0.000 | 0.000 | 0.000 | 0.000 | 0.007 | 0.016 | 0.005 | 0.010 | 0.000 | 0.000 |
| mmu04350 | TGF-beta signaling pathway | 1272 | 0.000 | 0.000 | 0.003 | 0.015 | 0.708 | 0.773 | 0.005 | 0.010 | 0.000 | 0.000 |
| mmu04360 | Axon guidance | 2778 | 0.000 | 0.000 | 0.000 | 0.000 | 0.000 | 0.000 | 0.018 | 0.032 | 0.000 | 0.000 |
| mmu04370 | VEGF signaling pathway | 864 | 0.300 | 0.364 | 0.462 | 0.637 | 0.894 | 0.917 | 0.000 | 0.000 | 0.001 | 0.002 |
| mmu04371 | Apelin signaling pathway | 1920 | 0.808 | 0.845 | 0.941 | 0.965 | 0.002 | 0.006 | 0.000 | 0.000 | 0.000 | 0.000 |
| mmu04380 | Osteoclast differentiation | 1662 | 0.000 | 0.000 | 0.886 | 0.952 | 0.000 | 0.000 | 0.000 | 0.000 | 0.000 | 0.000 |
| mmu04390 | Hippo signaling pathway | 2220 | 0.000 | 0.000 | 0.000 | 0.000 | 0.000 | 0.000 | 0.997 | 1.000 | 0.000 | 0.000 |
| mmu04392 | Hippo signaling pathway - multiple species | 378 | 0.000 | 0.000 | 0.837 | 0.934 | 0.000 | 0.000 | 0.378 | 0.445 | 0.140 | 0.158 |
| mmu04510 | Focal adhesion | 2826 | 0.000 | 0.000 | 0.917 | 0.959 | 0.000 | 0.000 | 0.000 | 0.000 | 0.000 | 0.000 |
| mmu04512 | ECM-receptor interaction | 1026 | 0.000 | 0.000 | 0.512 | 0.672 | 0.055 | 0.098 | 0.000 | 0.000 | 0.000 | 0.000 |
| mmu04514 | Cell adhesion molecules (CAMs) | 2016 | 0.000 | 0.000 | 0.546 | 0.699 | 0.000 | 0.000 | 0.000 | 0.000 | 0.001 | 0.002 |
| mmu04520 | Adherens junction | 1218 | 0.000 | 0.000 | 0.017 | 0.062 | 0.006 | 0.014 | 0.006 | 0.012 | 0.002 | 0.003 |
| mmu04530 | Tight junction | 2256 | 0.007 | 0.012 | 0.584 | 0.732 | 0.047 | 0.086 | 0.000 | 0.000 | 0.000 | 0.000 |
| mmu04540 | Gap junction | 1188 | 0.165 | 0.212 | 0.017 | 0.062 | 0.659 | 0.738 | 0.001 | 0.002 | 0.000 | 0.000 |
| mmu04550 | Signaling pathways regulating pluripotency of stem cells | 2016 | 0.000 | 0.000 | 0.000 | 0.000 | 0.000 | 0.000 | 0.308 | 0.372 | 0.000 | 0.000 |
| mmu04610 | Complement and coagulation cascades | 732 | 0.048 | 0.069 | 0.912 | 0.958 | 0.026 | 0.050 | 0.616 | 0.673 | 0.636 | 0.646 |
| mmu04611 | Platelet activation | 1680 | 0.887 | 0.904 | 0.216 | 0.383 | 0.000 | 0.000 | 0.000 | 0.000 | 0.000 | 0.000 |
| mmu04612 | Antigen processing and presentation | 870 | 0.000 | 0.000 | 0.307 | 0.482 | 0.000 | 0.000 | 0.004 | 0.008 | 0.011 | 0.015 |
| mmu04614 | Renin-angiotensin system | 312 | 0.441 | 0.512 | 0.251 | 0.424 | 0.038 | 0.070 | 0.209 | 0.258 | 0.290 | 0.310 |
| mmu04620 | Toll-like receptor signaling pathway | 1230 | 0.001 | 0.002 | 0.005 | 0.023 | 0.000 | 0.000 | 0.000 | 0.000 | 0.000 | 0.000 |
| mmu04621 | NOD-like receptor signaling pathway | 2040 | 0.000 | 0.000 | 0.092 | 0.219 | 0.000 | 0.000 | 0.000 | 0.000 | 0.000 | 0.000 |
| mmu04622 | RIG-I-like receptor signaling pathway | 852 | 0.017 | 0.027 | 0.015 | 0.056 | 0.000 | 0.000 | 0.000 | 0.000 | 0.000 | 0.000 |
| mmu04623 | Cytosolic DNA-sensing pathway | 690 | 0.002 | 0.004 | 0.000 | 0.000 | 0.000 | 0.000 | 0.002 | 0.004 | 0.001 | 0.002 |
| mmu04625 | C-type lectin receptor signaling pathway | 1530 | 0.000 | 0.000 | 0.099 | 0.226 | 0.000 | 0.000 | 0.000 | 0.000 | 0.000 | 0.000 |
| mmu04630 | JAK-STAT signaling pathway | 1944 | 0.000 | 0.000 | 0.000 | 0.000 | 0.000 | 0.000 | 0.000 | 0.000 | 0.000 | 0.000 |
| mmu04640 | Hematopoietic cell lineage | 984 | 0.000 | 0.000 | 0.011 | 0.044 | 0.000 | 0.000 | 0.000 | 0.000 | 0.000 | 0.000 |
| mmu04650 | Natural killer cell mediated cytotoxicity | 1440 | 0.000 | 0.000 | 0.015 | 0.056 | 0.001 | 0.003 | 0.000 | 0.000 | 0.000 | 0.000 |
| mmu04657 | IL-17 signaling pathway | 1104 | 0.000 | 0.000 | 0.002 | 0.011 | 0.000 | 0.000 | 0.000 | 0.000 | 0.000 | 0.000 |
| mmu04658 | Th1 and Th2 cell differentiation | 1188 | 0.000 | 0.000 | 0.837 | 0.934 | 0.000 | 0.000 | 0.000 | 0.000 | 0.000 | 0.000 |
| mmu04659 | Th17 cell differentiation | 1458 | 0.000 | 0.000 | 0.865 | 0.948 | 0.000 | 0.000 | 0.000 | 0.000 | 0.000 | 0.000 |
| mmu04660 | T cell receptor signaling pathway | 1596 | 0.000 | 0.000 | 0.312 | 0.487 | 0.095 | 0.153 | 0.000 | 0.000 | 0.000 | 0.000 |
| mmu04662 | B cell receptor signaling pathway | 1080 | 0.000 | 0.000 | 0.027 | 0.086 | 0.000 | 0.000 | 0.000 | 0.000 | 0.001 | 0.002 |
| mmu04664 | Fc epsilon RI signaling pathway | 888 | 0.246 | 0.304 | 0.021 | 0.072 | 0.013 | 0.028 | 0.000 | 0.000 | 0.000 | 0.000 |
| mmu04666 | Fc gamma R-mediated phagocytosis | 1392 | 0.006 | 0.011 | 0.037 | 0.105 | 0.000 | 0.000 | 0.000 | 0.000 | 0.000 | 0.000 |
| mmu04668 | TNF signaling pathway | 1470 | 0.000 | 0.000 | 0.293 | 0.469 | 0.000 | 0.000 | 0.000 | 0.000 | 0.000 | 0.000 |
| mmu04670 | Leukocyte transendothelial migration | 1506 | 0.005 | 0.009 | 0.025 | 0.081 | 0.002 | 0.006 | 0.000 | 0.000 | 0.000 | 0.000 |
| mmu04672 | Intestinal immune network for IgA production | 498 | 0.000 | 0.000 | 0.490 | 0.660 | 0.000 | 0.000 | 0.000 | 0.000 | 0.001 | 0.002 |
| mmu04710 | Circadian rhythm | 534 | 0.028 | 0.043 | 0.096 | 0.220 | 0.940 | 0.955 | 0.184 | 0.233 | 0.048 | 0.058 |
| mmu04713 | Circadian entrainment | 1398 | 0.000 | 0.000 | 0.146 | 0.300 | 0.003 | 0.008 | 0.003 | 0.007 | 0.000 | 0.000 |
| mmu04714 | Thermogenesis | 2856 | 0.000 | 0.000 | 0.058 | 0.154 | 0.004 | 0.010 | 0.959 | 0.981 | 0.000 | 0.000 |
| mmu04720 | Long-term potentiation | 1086 | 0.727 | 0.773 | 0.049 | 0.132 | 0.037 | 0.069 | 0.423 | 0.492 | 0.000 | 0.000 |
| mmu04721 | Synaptic vesicle cycle | 1074 | 0.891 | 0.904 | 0.320 | 0.493 | 0.000 | 0.000 | 0.000 | 0.000 | 0.000 | 0.000 |
| mmu04722 | Neurotrophin signaling pathway | 1998 | 0.000 | 0.000 | 0.930 | 0.960 | 0.241 | 0.336 | 0.000 | 0.000 | 0.000 | 0.000 |
| mmu04723 | Retrograde endocannabinoid signaling | 1956 | 0.000 | 0.000 | 0.002 | 0.011 | 0.000 | 0.000 | 0.549 | 0.610 | 0.000 | 0.000 |
| mmu04724 | Glutamatergic synapse | 1554 | 0.002 | 0.004 | 0.021 | 0.072 | 0.000 | 0.000 | 0.005 | 0.010 | 0.000 | 0.000 |
| mmu04725 | Cholinergic synapse | 1662 | 0.263 | 0.324 | 0.026 | 0.084 | 0.001 | 0.003 | 0.000 | 0.000 | 0.000 | 0.000 |
| mmu04726 | Serotonergic synapse | 1584 | 0.288 | 0.352 | 0.265 | 0.438 | 0.015 | 0.031 | 0.000 | 0.000 | 0.000 | 0.000 |
| mmu04727 | GABAergic synapse | 1158 | 0.196 | 0.246 | 0.110 | 0.242 | 0.080 | 0.132 | 0.014 | 0.025 | 0.000 | 0.000 |
| mmu04728 | Dopaminergic synapse | 2082 | 0.000 | 0.000 | 0.000 | 0.000 | 0.002 | 0.006 | 0.010 | 0.019 | 0.000 | 0.000 |
| mmu04730 | Long-term depression | 1014 | 0.149 | 0.193 | 0.686 | 0.812 | 0.508 | 0.607 | 0.000 | 0.000 | 0.000 | 0.000 |
| mmu04740 | Olfactory transduction | 810 | 0.000 | 0.000 | 0.000 | 0.000 | 0.059 | 0.103 | 0.023 | 0.039 | 0.000 | 0.000 |
| mmu04742 | Taste transduction | 570 | 0.037 | 0.055 | 0.357 | 0.525 | 0.351 | 0.443 | 0.102 | 0.145 | 0.000 | 0.000 |
| mmu04744 | Phototransduction | 264 | 0.000 | 0.000 | 0.011 | 0.044 | 0.172 | 0.251 | 0.498 | 0.563 | 0.057 | 0.068 |
| mmu04750 | Inflammatory mediator regulation of TRP channels | 1434 | 0.417 | 0.489 | 0.244 | 0.418 | 0.013 | 0.028 | 0.049 | 0.077 | 0.000 | 0.000 |
| mmu04810 | Regulation of actin cytoskeleton | 3030 | 0.000 | 0.000 | 0.034 | 0.101 | 0.104 | 0.163 | 0.000 | 0.000 | 0.000 | 0.000 |
| mmu04910 | Insulin signaling pathway | 2070 | 0.515 | 0.578 | 0.877 | 0.952 | 0.301 | 0.400 | 0.000 | 0.000 | 0.000 | 0.000 |
| mmu04911 | Insulin secretion | 1200 | 0.006 | 0.011 | 0.035 | 0.103 | 0.087 | 0.142 | 0.000 | 0.000 | 0.000 | 0.000 |
| mmu04912 | GnRH signaling pathway | 1350 | 0.345 | 0.412 | 0.070 | 0.176 | 0.243 | 0.337 | 0.000 | 0.000 | 0.000 | 0.000 |
| mmu04913 | Ovarian steroidogenesis | 624 | 0.068 | 0.095 | 0.868 | 0.948 | 0.668 | 0.745 | 0.270 | 0.330 | 0.125 | 0.142 |
| mmu04914 | Progesterone-mediated oocyte maturation | 1344 | 0.029 | 0.044 | 0.001 | 0.006 | 0.478 | 0.578 | 0.875 | 0.900 | 0.044 | 0.054 |
| mmu04915 | Estrogen signaling pathway | 1860 | 0.000 | 0.000 | 0.751 | 0.865 | 0.126 | 0.190 | 0.000 | 0.000 | 0.000 | 0.000 |
| mmu04916 | Melanogenesis | 1452 | 0.000 | 0.000 | 0.000 | 0.000 | 0.000 | 0.000 | 0.480 | 0.547 | 0.000 | 0.000 |
| mmu04917 | Prolactin signaling pathway | 1098 | 0.025 | 0.039 | 0.487 | 0.658 | 0.013 | 0.028 | 0.114 | 0.157 | 0.001 | 0.002 |
| mmu04918 | Thyroid hormone synthesis | 1008 | 0.001 | 0.002 | 0.253 | 0.425 | 0.063 | 0.107 | 0.004 | 0.008 | 0.000 | 0.000 |
| mmu04919 | Thyroid hormone signaling pathway | 1812 | 0.022 | 0.034 | 0.107 | 0.239 | 0.555 | 0.653 | 0.009 | 0.017 | 0.001 | 0.002 |
| mmu04920 | Adipocytokine signaling pathway | 1008 | 0.761 | 0.804 | 0.728 | 0.841 | 0.005 | 0.012 | 0.000 | 0.000 | 0.000 | 0.000 |
| mmu04921 | Oxytocin signaling pathway | 2250 | 0.000 | 0.000 | 0.002 | 0.011 | 0.001 | 0.003 | 0.000 | 0.000 | 0.000 | 0.000 |
| mmu04922 | Glucagon signaling pathway | 1392 | 0.056 | 0.079 | 0.290 | 0.469 | 0.016 | 0.033 | 0.184 | 0.233 | 0.012 | 0.016 |
| mmu04923 | Regulation of lipolysis in adipocytes | 744 | 0.038 | 0.056 | 0.146 | 0.300 | 0.027 | 0.052 | 0.057 | 0.088 | 0.000 | 0.000 |
| mmu04924 | Renin secretion | 1026 | 0.606 | 0.664 | 0.351 | 0.522 | 0.000 | 0.000 | 0.039 | 0.063 | 0.028 | 0.036 |
| mmu04925 | Aldosterone synthesis and secretion | 1458 | 0.871 | 0.893 | 0.159 | 0.319 | 0.001 | 0.003 | 0.060 | 0.092 | 0.000 | 0.000 |
| mmu04926 | Relaxin signaling pathway | 1818 | 0.007 | 0.012 | 0.882 | 0.952 | 0.009 | 0.021 | 0.000 | 0.000 | 0.000 | 0.000 |
| mmu04927 | Cortisol synthesis and secretion | 930 | 0.000 | 0.000 | 0.328 | 0.503 | 0.006 | 0.014 | 0.039 | 0.063 | 0.000 | 0.000 |
| mmu04928 | Parathyroid hormone synthesis, secretion and action | 1626 | 0.223 | 0.279 | 0.970 | 0.979 | 0.014 | 0.030 | 0.002 | 0.004 | 0.002 | 0.003 |
| mmu04930 | Type II diabetes mellitus | 690 | 0.496 | 0.559 | 0.144 | 0.300 | 0.000 | 0.000 | 0.052 | 0.082 | 0.000 | 0.000 |
| mmu04931 | Insulin resistance | 1542 | 0.001 | 0.002 | 0.166 | 0.325 | 0.000 | 0.000 | 0.006 | 0.012 | 0.000 | 0.000 |
| mmu04932 | Non-alcoholic fatty liver disease (NAFLD) | 1944 | 0.000 | 0.000 | 0.001 | 0.006 | 0.000 | 0.000 | 0.428 | 0.496 | 0.000 | 0.000 |
| mmu04933 | AGE-RAGE signaling pathway in diabetic complications | 1434 | 0.000 | 0.000 | 0.007 | 0.029 | 0.000 | 0.000 | 0.000 | 0.000 | 0.000 | 0.000 |
| mmu04934 | Cushing syndrome | 2190 | 0.000 | 0.000 | 0.000 | 0.000 | 0.015 | 0.031 | 0.114 | 0.157 | 0.000 | 0.000 |
| mmu04940 | Type I diabetes mellitus | 558 | 0.000 | 0.000 | 0.263 | 0.437 | 0.000 | 0.000 | 0.135 | 0.181 | 0.267 | 0.289 |
| mmu04950 | Maturity onset diabetes of the young | 288 | 0.315 | 0.379 | 0.018 | 0.064 | 0.087 | 0.142 | 0.000 | 0.000 | 0.000 | 0.000 |
| mmu04960 | Aldosterone-regulated sodium reabsorption | 564 | 0.636 | 0.695 | 0.463 | 0.637 | 0.261 | 0.356 | 0.810 | 0.853 | 0.010 | 0.014 |
| mmu04961 | Endocrine and other factor-regulated calcium reabsorption | 834 | 0.016 | 0.025 | 0.416 | 0.590 | 0.021 | 0.042 | 0.002 | 0.004 | 0.000 | 0.000 |
| mmu04962 | Vasopressin-regulated water reabsorption | 564 | 0.167 | 0.213 | 0.945 | 0.965 | 0.049 | 0.088 | 0.000 | 0.000 | 0.001 | 0.002 |
| mmu04964 | Proximal tubule bicarbonate reclamation | 336 | 0.382 | 0.453 | 0.408 | 0.581 | 0.640 | 0.721 | 0.009 | 0.017 | 0.000 | 0.000 |
| mmu04966 | Collecting duct acid secretion | 300 | 0.880 | 0.900 | 0.318 | 0.492 | 0.000 | 0.000 | 0.003 | 0.007 | 0.001 | 0.002 |
| mmu04970 | Salivary secretion | 1032 | 0.009 | 0.015 | 0.366 | 0.533 | 0.054 | 0.097 | 0.018 | 0.032 | 0.000 | 0.000 |
| mmu04971 | Gastric acid secretion | 1008 | 0.700 | 0.747 | 0.106 | 0.239 | 0.001 | 0.003 | 0.000 | 0.000 | 0.000 | 0.000 |
| mmu04972 | Pancreatic secretion | 1344 | 0.478 | 0.545 | 0.094 | 0.220 | 0.030 | 0.057 | 0.000 | 0.000 | 0.000 | 0.000 |
| mmu04973 | Carbohydrate digestion and absorption | 498 | 0.014 | 0.023 | 0.537 | 0.694 | 0.036 | 0.068 | 0.057 | 0.088 | 0.013 | 0.017 |
| mmu04974 | Protein digestion and absorption | 1020 | 0.000 | 0.000 | 0.000 | 0.000 | 0.310 | 0.405 | 0.029 | 0.049 | 0.000 | 0.000 |
| mmu04975 | Fat digestion and absorption | 420 | 0.010 | 0.017 | 0.001 | 0.006 | 0.391 | 0.476 | 0.000 | 0.000 | 0.000 | 0.000 |
| mmu04976 | Bile secretion | 816 | 0.028 | 0.043 | 0.166 | 0.325 | 0.902 | 0.922 | 0.000 | 0.000 | 0.000 | 0.000 |
| mmu04977 | Vitamin digestion and absorption | 300 | 0.018 | 0.028 | 0.885 | 0.952 | 0.368 | 0.457 | 0.001 | 0.002 | 0.000 | 0.000 |
| mmu04978 | Mineral absorption | 552 | 0.013 | 0.021 | 0.001 | 0.006 | 0.003 | 0.008 | 0.986 | 0.994 | 0.013 | 0.017 |
| mmu04979 | Cholesterol metabolism | 618 | 0.017 | 0.027 | 0.763 | 0.869 | 0.010 | 0.023 | 0.000 | 0.000 | 0.001 | 0.002 |
